# Supplementary material for: RobustiPy: An efficient next-generation multiversal library with model selection, averaging, resampling, and explainable AI
Source: Patterns (N Y). 2026 Aug 14;7(8):101609. doi: 10.1016/j.patter.2026.101609 (PMC13494627; doi:10.1016/j.patter.2026.101609)
Supplement: Supplementary file 1 [file mmc1.pdf]

Patterns, Volume *7*

Supplemental information

RobustiPy: An efficient next-generation multiversal library with model selection, averaging, resampling, and explainable AI

Daniel Valdenegro, Jiani Yan, Duiyi Dai, and Charles Rahal

RobustiPy: An efficient next-generation multiversal library with model selection, averaging, resampling, and explainable AI

Daniel Valdenegro Ibarra[1,3,5], Jiani Yan[1,2,3,4], Duiyi Dai[1,3], and Charles Rahal[1,3,5]

[1]Leverhulme Centre for Demographic Science, University of Oxford
[2]Department of Sociology, University of Oxford
[3]ESRC Centre for Care, University of Oxford
[4]Wolfson College, University of Oxford
[5]Nuffield College, University of Oxford

June 30, 2026

Keywords: *Multiverse analysis*; *Specification curve analysis*; *Robustness analysis*; *Model uncertainty quantification*; *Model selection*; *Bayesian model averaging*; *Bootstrap resampling*; *Joint inference*; *Out-of-sample validation*; *Explainable AI / SHAP*.

For correspondence: Daniel Valdenegro Ibarra, Jiani Yan, and Charles Rahal, Leverhulme Centre for Demographic Science and the Centre for Care, Oxford Population Health, University of Oxford, OX1 1JD, United Kingdom. Tel: 01865 286170. Email: daniel.valdenegro@demography.ox.ac.uk, jiani.yan@demography.ox.ac.uk and charles.rahal@demography.ox.ac.uk. The authors declare no conflicts of interest and are grateful to comments on earlier versions of the work from participants at the International Conference on Computational Social Science, the British Society for Population Studies, the European Consortium for Sociological Research, Population Association of America, Tsinghua University, the International Conference on Social Computing, and internal feedback received from the Leverhulme Centre for Demographic Science, Reproducible Research Oxford, and the Pandemic Sciences Institute. We also thank all attendees at a previous hackathon, hosted at the University of Oxford in June 2024. We especially thank Anda-Raluca Epure for detailed comments on the manuscript and an extensive code review of the accompanying software. The authors are grateful for funding from the ESRC (an ESRC Large Centre grant), the Leverhulme Trust (Grant RC-2018-003) for the Leverhulme Centre for Demographic Science and Nuffield College, and a Grand Union DTP ESRC studentship.

Supplementary Figures

Supplementary Figure S1: Array of never-changing predictors

```
import numpy as np
import pandas as pd
from robustipy.models import OLSRobust


def sim2(project_name):
    np.random.seed(192735)
    beta1 = np.array([0.2, 0.5, -0.4, -0.7, 0.2,
                      0.5, 0.2, 0.5, 0.3])
    L = np.array([
        [0.8, 0.2], [0.6, -0.5], [0.7, 0.1],
        [0.5, -0.6], [0.4, 0.7], [0.3, -0.4],
        [0.2, 0.3], [0.1, -0.2]])
    D = np.diag([5] * 8)
    cov_matrix = L.dot(L.T) + D
    num_samples = 1000
    mean_vector = np.zeros(8)
    X = np.random.multivariate_normal(mean=mean_vector,
                                      cov=cov_matrix,
                                      size=num_samples)
    X_i = np.column_stack((np.ones(num_samples), X))
    errors = np.random.normal(0.0, 1.0, num_samples)
    Y1 = np.dot(X_i, beta1) + errors
    np_data = np.column_stack((Y1, X))
    data = pd.DataFrame(np_data, columns=['y1', 'x1', 'z1', 'z2',
                                          'z3', 'z4', 'z5', 'z6',
                                          'z7'])
    y = ['y1']
    x = ['x1', 'z1']
    z = ['z2', 'z3','z4', 'z5', 'z6', 'z7']
    sim2 = OLSRobust(y=y, x=x, data=data)
    sim2.fit(controls=z, draws=1000, kfold=10, seed=192735)
    sim2_results = sim2.get_results()
    sim2_results.plot(specs=[['z4', 'z5']],
                      ic='hqic', figsize=(16, 16),
                      ext='pdf',
                      project_name=project_name)
    sim2_results.summary()


if __name__ == "__main__":
    sim2('sim2_example')
```

Supplementary Figure S2: Fixed Effect OLS

```
import numpy as np
import pandas as pd
from robustipy.models import OLSRobust


def sim3(project_name):
    np.random.seed(192735)
    beta_mean = [0.9, 0.4, -0.7, -0.1, 0.2, 0.3, -0.2, 0.5, 0.1]
    beta_std = 2
    L_factor = np.array([[0.8,  0.2], [0.6, -0.5],
                         [0.7,  0.1], [0.5, -0.6],
                         [0.4,  0.7], [0.3, -0.4],
                         [0.2,  0.3], [0.1, -0.2]])
    D = np.diag([0.3] * 8)
    cov_matrix = L_factor.dot(L_factor.T) + D
    num_samples, num_groups = 10, 1000
    mean_vec = np.zeros(8)
    X_list, Y_list, group_list = [], [], []
    for group in range(1, num_groups + 1):
        beta = np.random.normal(beta_mean, beta_std, len(beta_mean))
        mi, ma, factor = np.argmin(beta), np.argmax(beta), 5
        beta[mi] = np.random.normal(beta[mi], beta_std * factor)
        beta[ma] = np.random.normal(beta[ma], beta_std * factor)
        X = np.random.multivariate_normal(mean_vec,
                                          cov_matrix,
                                          num_samples) \
            + np.random.normal(0, 5, (num_samples, 8))
        X_i = np.column_stack((np.ones(num_samples), X))
        errors = np.random.normal(0, 20, num_samples)
        Y = np.dot(X_i, beta) + errors
        X_list.append(X)
        Y_list.append(Y)
        group_list.extend([group] * num_samples)
    data = pd.DataFrame(np.column_stack((np.concatenate(Y_list),
                                         np.vstack(X_list))),
                        columns=['y', 'x1', 'z1', 'z2', 'z3',
                                 'z4', 'z5', 'z6', 'z7'])
    data['group'] = group_list
    sim3 = OLSRobust(y=['y'], x=['x1'], data=data)
    sim3.fit(controls=['z1', 'z2', 'z3', 'z4', 'z5', 'z6', 'z7'],
             group='group', draws=1000, kfold=10,
             rescale_y=True, rescale_z=True,
             seed=192735)
    sim3_results = sim3.get_results()
    sim3_results.plot(specs=[['z1', 'z2', 'z3']], ic='hqic',
                      figsize=(16, 16), ext='pdf',
                      project_name=project_name)
    sim3_results.summary()


if __name__ == '__main__':
    sim3('sim3_example')
```

Supplementary Figure S3: Logistic Regression

```
import numpy as np
import pandas as pd
from robustipy.models import LRobust

def sim4(project_name):
    np.random.seed(192735)
    beta1 = np.array([0.05, 0.1, -0.6, -0.35, 0.05,
                      0.1, 0.05, 0.1, 0.05])
    L_factor = np.array([
        [0.8,  0.2],
        [0.6, -0.5],
        [0.7,  0.1],
        [0.5, -0.6],
        [0.4,  0.7],
        [0.3, -0.4],
        [0.2,  0.3],
        [0.1, -0.2]
    ])
    D = np.diag([0.3]*8)
    cov_matrix = L_factor.dot(L_factor.T) + D
    num_samples = 10000
    mean_vector = np.zeros(8)
    X = np.random.multivariate_normal(mean=mean_vector,
                                      cov=cov_matrix,
                                      size=num_samples)
    X_i = np.column_stack((np.ones(num_samples), X))
    errors = np.random.normal(0.0, 1, num_samples)
    Y1 = np.dot(X_i, beta1) + errors
    threshold = np.median(Y1)
    Y1 = (Y1 > threshold).astype(int)
    np_data = np.column_stack((Y1, X))
    data = pd.DataFrame(np_data, columns=['y1','x1','z1','z2',
                                          'z3','z4','z5','z6','z7'])

    y = ['y1']
    x = ['x1']
    z = ['z1','z2','z3','z4','z5','z6','z7']
    sim4 = LRobust(y=y, x=x, data=data)
    sim4.fit(controls=z, draws=1000, kfold=10, seed=192735)
    sim4_results = sim4.get_results()
    sim4_results.plot(specs=[['z1'], ['z2','z4'],
                             ['z3','z5']],
                      ic='hqic', figsize=(16,16),
                      ext='pdf', project_name=project_name)
    sim4_results.summary()


if __name__ == "__main__":
    sim4('sim4_example')
```

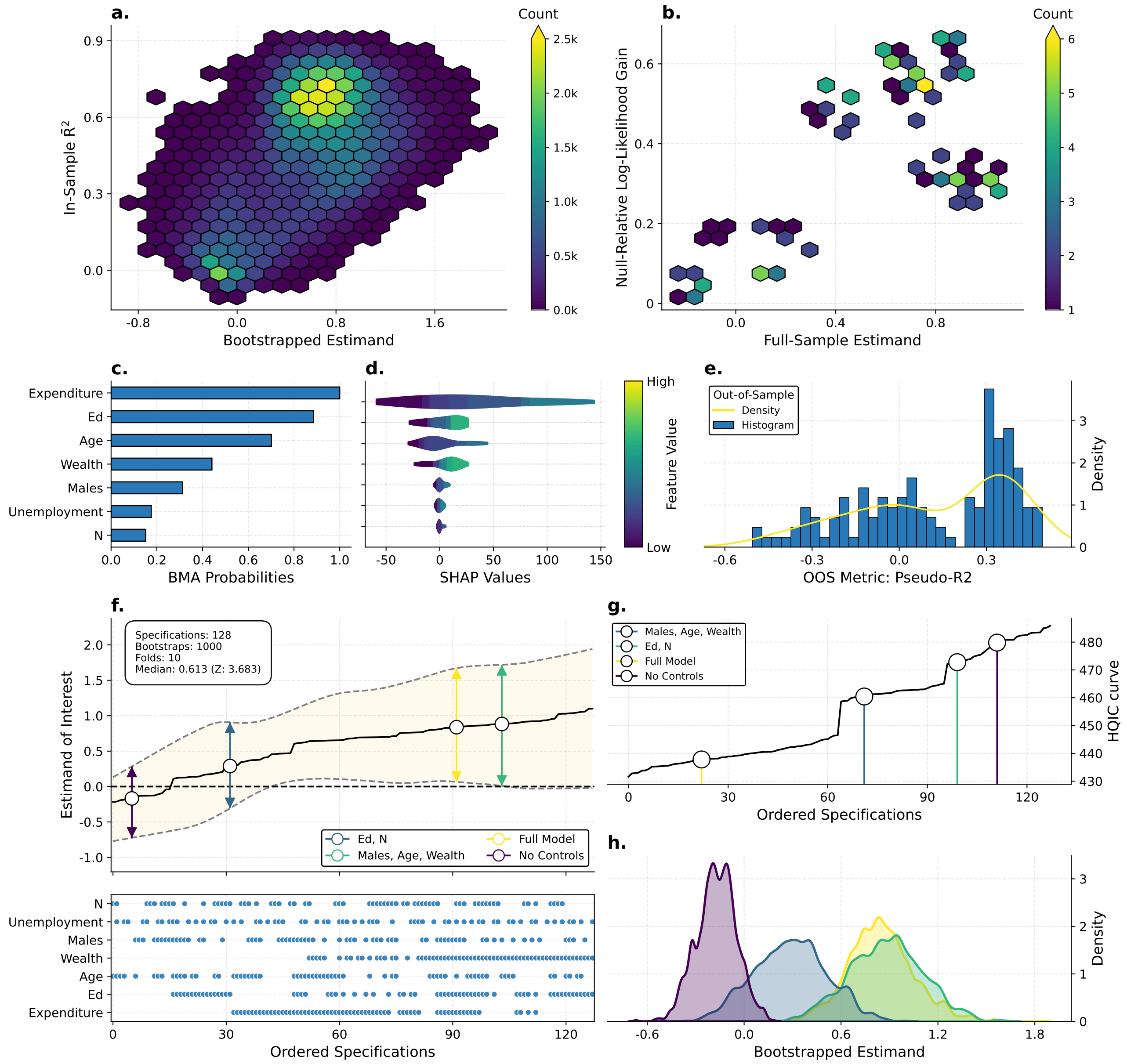

Supplementary Figure S4: An application of `RobustiPy` to criminology: Ehrlich (1973) revisited, based on a revised dataset from Vandaele (1987). The dependent variable is state-level crime rate, while the estimand of interest is income inequality.

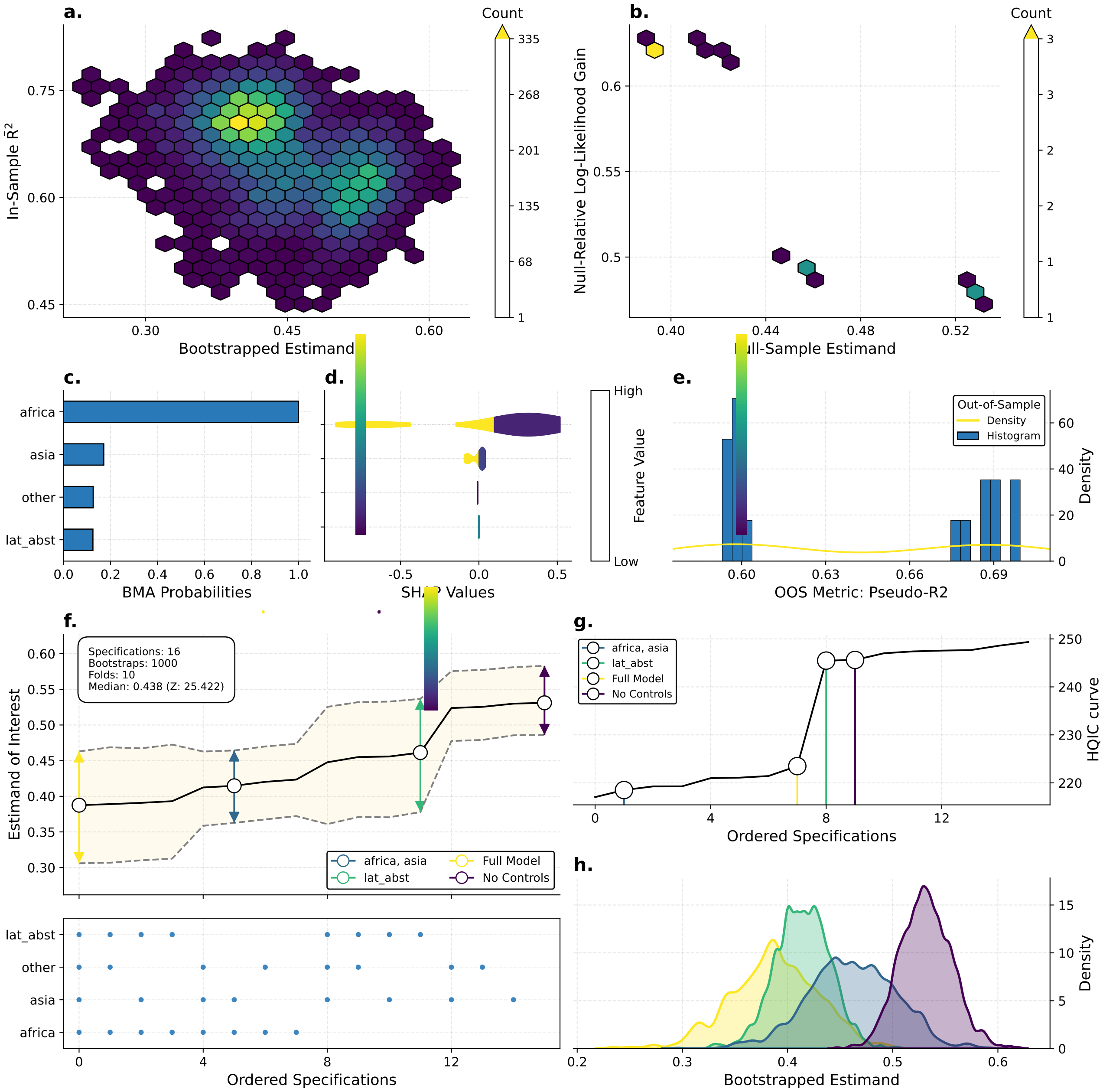

Supplementary Figure S5: A re-examination of the influential Acemoglu et al. (2001) paper. Our analysis spans from the core, baseline OLS model, to the fullest OLS model as specified in Table 2 of the aforementioned paper, where the variation of all intermediate specifications are analysed by `RobustiPy`. The baseline models without any and with all controls are highlighted in subfigures '**f.**', '**g.**' and '**h.**'.

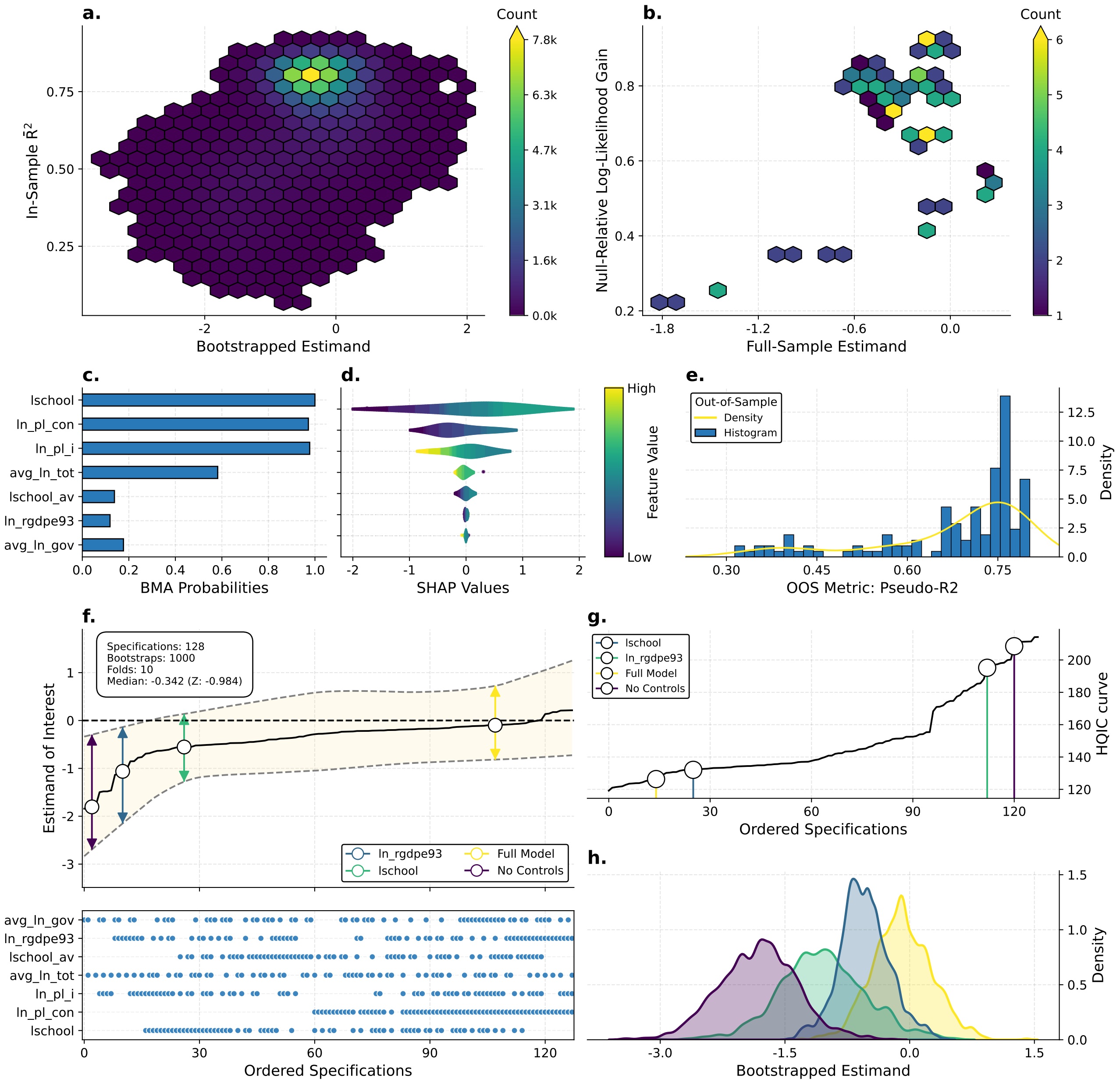

Supplementary Figure S6: A re-analysis of the Solow Growth Model as per Mankiw et al. (1992). With this instantiation of `RobustiPy`, we order the logarithm of (n+g+δ) – averaged over the preceding years and empirically calibrated for all countries – first in our x array.

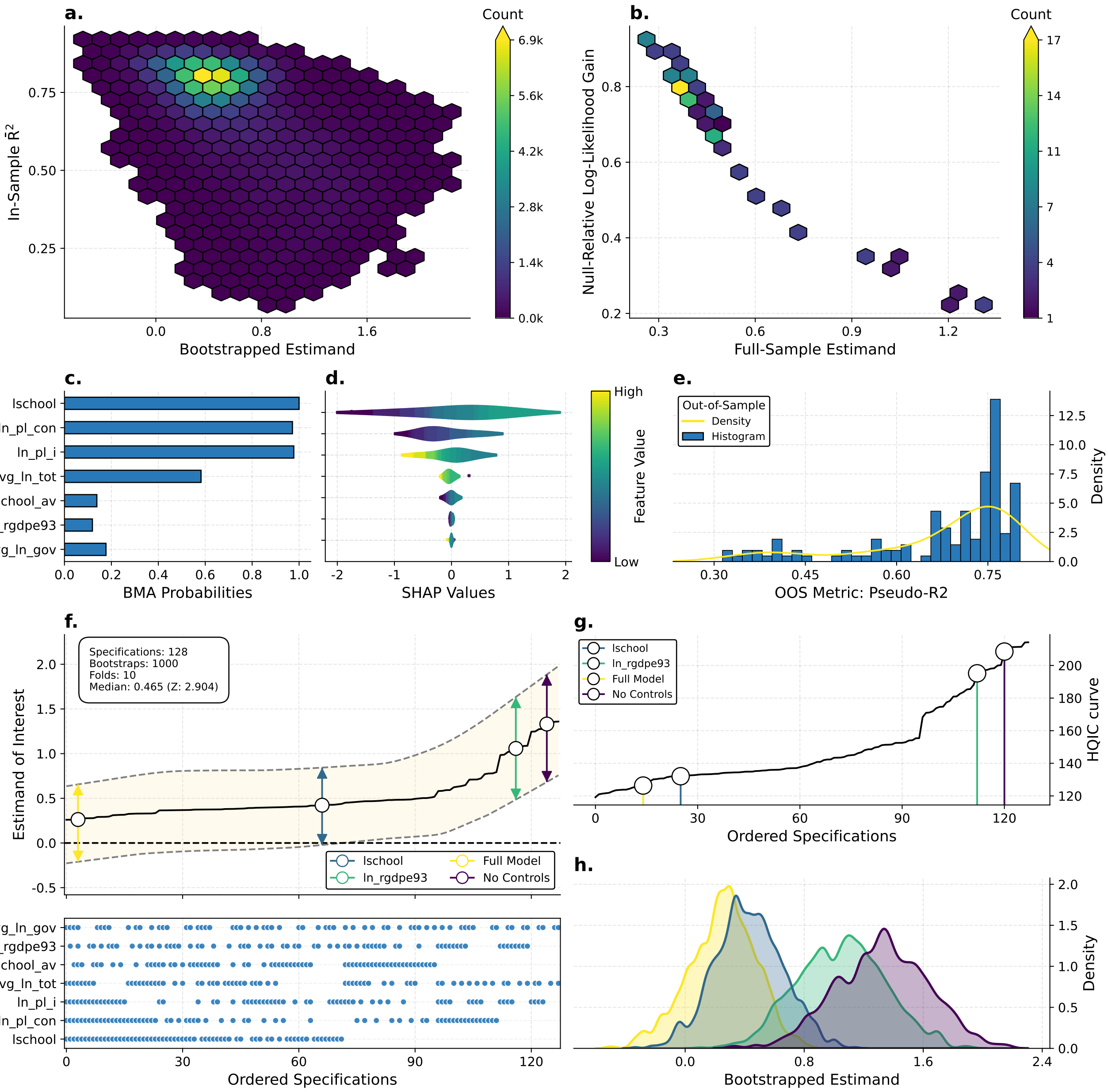

Supplementary Figure S7: A re-analysis of the Solow Growth Model as per Mankiw et al. (1992). With this instantiation of `RobustiPy`, we order the logarithm of $\frac{I}{Y}$ – averaged over the preceding years and empirically calibrated for all countries – first in our x array.

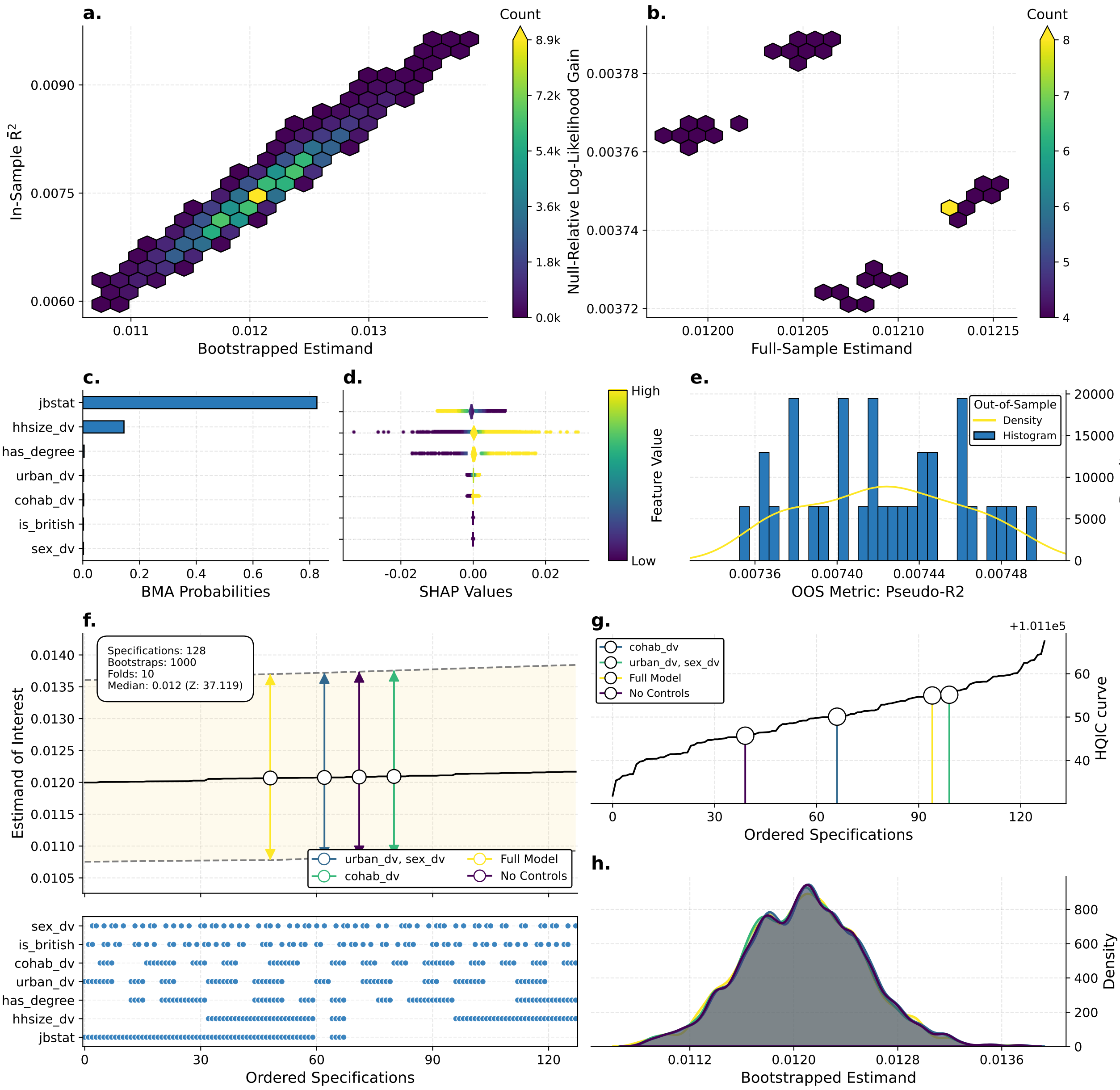

Supplementary Figure S8: Example output of RobustiPy utilising the UKHLS dataset. The dependent variable is a binary representation of whether a survey recipient is in 'good health', estimated as a linear probability model. This example showcases individuals grouped longitudinally via the **group** input to the '`.fit()`' method (in this case, with `group='pipd'`).

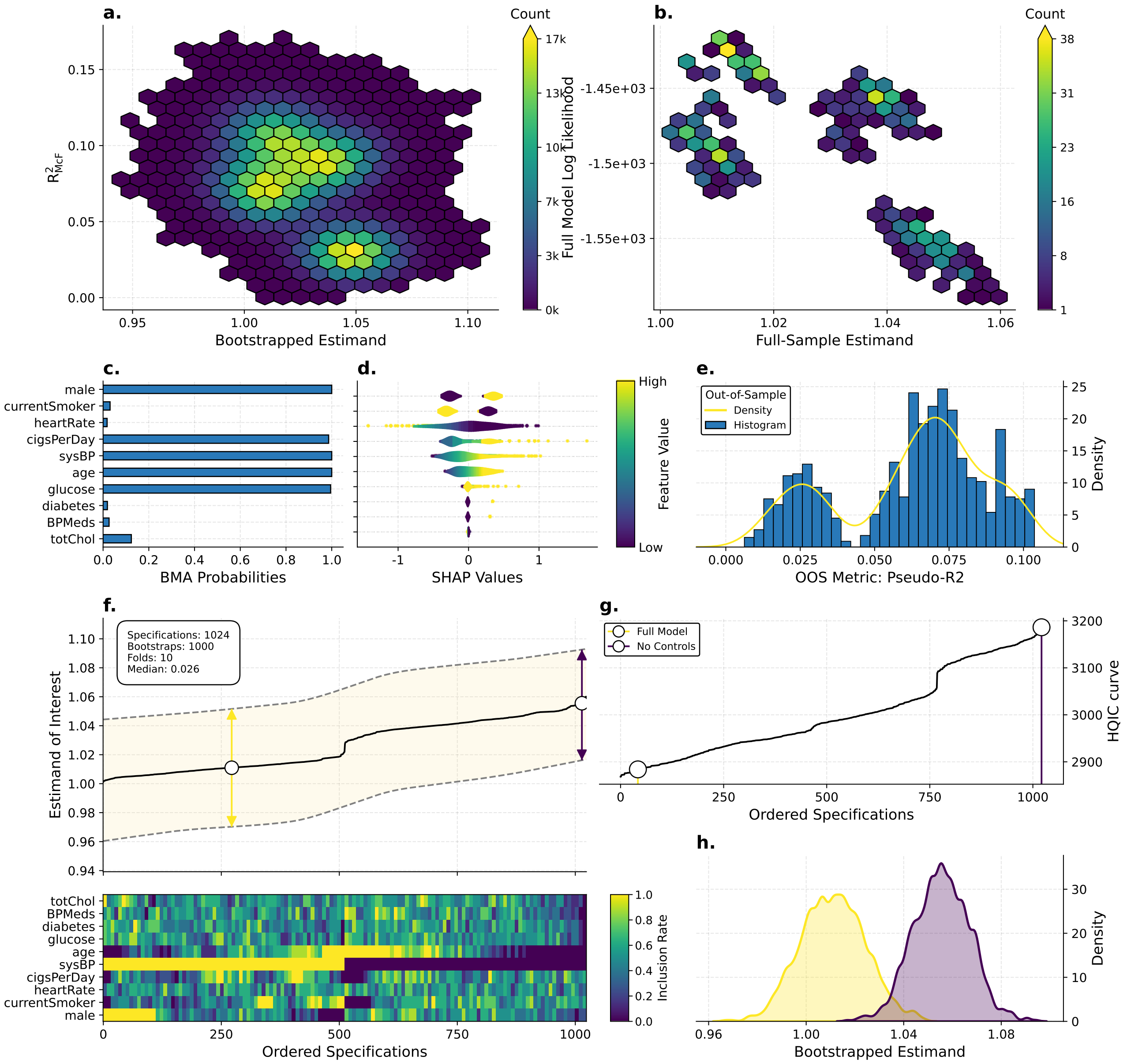

Supplementary Figure S9: Example output of `RobustiPy` with a binary dependent variable. The dependent variable is a ten year metric of coronary heart disease (CHD), and the variable of interest is Body Mass Index.

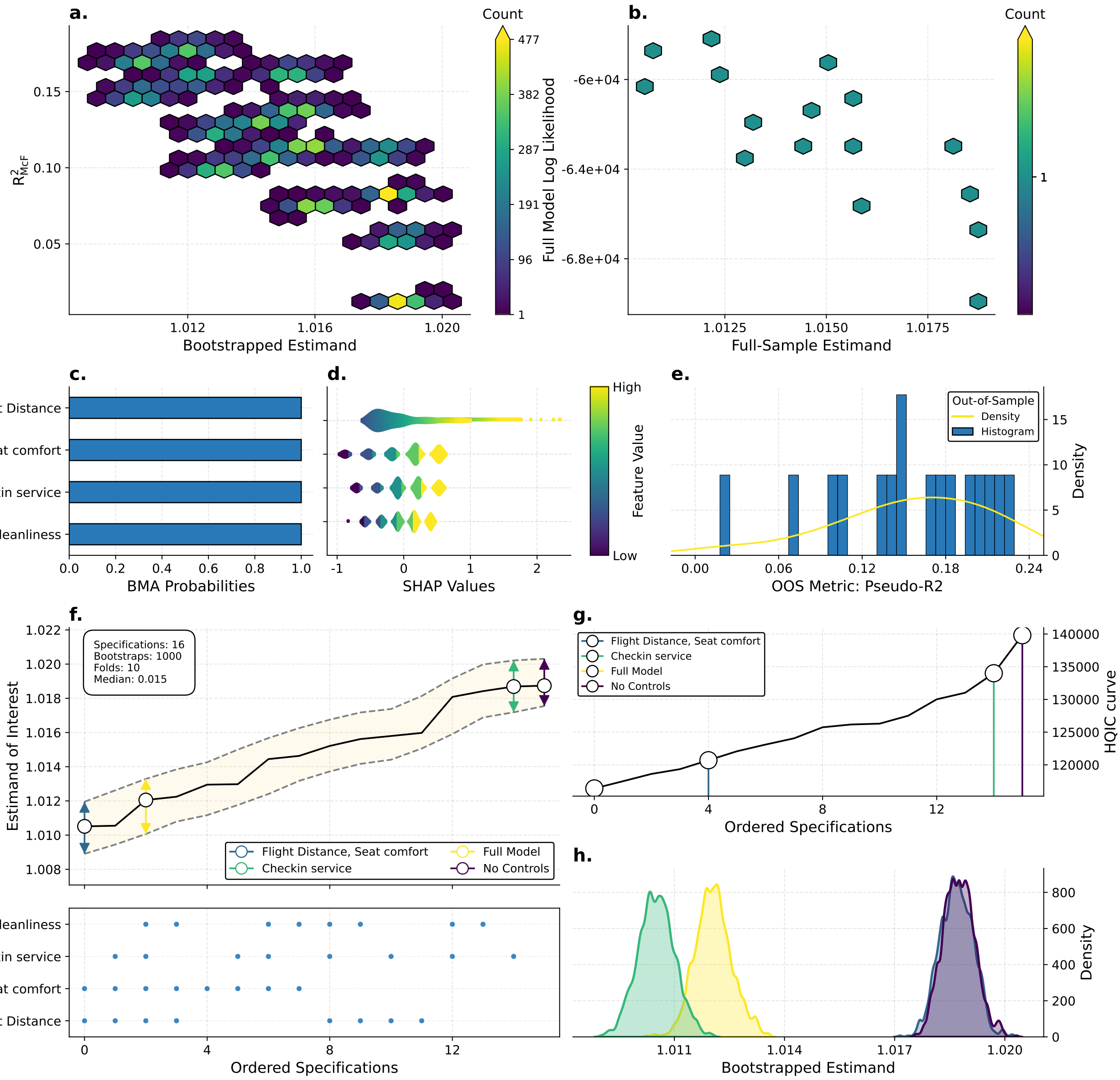

Supplementary Figure S10: Example output of RobustiPy utilising a binary dependent variable. The dependent variable is a representation of custom satisfaction within an anonymous airline company, with data provided by Kaggle. The estimand of interest relates to the age of the customer.

Supplementary References

Daron Acemoglu, Simon Johnson, and James A. Robinson. The colonial origins of comparative development: An empirical investigation. *American Economic Review*, 91(5):1369–1401, 2001.

Isaac Ehrlich. Participation in illegitimate activities: A theoretical and empirical investigation. *Journal of Political Economy*, 81(3):521–565, 1973.

N. Gregory Mankiw, David Romer, and David N. Weil. A contribution to the empirics of economic growth. *Quarterly Journal of Economics*, 107(2):407–437, 1992.

Walter Vandaele. *Participation in Illegitimate Activities: Ehrlich Revisited, 1960 [Data Set]*. Inter-University Consortium for Political and Social Research, 1987.
