# Supplementary material for: RobustiPy: An efficient next-generation multiversal library with model selection, averaging, resampling, and explainable AI
Source: Patterns (N Y). 2026 Aug 14;7(8):101609. doi: 10.1016/j.patter.2026.101609 (PMC13494627; doi:10.1016/j.patter.2026.101609)
Supplement: Supplementary file 2 [file mmc2.pdf]

Patterns

RobustiPy: An efficient next-generation multiversal library with model selection, averaging, resampling, and explainable AI

Graphical abstract

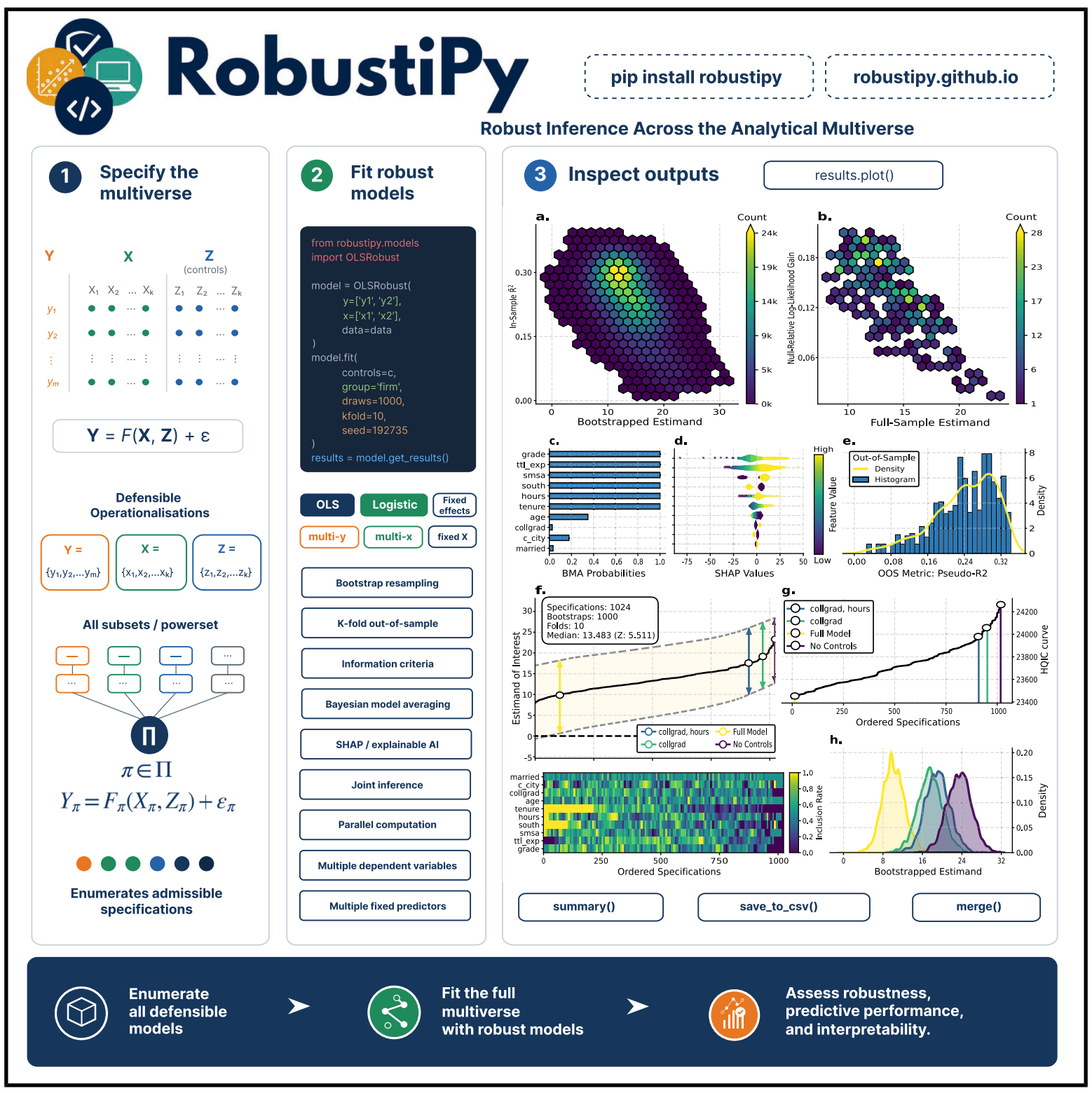

Highlights

- RobustiPy turns defensible modeling choices into explicit analytical multiverses
- It combines bootstrapping, model averaging, joint inference, and explainable AI
- Simulations and empirical examples span diverse research domains
- It is open source (GNU GPL v.3.0), documented, on PyPI, and actively maintained

Authors

Daniel Valdenegro, Jiani Yan, Duiyi Dai, Charles Rahal

Correspondence

daniel.valdenegro@demography.ox.ac.uk (D.V.),
jiani.yan@demography.ox.ac.uk (J.Y.),
charles.rahal@demography.ox.ac.uk (C.R.)

In brief

RobustiPy is a frontier open-source library for asking whether empirical scientific conclusions survive all possible yet defensible modeling choices. It builds analytical multiverses and summarizes uncertainty. Through controlled simulations, time profiling, and empirical replications—including re-analyses of discrepancies in published work—it makes robustness analysis transparent, reproducible, and interpretable.

Valdenegro et al., 2026, Patterns 7, 101609
August 14, 2026 © 2026 The Author(s). Published by Elsevier Inc.
https://doi.org/10.1016/j.patter.2026.101609

CellPress

Patterns

CellPress
OPEN ACCESS

Resource

RobustiPy: An efficient next-generation multiversal library with model selection, averaging, resampling, and explainable AI

Daniel Valdenegro,[1,3,5,*] Jiani Yan,[1,2,3,4,*] Duiyi Dai,[1,3] and Charles Rahal[1,3,5,6,*]
[1]Leverhulme Centre for Demographic Science, University of Oxford, Oxford, UK
[2]Department of Sociology, University of Oxford, Oxford, UK
[3]ESRC Centre for Care, University of Oxford, Oxford, UK
[4]Wolfson College, University of Oxford, Oxford, UK
[5]Nuffield College, University of Oxford, Oxford, UK
[6]Lead contact
*Correspondence: daniel.valdenegro@demography.ox.ac.uk (D.V.), jiani.yan@demography.ox.ac.uk (J.Y.), charles.rahal@demography.ox.ac.uk (C.R.)
https://doi.org/10.1016/j.patter.2026.101609

SUMMARY

Scientific inference is undermined by the vast but rarely explored "multiverse" of defensible modeling choices, which can generate results as variable as the phenomena under study. We introduce RobustiPy, an open-source Python library that systematizes multiverse analysis and model uncertainty quantification at scale. RobustiPy unifies bootstrap-based inference, combinatorial specification search, model selection and averaging, joint inference routines, and explainable AI methods within a modular, reproducible framework. Beyond exhaustive specification curves, it supports rigorous out-of-sample validation and provides feature-level explanations for the full-specification predictive model. We demonstrate utility across five simulations and ten high-profile replications spanning economics, sociology, psychology, and medicine, including a re-analysis of widely cited findings with documented discrepancies. Benchmarking on ∼672 million simulated regressions shows that RobustiPy delivers state-of-the-art computational efficiency while expanding transparency. By standardizing and accelerating methods for robustness, RobustiPy transforms how researchers interrogate sensitivity across the analytical multiverse, offering a foundation for reproducible and interpretable computational science.

INTRODUCTION

The act of constructing quantitative models to examine scientific phenomena represents the cornerstone of most intellectual processes.[1] Yet, the scientific community, particularly within the health and social sciences,[2] has long recognized that the process of testing hypotheses using such models is susceptible to substantial variation in outcomes, even under relatively conservative assumptions.[3] While Leamer's infamous "Let's Take the Con Out of Econometrics" article[4] is often credited with dramatically drawing attention to pervasive risks in applied work, the concept and even formal tools for handling model uncertainty date back decades earlier.[5,6] Numerous factors influence the model-building process, ranging from uncontrollable elements to more structured components like the conditions of data collection; the choices that responsible researchers make over this space are otherwise known as the "garden of forking paths."[7] The range of possible outcomes in scientific modeling may, in fact, mirror the vast variation inherent in reality itself, all when focusing on just one estimand of interest.[8] At the same time, it is increasingly recognized that complex computational routines can themselves be utilized for theory building.[9] We create a tractable solution that takes steps to reduce this consequential source of (epistemic[10]) variation: the analytical choices made by researchers. These choices can, in principle, be systematically examined and controlled. Developed in Python, our readily available, continuously maintained, and highly accessible tool aims to bring transparency to research practice, representing what we believe is the next generation of model uncertainty software. RobustiPy enables efficient large-scale specification curve and multiverse analyses within a unified, reproducible ecosystem; it is also agnostic to philosophies regarding whether models in a multiverse should be weighted[11,12] by considering both the full model space, metrics of fit, estimates selected by minimizing information criteria, and weighted and unweighted estimands. We document the five different types of analyses available through simulated analysis, conduct a time profiling exercise, and apply RobustiPy to ten impactful empirical examples across the research and teaching-related landscape. These empirical examples span the economic, social, psychological,

This is an open access article under the CC BY license (http://creativecommons.org/licenses/by/4.0/).

and medical sciences and beyond. All of our simulated and empirical examples are made available as Python scripts and interactive notebooks within a release of our library, which has been indexed into Zenodo.[13]

Analytical decisions, in comparison to more ethereal, uncontrollable concerns, are not only more amenable to scrutiny but are also potentially more influential for the scientific enterprise. While other sources of variation (such as data collection) may be expected to cancel out with replication, research decisions regarding the modeling of scientific processes are often inherently non-random and systematically biasing. They can introduce systematic biases that remain hidden unless explicitly interrogated. This typically results in an asymmetry of information[14]; current common practice involves researchers varying their decisions, selectively reporting what they choose. Although exploring alternative specifications is not inherently problematic, the persistent lack of transparency in these practices enables questionable research behaviors, such as p-hacking, the process of iteratively modifying models until statistically significant results are obtained.[15,16] A related issue is HARKing (hypothesizing after the results are known; see, for example, Kerr[17]), where researchers test multiple hypotheses and present significant findings as though they had been hypothesized in advance. Beyond concerns about scientific integrity, such selective reporting contributes to poor reproducibility and a misleading sense of empirical consensus, ultimately undermining confidence in research and the scientific endeavor more broadly.[18] A range of solutions have been proposed to address these challenges. These include greater transparency through pre-registration,[19] a stronger emphasis on replication,[20] improved statistical power,[15,21] the adoption of Bayesian inference,[22] and a shift toward predictive modeling as an evaluation tool.[23,24] One particularly promising approach to improving transparency is the systematic reporting of all defensible combinations of analytical decisions made by a researcher. This strategy, independently developed as specification curve analysis[25] and multiverse analysis,[26] explicitly counters selective reporting, the former by looking at the specification-based choices researchers can make and the latter by analyzing a broader choice space. Rather than presenting a single preferred model, it aims to identify and estimate all plausible analysis branches, presenting the full distribution of permissible results in a single, usually visual output. This is what we build upon.

Despite their conceptual appeal, multiverse and specification curve analyses are computationally demanding. Among the many degrees of freedom available to researchers, covariate selection alone can result in an exponential number of model specifications. For example, a dataset with 10 candidate control variables produces $2^{10} = 1{,}024$ possible models; 20 control variables yield over a million. Historically, researchers have tackled this complexity using bespoke computational routines to estimate and summarize the results of thousands,[4] millions,[27] billions,[28] or even trillions[29] of regression models. The expansion of empirical research has brought increased attention to the role of "researcher degrees of freedom"—the wide range of defensible yet subjective decisions made during data collection, model specification, and result reporting,[30] all of which undermine the validity of empirical findings and contribute to broader issues, such as the replication crisis. These problems are pervasive across almost all quantitative academic disciplines, spanning especially psychology,[31] the social sciences,[32] and medical research.[33] However, custom implementations are often inefficient and insufficiently feature rich despite the ever-increasing computational power available to researchers in the limit of human progress.[10,34] To overcome these limitations, we introduce RobustiPy, which streamlines model selection, averaging, out-of-sample validation, and full-model feature explanation while providing uncertainty summaries, inference diagnostics, and fit evaluations across the admissible space of defensible models. By offering a standardized, efficient, and unit-tested solution, RobustiPy enhances computational reproducibility and mitigates the risks associated with costly and limited *ad hoc* implementations of multiverse and specification curve analysis.

Existing state of the art

Several computational tools have been developed to facilitate the implementation of specification curve analysis (a way to visualize all specifications of a model space) and multiverse analysis (the systematic estimation of every defensible analytic pathway) in applied research. In the R programming ecosystem, the multiverse package[35] introduces a declarative syntax for defining multiple decision points within a single analysis script. Users can specify branches corresponding to alternative models and specifications, which are then executed across the entire permissible model space; the package integrates with R Markdown and supports structured workflows for running and reporting multiverse analyses. Specifically regarding specification curve analysis,[25] as opposed to multiversal analysis, the specr package[36] offers a comprehensive set of tools to define, estimate, and visualize a range of model specifications. It is particularly well suited for observational studies, enabling researchers to explore variation in outcomes, predictors, and control variables. Results are presented through specification curves that summarize the distribution of estimates and confidence intervals across the analytical space. Bayesian approaches are also advocated, with code available on the Open Science Framework.[37,38] Similar, but with more limited functionality, spec_chart allows you to create a specification chart using base R.[39] In Stata, there exists the MULTIVRS module to conduct multiverse analysis.[40] There also exists MROBUST, a module to estimate model robustness and model influence.[41]

In contrast, the development of comparable toolkits in the Python ecosystem has been more limited despite the fact that Python has become the world's most widely used programming language for highly performant data science research.[42] The spec_curve package provides foundational functionality for conducting specification curve analyses. While it offers a useful entry point, it lacks the breadth and extensibility of its R-based counterparts. The Boba domain-specific language (DSL)[43] provides a programming language-agnostic tool and visualization system for multiverse analysis. It automatically expands and executes all valid combinations of user decision points. The results are then presented through an interactive, linked-view interface. While Boba supports execution in both R and Python environments, its use of a custom DSL makes it difficult to integrate seamlessly into standard analysis pipelines. RobustiPy extends beyond existing tools by incorporating functionality for model selection, bootstrapping, joint inference, out-of-sample evaluation,

CellPress
OPEN ACCESS

Table 1. Comparison of toolkits for multiverse and specification curve analysis

Feature/toolkit	multiverse (R)	specr (R)	MULTIVRS (Stata)	spec_curve (Python)	Boba (Python-based DSL)	RobustiPy (Python)
Multiverse analysis	✓	–	✓	–	✓	✓ (limited)
Specification curve analysis	–	✓	–	✓	–	✓
High-abstraction syntax	✓	✓ (partial)	✓ (limited)	–	✓	✓
Visualization of results	✓	✓ (built in)	✓ (kdensity)	✓ (basic)	✓	✓ (custom)
Joint inference	–	✓	–	✓	✓	✓
Influence analysis	–	–	✓	–	✓	✓
Bootstrapping	–	✓	–	✓	✓ (partial)	✓
Out-of-sample	–	–	–	–	–	✓
Multiple estimands	–	✓ (limited)	–	✓ (partial)	–	✓

and the systematic exploration of multiple estimands and outcome spaces. In doing so, it enables a more rigorous and transparent approach to robustness analysis. A summary of the main packages in this space is described in Table 1. We consider "multiverse" analysis the analysis that is able to provide outputs using different estimators (e.g., linear regression, logistic regression, probit regression, and so forth).

Formalization

In order to describe the problem RobustiPy addresses, we formalize the problem below. Consider the key association between an outcome **Y** and focal predictors **X**, where a set of control variables **Z** may enter the data-generating process. We write the target regression function as

$$\mathbf{Y} = f^{\star}(\mathbf{X},\mathbf{Z}) + \epsilon,$$

$$\mathbb{E}[\epsilon|\mathbf{X},\mathbf{Z}] = 0. \quad \text{(Equation 1)}$$

Here, $f^{\star}(\mathbf{x},\mathbf{z}) = \mathbb{E}[\mathbf{Y}|\mathbf{X}=\mathbf{x},\mathbf{Z}=\mathbf{z}]$ denotes the target regression function. Equation 1 represents the real data-generating process researchers approximate, but its perfect reproduction is almost always unattainable. Following conventions of previous work,[25] **Y** and **X** are usually imprecisely defined latent variables for which we cannot have direct measures. Likewise, the set of control variables **Z** can also contain imprecisely defined variables; the number of them can be unknown and/or non-finite. Under these conditions, researchers must make concessions, usually in the form of finite sets of "reasonable" operationalizations of **Y**, **X**, **Z** and the functional form. The way "reasonable" is defined is itself a point of contention; prior work[25] often defines this as a set where each element is theoretically justified, statistically valid, and non-redundant. We prefer the term "defensible." Let the defensible operationalization spaces for the outcome, focal predictors, controls, and functional form be denoted by

$$\mathcal{Y}, \mathcal{X}, \mathcal{Z}, \mathcal{F}.$$

A particular specification π selects one admissible element from each relevant space:

$$Y_\pi \in \mathcal{Y},\ X_\pi \in \mathcal{X},\ Z_\pi \in \mathcal{Z},\ f_\pi \in \mathcal{F}.$$

The fitted relation represented by specification π can then be written as

$$Y_\pi = f_\pi(X_\pi, Z_\pi) + \varepsilon_\pi. \quad \text{(Equation 2)}$$

Equation 2 corresponds to a single possible specification π among all admissible specifications in Π. The unrestricted product space is obtained by selecting (1) one composite outcome operationalization, potentially created by one or many components; (2) one functional form or estimator; (3) one focal predictor specification; and (4) one subset of candidate controls. Let

$$m_F := |\Pi_{\mathcal{F}}|,$$

$$m_X := |\Pi_{\mathcal{X}}|. \quad \text{(Equation 3)}$$

If there are d_Z candidate control variables and d_Y candidate components of a composite dependent variable, and the modeling decision is inclusion/exclusion of each control and candidate dependent component with at least one dependent component included, then

$$|\Pi_{\mathcal{Z}}| = 2^{d_Z},$$

$$|\Pi_{\mathcal{Y}}| = 2^{d_Y} - 1. \quad \text{(Equation 4)}$$

Define the unrestricted Cartesian product space as

$$\Pi_{\text{prod}} := \Pi_{\mathcal{Y}} \times \Pi_{\mathcal{F}} \times \Pi_{\mathcal{X}} \times \Pi_{\mathcal{Z}}. \quad \text{(Equation 5)}$$

Its cardinality is

$$|\Pi_{\text{prod}}| = (2^{d_Y} - 1)\, m_F\, m_X\, 2^{d_Z}. \quad \text{(Equation 6)}$$

In practice, not every tuple of choices need be admissible. For example, a binary-response estimator may only be admissible for binary outcomes. Let $C(y, f, x, z)\in\{0,1\}$ denote the corresponding compatibility indicator. The admissible specification space is therefore

$$\Pi = \{(y,f,x,z) \in \Pi_{\mathcal{Y}} \times \Pi_{\mathcal{F}} \times \Pi_{\mathcal{X}} \times \Pi_{\mathcal{Z}} : C(y,f,x,z) = 1\} \subseteqq \Pi_{\text{prod}}. \quad \text{(Equation 7)}$$

Consequently,

$$|\Pi| \le |\Pi_{\text{prod}}| = (2^{d_Y} - 1)\, m_F\, m_X\, 2^{d_Z}, \quad \text{(Equation 8)}$$

with equality only when every cross-combination is admissible. For example, with two functional forms ($m_F = 2$), two target

variables ($d_Y = 2$), two alternative focal predictors ($m_X = 2$), and four candidate controls ($d_Z = 4$), the unrestricted product count is

$$\left|\Pi_{\text{prod}}\right| = \left(2^2 - 1\right) \times 2 \times 2 \times 2^4 = 192. \quad \text{(Equation 9)}$$

If all tuples are admissible, then $|\Pi| = 192$; otherwise, $|\Pi| < 192$. Given that Π represents a collection of admissible approximations to the relationship between **Y** and **X**, enlarging the catalog of admissible specifications can improve coverage of plausible approximations to $f^\star$. This improvement is not automatic; it depends on whether the enlarged catalog contains specifications close to the target function and, when subsampling is used, whether the random sub-selection actually reaches those specifications. The main problem with current research practices is that researchers usually choose a small and non-random sample of Π to represent the variety of results in the full space of possible choices.[25] This space is usually as sparse as a single "baseline" model and a few scant robustness checks.

We formalize the corresponding approximation property as follows. Let $\{\Pi_D : D \in \mathbb{N}\}$ be a nested sequence of admissible specification sets with $\Pi_D \subseteq \Pi_{D+1}$ and cardinality $N_D := |\Pi_D| \to \infty$, where D indexes model complexity or the breadth of the admissible catalog. For each $\pi \in \Pi_D$, let $\widehat{f}_{\pi,n}(x,z)$ denote the fitted prediction rule obtained from a training sample $\mathcal{S}_n$, and define the mean fitted predictor

$$\bar{f}_\pi(x,z); \; = \mathbb{E}_{\mathcal{S}_n}\left[\widehat{f}_{\pi,n}(x,z)\right]. \quad \text{(Equation 10)}$$

Let

$$\mathcal{G}_D; \; = \{\bar{f}_\pi : \pi \in \Pi_D\} \subseteq L_2(P), \quad \text{(Equation 11)}$$

where all L_2 norms are taken with respect to the covariate distribution $P_{X,Z}$:

$$\| g \|_{L_2(P)} = \left(\int g(x,z)^2 \, dP_{X,Z}(x,z) \right)^{1/2}. \quad \text{(Equation 12)}$$

If $\cup_D \mathcal{G}_D$ is dense in $L_2(P)$, then for every $\varepsilon > 0$,

$$\lim_{D \to \infty} \inf_{\pi \in \Pi_D} \| \bar{f}_\pi - f^\star \|_{L_2(P)} = 0. \quad \text{(Equation 13)}$$

This is the full-catalog oracle approximation property.[44,45] When the full catalog is too large to estimate, let $\mathcal{R}_D \subseteq \Pi_D$ denote a random sub-selection. For $\varepsilon > 0$, define the ε-good set

$$G_D(\varepsilon) := \left\{ \pi \in \Pi_D : \| \bar{f}_\pi - f^\star \|_{L_2(P)} < \varepsilon \right\},$$

$$g_D(\varepsilon) := |G_D(\varepsilon)|. \quad \text{(Equation 14)}$$

If $\mathcal{R}_D$ is a simple random sample without replacement of size r_D from Π_D, and

$$\frac{r_D \, g_D(\varepsilon)}{N_D} \to \infty, \quad \text{(Equation 15)}$$

then

$$\Pr_{\mathcal{R}_D}\left[\inf_{\pi \in \mathcal{R}_D} \| \bar{f}_\pi - f^\star \|_{L_2(P)} < \varepsilon \right] \to 1. \quad \text{(Equation 16)}$$

Indeed,

$$\Pr_{\mathcal{R}_D}\{\mathcal{R}_D \cap G_D(\varepsilon) = \varnothing\} = \frac{\binom{N_D - g_D(\varepsilon)}{r_D}}{\binom{N_D}{r_D}} \leq \exp\left\{ - \frac{r_D \, g_D(\varepsilon)}{N_D} \right\}. \quad \text{(Equation 17)}$$

Thus, random sub-selection inherits the oracle approximation property only when it is large enough, relative to the prevalence of good specifications, to hit the approximating region with probability tending to one.

Types of analyses

RobustiPy systematizes exploration of the admissible specification space in Equation 7. In a single fitted object, the estimator class and focal predictor list are fixed, while RobustiPy exhaustively enumerates, or optionally samples, the control subset space $\Pi_{\mathcal{Z}}$. For OLSRobust, additional functionality also constructs and fits non-empty outcome composite subsets $\Pi_{\mathcal{Y}}$ when multiple valid dependent measurements are supplied.

Vanilla computation

Our first simulated example (Figure 1) assumes a linear data-generating process. Here, we fit an OLS estimator with 10-fold cross-validation and 1,000 bootstrap resamples, and then we plot results and print a large number of summary outputs. The data generation process is analogous to a typical modeling problem, whereby a researcher seeks to evaluate the relationship between a target variable of interest with only one valid operationalization and a predictor with only one possible operationalization that is conditional on a "reasonable" set of control variables (and, by extension, a researcher's idea about the data generation process, be it theoretical or otherwise). In this situation (i.e., with no variation in the outcome space, the variable of interest space, or the functional form), only the control subset coordinate varies. More precisely, for fixed Y_0, f_0, and X_0,

$$\Pi_{\text{run}} = \{Y_0\} \times \{f_0\} \times \{X_0\} \times \Pi_{\mathcal{Z}} \cong \Pi_{\mathcal{Z}},$$

where $\cong$ denotes the natural one-to-one correspondence obtained by forgetting the fixed coordinates. For the purposes of delineation, as in Figure 1, the OLSRobust class allows us to declare an object (vanilla_obj) containing the target variable (Y_1), the main predictor as part of an array ($\overleftrightarrow{\mathbf{X}}$), and the source of data. The .fit() method allows us to declare the array of possible control variables ($\overleftrightarrow{\mathbf{Z}}$), which a researcher may be agnostic about including, to be used in the analysis. RobustiPy automatically adds an intercept unless the user has already included one in $\overleftrightarrow{\mathbf{X}}$ or $\overleftrightarrow{\mathbf{Z}}$ (i.e., a feature with any name that has zero variance; columns of zeros are treated as redundant). When a constant is present in $\overleftrightarrow{\mathbf{X}}$, the matrix is left unchanged. A constant placed in $\overleftrightarrow{\mathbf{Z}}$ is treated as a varying regressor (with any constants in $\overleftrightarrow{\mathbf{X}}$ removed) so that the iterator yields some specifications with an intercept and others without. By default,

Vanilla computation

```
import numpy as np
import pandas as pd
from robustipy.models import OLSRobust


def sim1(project_name):
    # 1. Setup
    n = 100
    rng = np.random.default_rng(192735)
    z = [f"z{i}" for i in range(1, 5)]

    # 2. Simulate covariates
    X = rng.standard_normal((n, 1))
    Z = rng.standard_normal((n, len(z)))
    df = pd.DataFrame(np.hstack([X, Z]),
                      columns=["x1"] + z)

    # 3. Generate outcome y1
    df["y1"] = (1 + 2 * df["x1"]
                + 0.5 * df[z].sum(axis=1)
                + rng.standard_normal(n) * 0.5)

    # 4. Fit robust OLS with cross-validation
    vanilla_obj = OLSRobust(y=["y1"],
                            x=["x1"],
                            data=df)
    vanilla_obj.fit(controls=z, draws=1000,
                    kfold=10, seed=192735)

    # 5. Retrieve and plot results
    vanilla_res = vanilla_obj.get_results()
    vanilla_res.plot(specs=[z[1:3]],
                     figsize=(16, 16),
                     ci=0.95, ic="bic",
                     ext="svg",
                     figpath='../figures',
                     project_name=project_name)
    vanilla_res.summary()


if __name__ == "__main__":
    sim1('sim1_example')
```

Figure 1. Vanilla computation

we parallelize computation to decrease runtime. RobustiPy supports interactive prompting for n_cpu. In interactive sessions, if n_cpu is not supplied, then the user can confirm or choose the CPU count; in non-interactive contexts, defaults are applied. Finally, to return the results of fitted models, OLSRobust has a method entitled get_results, returning a class object containing estimates as well as utility functions for summarization and visualization. We allow the user to specify which specifications they wish to visually highlight, allowing direct comparisons with already published results, alongside multiple other options. By default, the "full" model (the one containing all control variables) and the "null" model (which contains only the smallest guaranteed model space) are always highlighted in the relevant visual analysis for the case of one dependent variable. When there is more than one dependent variable specified, it highlights the model with solely the first dependent variable specified in the list and the model that incorporates all dependent and independent variables.

Array of never-changing variables

RobustiPy also allows for the inclusion of multiple "fixed" predictors that are not varied across the covariate space. Programmatically, this set of fixed predictors will be included in all specifications but not considered in the combinatoric operation. They act as a set of always present features. This functionality is useful in cases when the researchers have a strong determination that these variables always need to be included in a model, likely due to theoretical reasoning (or for specific hypothesis testing). It also has the secondary effect of reducing the specification space and, hence, the compute cost, making it an alternative path to follow in cases with very large sets of covariates. To do this, we simply need to append additional predictors into our $\overleftrightarrow{\mathbf{X}}$ array; RobustiPy will take the first feature name of the list as the main estimand of interest and the rest as fixed, never-varying predictors. A stand-alone example of this type of

use case is shown in Figure S1. An alternate approach to reduce compute cost with large control variables space is to pass z_specs_sample_size in OLSRobust.fit(), which samples the covariate space.

Fixed-effects OLS

RobustiPy also provides the option to run a fixed-effects OLS model. These types of models are commonly used in the analysis of panel data, in which repeated observations are nested within the observed unit (e.g., individuals, households, etc.), usually over time. In these contexts, fixed-effects OLS is used to isolate and eliminate the intraclass variation of the observations. RobustiPy allows the specifying of a grouping variable, group, which enters into the .fit() method. By providing this, RobustiPy automatically performs demeaning of the relevant variables and estimates its sets of models on the resultant, transformed data. In the fixed-effects model, the default constant will be removed before any model-fitting, as it contains zero information after demeaning. Grouping identifiers may be string/categorical; numeric encoding is not required. A stand-alone example use case is shown in Figure S2.

Binary dependents

RobustiPy also provides the opportunity to apply a logistic estimator to handle binary outcome variables (via the Newton method[46] and a tolerance of 1×10^{-7}). We chose logistic regression because it is one of the most common and computationally efficient estimators for binary choice models, an important consideration when running specification curves. This functionality is implemented through the LRobust class. It shares the core control subset search, bootstrapping, grouped cross-validation/bootstrap resampling, information criterion summaries, out-of-sample evaluation, plotting interface, and optional Shapley additive explanations (SHAP) explanation workflow with OLSRobust. However, in the current implementation, it supports a single binary outcome per fit, does not construct multiple-outcome composites, does not expose z_specs_sample_size, and does not implement the OLS-style residualized null-bootstrap joint inference routine. When group is supplied to LRobust, it is used for grouped cross-validation and grouped bootstrap resampling, not for within-group demeaning. Naturally, it is also possible to estimate the model space for a binary dependent variable with OLSRobust, which would represent a linear probability model.[47] A stand-alone example use case is shown in Figure S3.

Multiple dependents

Let's now modify our assumed data-generating process by allowing the dependent variable to be represented by a vector of four possible operationalizations. We describe the process to simulate these data in Figure 2. In this scenario, researchers may want to evaluate each admissible control subset $\Pi_{\mathcal{Z}}$ over each outcome operationalization and evaluate all non-empty composite combinations of the outcome operationalizations $\Pi_{\mathcal{Y}}$ in order to obtain the relevant admissible specification space Π. It might also involve an array of never-changing predictors. RobustiPy takes the list of operationalizations and creates combinations of composites by first standardizing their values and then combining them using the row-wise average. Assume m alternative operationalizations of the outcome construct,

$$\mathcal{Y} = \{Y^{(1)}, \ldots, Y^{(m)}\}.$$

1. Compute the Z score of each item:

$$\tilde{Y}_i^{(j)} = \frac{Y_i^{(j)} - \mu_j}{\sigma_j}, \qquad \text{(Equation 18)}$$

where, with $\mathcal{I}_j = \{i : Y_i^{(j)} \text{ is observed}\}$ and $n_j = |\mathcal{I}_j|$,

$$\mu_j = \frac{1}{n_j}\sum_{i \in \mathcal{I}_j} Y_i^{(j)}, \quad \sigma_j^2 = \frac{1}{n_j}\sum_{i \in \mathcal{I}_j}\left(Y_i^{(j)} - \mu_j\right)^2. \qquad \text{(Equation 19)}$$

This matches the population standard deviation convention used by pandas.std(ddof = 0).

2. For every non-empty subset $S \subseteq \{1, \ldots, m\}$, take the row-wise mean:

$$Y_{i,S}^{\text{comp}} = \frac{1}{|S|}\sum_{j \in S}\tilde{Y}_i^{(j)}. \qquad \text{(Equation 20)}$$

3. Each composite Y_S^{comp} then enters a specification-specific model:

$$Y_S^{\text{comp}} = f_\pi(X_\pi, Z_\pi) + \varepsilon_{S,\pi}. \qquad \text{(Equation 21)}$$

This is analogous to Equation 2, with X_π fixed and $Z_\pi \subseteq Z$ chosen by independent-variable specification π. In this particular example, we highlight a specification containing two covariates and the $y1$ and $y2$ composites: ['y1', 'y2', 'z1', 'z2'], and a specification containing only one covariate ($z4$) and a single operationalization, non-composite of the dependent variable in the form of $y4$: ['y4', 'z4']. If researchers are interested in running multiple fit commands—one for each dependent variable without combinations of them—RobustiPy provides concat_results to stack OLSResult objects.

RESULTS

All of the results in the following sections, unless specified otherwise, are reported with 1,000 bootstrapped draws, ten folds, a seed of 192735, and options of the Hannan-Quinn information criteria (HQICs) for model selection and the pseudo-R^2 metric for out-of-sample evaluation. All of these options can be varied, along with additional ones. If draws, folds, seed, out-of-sample metric, or n_cpu are omitted, then RobustiPy prompts for them in interactive sessions; in non-interactive runs, it uses defaults. When there are 128 or fewer specifications, the default subfigure 'F' uses a dot plot; above this threshold, it uses a binned heatmap. We provide the results of ten empirical examples that illustrate the five types of analyses as detailed above; some are in more detail than others in the main text, each with a corresponding notebook as part of our replication materials.[13] All results are tabulated in Table 2.

Multiple dependents

```
import numpy as np
import pandas as pd
from robustipy.models import OLSRobust


def sim5(project_name):
    # 1. Simulation parameters
    n, p = 1000, 5
    sigma_X = np.eye(p)
    rng = np.random.default_rng(192735)

    # 2. Draw covariates X and error epsilon
    X = rng.multivariate_normal(mean=np.zeros(p),
                                cov=sigma_X,
                                size=n)
    epsilon = rng.standard_normal(n)

    # 3. Stack -vectors into a (4xp) matrix
    B = np.array([
        [0.20, 0.50, -0.40, -0.10, 0.20],
        [0.30, 0.40, -0.35, -0.10, 0.20],
        [0.15, 0.60, -0.45, -0.10, 0.20],
        [0.40, 0.30, -0.50, -0.10, 0.20],
    ])  # shape = (4, p)

    # 4. Build DataFrame/generate outcomes and controls
    df = pd.DataFrame(X,
         columns=[f"x{i}" for i in range(1, p + 1)])
    df[[f"y{j}" for j in range(1, 5)]] = (
        X @ B.T + epsilon[:, None])
    df[[f"z{j}" for j in range(1, 5)]] = (
        rng.standard_normal((n, 4)))

    # 5. Specify outcome and control names
    Y = [f'y{j}' for j in range(1, 5)]
    z = [f'z{j}' for j in range(1, 5)]

    # 6. Fit robust OLS
    model = OLSRobust(y=Y, x=['x1'], data=df)
    model.fit(controls=z, draws=1000,
              kfold=10, rescale_y=True,
              rescale_x=True, rescale_z=True,
              seed=192735)

    # 7. Retrieve and plot results
    res = model.get_results()
    res.plot(loess=False,
        specs=[['y1', 'y2', 'z1', 'z2'],
               ['y4', 'z4']],
        figsize=(16, 8),
        figpath='../figures',
        project_name=project_name)
    res.summary()

if __name__ == "__main__":
    sim5('sim5_example')
```

Figure 2. Multiple dependents

Vanilla computation

Our first example utilizes the infamous union.dta dataset, a subsample of the National Longitudinal Survey of Young Women.[55] This dataset is anonymized and publicly available. It contains information on things like wage (which, when logarithmically transformed, represents the dependent variable, often written as y_i), union membership (and the associated estimand of interest, $\widehat{\beta}_1$ in the canonical reduced form linear regression), and a multitude of often arbitrarily specified controls. Results are visualized in Figure 3. A simple OLS regression on the full sample yields an estimand of interest of 10.18, lower than "conventional estimates," which are nearer to a 15% premium.[56] Other work[41] estimates the (unweighted) median effect across the multiverse as 14.00; the median of our unweighted estimates across all samples with no bootstraps is 13.5 and across all samples and bootstraps as 13.5, with a Bayesian information criterion (BIC)-weighted estimate of 9.8. We are agnostic as to which number is most statistically valid, simply providing the tools by which a researcher can consider the range of plausible results.

We also undertake a "vanilla" application to criminology: a revisitation[49] of a highly contentious paper,[57] where the dependent variable is state-level crime rate, and the estimand of interest is income inequality (defined as the percentage of families earning below one-half of the median income). The results from the .summary() method, inherent to RobustiPy, are shown in Figure 4 and Figure S4. This example is particularly interesting because, as shown in other supplemental information figures, the direction of the estimand of interest actually changes across specification choice (e.g., from a minimum of -0.8743 to a maximum of 2.0337). Figure S5 contains a re-analysis of the "colonial origins of comparative development" based on other influential work.[48]

Array of never-changing variables

We next detail the case where we have an array of predictor names we always want to include in the model space but that never vary. In terms of RobustiPy functionality, this simply involves passing an array of predictor names ($\overleftrightarrow{\mathbf{X}}$) that has length greater than 1, with the array of variable controls specified as before ($\overleftrightarrow{\mathbf{Z}}$). This involves using updated variables from the Penn World Table (version 10.01[58]) to revisit existing work[50] which contributes to the "empirics of economic growth." We map modern data from 73 of the 98 countries in the original 1988 sample, using observed values of all parts of the $log(n+g+\delta)$ and $\log\left(\frac{I}{Y}\right)$, which are canonical to the Solow growth model. We also include various possible extensions,[50] such as logged metrics of human capital, and previous values of GDP in levels. By running RobustiPy twice—with $\overleftrightarrow{\mathbf{X}} = \left[\log(n+g+\delta), \log\left(\frac{I}{Y}\right)\right]$ and $\overleftrightarrow{\mathbf{X}} = \left[\log\left(\frac{I}{Y}\right), \log(n+g+\delta)\right]$, both of which we always want in our core model—we are able to recover two large sets of bootstrapped estimates of the relevant estimands accompanying the first variable in each of these two arrays. This allows us to extract estimates from the results object of RobustiPy to assess whether the signs are of equal size and opposite magnitude. RobustiPy also allows us to visualize the distribution of the $\overline{R}^2$ values (a key test of the Solow growth model); both are shown in Figure 5, with full RobustiPy outputs for the two runs shown in Figures S6 and S7.

Fixed-effects OLS

Regarding fixed-effect estimation, we replicate work that takes a longitudinal approach to local government spending on adult social care and carers' subjective well-being in England.[51] Specifically, we analyze the effect of a local authority measure of adult social care spending on a general health questionnaire (GHQ12) metric from the United Kingdom Household Longitudinal Survey. A static group of predictors (i.e., inside $\overleftrightarrow{\mathbf{X}}$) includes seven sociodemographic and health variables alongside 13-year fixed effect dummy variables, whereas our array of controls—which do vary—contains a further eight variables. Importantly, we document the inclusion of a grouping variable (pipd), common in longitudinal survey analysis. Figure 6 shows the results, with the median value of the estimand of interest at 0.2069 for all specifications and all bootstrapped resamples, 0.2016 for all specifications and no resampling, and 0.1825, 0.1818, and 0.1826 for Akaike information criterion (AIC), BIC, and HQIC weighted estimates, respectively. This information is contained in an unreported printout analogous to Figure 4. This result is extremely close to that reported in the original paper (0.1825). Given that the size of this feature space is so large, had we not specified a larger $\overleftrightarrow{\mathbf{X}}$ (an array of always present predictors), RobustiPy would have had to estimate a relatively intractable control inclusion space of $2^{d_Z} = 268,435,456$ specifications, where $d_Z = 28$ is the number of candidate control variables. We could have alternatively used our method of resampling Π_Z. A complementary pedagogical example with the Understanding Society Longitudinal Teaching dataset (waves 1–9)[59] is provided in Figure S8, where the estimand of interest is sf1_dv (general health) and a fixed effect grouping is based on a unique cross-wave person identifier.

Binary dependents

We next describe an example of how RobustiPy can be used when our dependent variable is binarized as part of an application common to the medical sciences. In particular, we utilize the Framingham Heart Survey[52] for the estimation of models related to a dependent variable termed TenYearCHD: a binary indicator equal to 1 if a study participant experienced a coronary heart disease (CHD) event within 10 years of their baseline examination and zero otherwise. This is used in multiple high-profile papers[60] and as part of a composite in others.[61] The dataset includes over 4,240 records across 16 features. We fit our LRobust class with TenYearCHD as our target dependent and BMI as the single element of $\overleftrightarrow{\mathbf{X}}$ for which we calculate estimands of interest, and we use age, male, totChol, sysBP, BPMeds, currentSmoker, cigsPerDay, diabetes, heartRate, and glucose as elements of our array of controls ($\overleftrightarrow{\mathbf{Z}}$). This example accepts the odds ratio Boolean into the .plot() command on the results object to calculate odds ratios, with results shown in

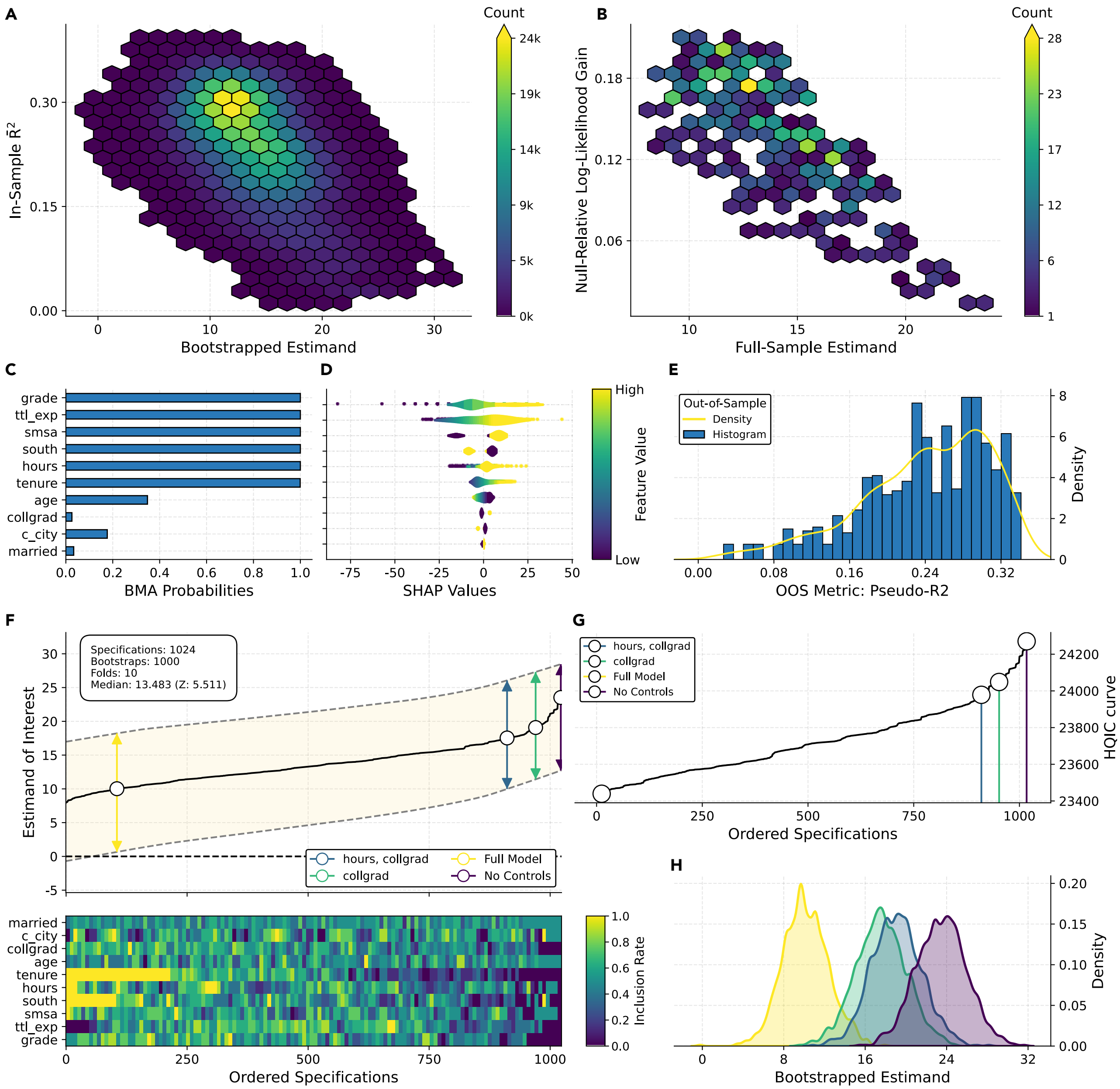

Figure 3. Example output of RobustiPy utilizing the union.dta dataset.

(A) In-sample $\overline{R}^2$ fit against bootstrapped estimands.

(B) The full-sample, non-bootstrapped estimands plotted against the null-relative log likelihood gain (per observation) of the corresponding OLS models.

(C) BIC-implied posterior inclusion probabilities for the candidate controls and (D) SHAP values, sharing *y* axis tick labels.

(E) Distribution of the chosen out-of-sample metric.

(F) For each specification, the median bootstrap estimate is shown in solid black. The dashed gray lines show LOESS-smoothed curves through the minimum and maximum bootstrap estimates (the default ci = 1 setting), with the intervening range shaded in yellow. The lower subpanel shows a binned heatmap of control-variable inclusion rates across the ordered specifications.

(G) Model selection.

(H) Distribution of bootstrapped estimand.

The coloring of specific lines, distributions, and markers in (F)–(H) represent the position of highlighted specifications of interest; RobustiPy will always show the position of the model with all variables (full model) and the model with no control variables beyond the variable of interest (no controls). The colormap can also be user specified.

Figure S9. Our online replication archive also includes models fit to a Kaggle dataset on a binarized measure of airline customer satisfaction (from an undisclosed airline); the resulting coefficient estimates indicate how RobustiPy can be used as a method for model selection and averaging and is presented in Figure S10 and, again, summarized in Table 2 (which also contains

Summarizing a re-analysis of criminality

```
==============================
1. Model Summary
==============================
Model: OLS Robust
Inference Tests: Yes
Dependent variable: R
Independent variable: Inequality
Number of possible controls: 7
Number of draws: 1000
Number of folds: 10
Number of specifications: 128
==============================
2.Model Robustness Metrics
==============================
2.1 Inference Metrics
==============================
Median β (specs, no resampling): 0.6976 (null-calibrated p: 0.001998)
Median β (bootstraps x specs): 0.6134
Min β (specs, no resampling): -0.2304
Min β (bootstraps x specs): -0.8743
Max β (specs, no resampling): 1.0851
Max β (bootstraps x specs): 2.0337
AIC-weighted β (specs, no resampling): 0.7754
BIC-weighted β (specs, no resampling): 0.7296
HQIC-weighted β (specs, no resampling): 0.7588
Share significant (specs, no resampling) [descriptive]: 0.7500
Share significant (bootstraps x specs) [descriptive]: 0.7293  (p: 0.001998)
Share β>0 (specs, no resampling) [descriptive]: 0.8750
Share β>0 (bootstraps x specs) [descriptive]: 0.8632 (p: 0.2498)
Share β<0 (specs, no resampling) [descriptive]: 0.1250
Share β<0 (bootstraps x specs) [descriptive]: 0.1368  (p: 0.2498)
Share β>0 & significant (specs, no resampling) [descriptive]: 0.7500
Share β>0 & significant (bootstraps x specs) [descriptive]: 0.7141 (p: 0.001998)
Share β<0 & significant (specs, no resampling) [descriptive]: 0.0000
Share β<0 & significant (bootstraps x specs) [descriptive]: 0.0152 (p: 0.001998)
Stouffers Z = 3.6834  (two-sided p = 0.000999)
==============================
2.2 In-Sample Metrics (Full Sample)
==============================
Min AIC: 427.4263, Specs: ['Expenditure', 'Wealth', 'Ed', 'Age']
Min BIC: 438.5272, Specs: ['Expenditure', 'Wealth', 'Ed', 'Age']
Min HQIC: 431.6036, Specs: ['Expenditure', 'Wealth', 'Ed', 'Age']
Max Relative Log-Likelihood Gain (per obs): 0.664, Specs: ['Expenditure', 'Males', 'N',
                                                           'Age', 'Unemployment', 'Ed',
                                                           'Wealth']
Min Relative Log-Likelihood Gain (per obs): 0.0163, Specs: []
Max Raw Log Likelihood: -206.7716, Specs: ['Expenditure', 'Males', 'N', 'Age',
                                           'Unemployment', 'Ed', 'Wealth']
Min Raw Log Likelihood: -237.2149, Specs: []
Max Adj-R2: 0.6922, Specs: ['Expenditure', 'Males', 'Age', 'Ed', 'Wealth']
Min Adj-R2: -0.0310, Specs: ['Unemployment', 'Age']
==============================
2.3 Out-Of-Sample Metrics (pseudo-r2 averaged across folds)
==============================
Max Average: 0.4925, Specs: ['Expenditure', 'Unemployment', 'Ed', 'Age']
Min Average: -0.5057, Specs: ['Males', 'Unemployment', 'Ed', 'Age']
Mean Average: 0.12
Median Average: 0.2026
```

Figure 4. Summarizing a re-analysis of criminality

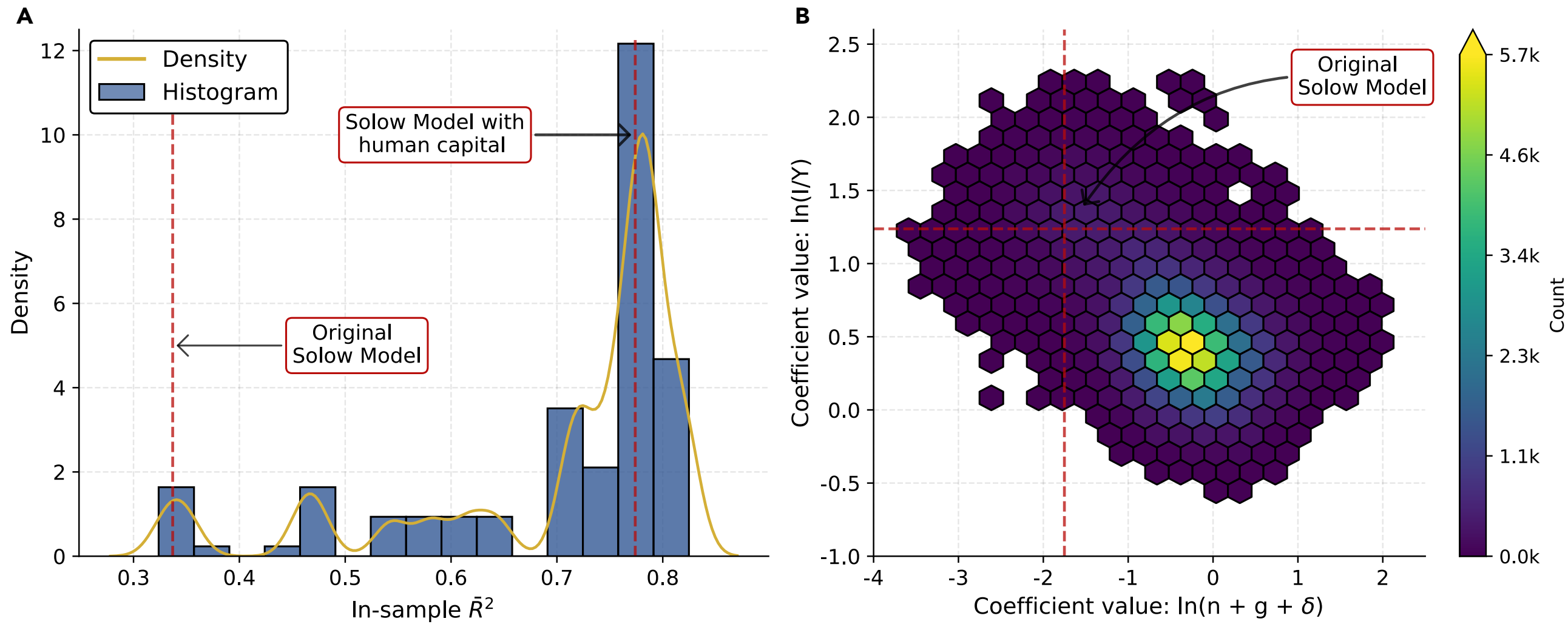

Figure 5. Replicating and extending Solow growth models with RobustiPy

(A) The distribution of $\bar{R}^2$ is identical for each of the two orderings of $\overleftrightarrow{X}$, as the model space is otherwise identical across the two results objects.

(B) A composite of two runs of RobustiPy, with the two reordered $\overleftrightarrow{X}$ arrays.

The figure illustrates the substantive finding of prior work[50]; the basic Solow model without human capital exhibits systematically lower explanatory power, while augmenting the model with a human capital proxy yields markedly higher $\bar{R}^2$ values. The hexbin representation in (B) makes clear that the coefficients vary enormously in comparison to the small number of values reported by the original study, which we replicate and extend.[50]

RobustiPy results from estimating the problem as a linear probability model; i.e., with the OLSRobust class).

Multiple dependents

The most common case of multiple target variables arises from the use of multi-item measures in surveys. Here, we attempt to replicate the results of an influential piece of research[62] alongside further replication analysis conducted by DataColada for the same study. In their analysis,[63] DataColada alluded to the manipulation of results through the alteration of the data used in the originally published data and analyses, ultimately leading to a retraction of the original study.[53] Here, we attempt to replicate the effect of the experimental condition on measures of moral impurity (seven items) and networking intentions (four items), separating the datasets used in both the originally published study and that which has been reconstructed.[64] In the case of multiple dependent measures, RobustiPy combines them into all possible composite combinations (i.e., average of the standardized Z scores of the measures), including single-item combinations. To ensure comparability between items, RobustiPy uses Z scores rather than raw measures. Finally, to replicate an ANOVA/analysis of covariance (ANCOVA), we convert the categorical column containing the experimental condition group membership into dummy variables and use them as predictors in each case. The results are shown in Figure 7. RobustiPy is there used to plot specification curves based on the published dataset, which would appear to support the main hypothesis of the study, as it shows that any combination of the outcome variable "moral impurity" has a significant effect from the experimental condition. However, results using the reconstructed data show that the effect is much weaker and in the opposite direction than hypothesized. This example illustrates the flexibility of RobustiPy, demonstrating how it can be used to replicate both ANOVA and ANCOVA analyses even though these are not explicitly implemented in the library while also enabling the integration of custom visualizations to present results. It also emphasizes the capacity of RobustiPy to act as a tool for replication. More broadly, this example shows how RobustiPy can be employed as a tool for auditing existing studies by systematically exploring the robustness of published findings. By taking the output of the .get_results() object, RobustiPy enables a multitude of custom comparisons beyond that which a single run permits. We also use RobustiPy to replicate other work[54] that aims to understand the "association between adolescent well-being and digital technology use." In this article, data from well-known adolescent well-being panel studies are used across a series of measures of well-being (dependent variables) and digital use (independent variables). Under this setting, the authors opt for a specification curve analysis approach to obtain a distribution of the effect of many forms of technology use across many measures of well-being; we replicate one of the main analyses, where the results—which are also a function of RobustiPy's ability to rescale $\overleftrightarrow{\mathbf{X}}$ and $\overleftrightarrow{\mathbf{Z}}$—can be seen in Figure 8 and Table 2.

DISCUSSION

Table 2 outputs all results from our 10 empirical replications. It explicates the enormous variation in both the estimand of interest across a researcher's set of reasonable choices and the variation in out-of-sample accuracy across specification spaces. It also shows, for example, how we can consider whether coefficient values are approximately equal and

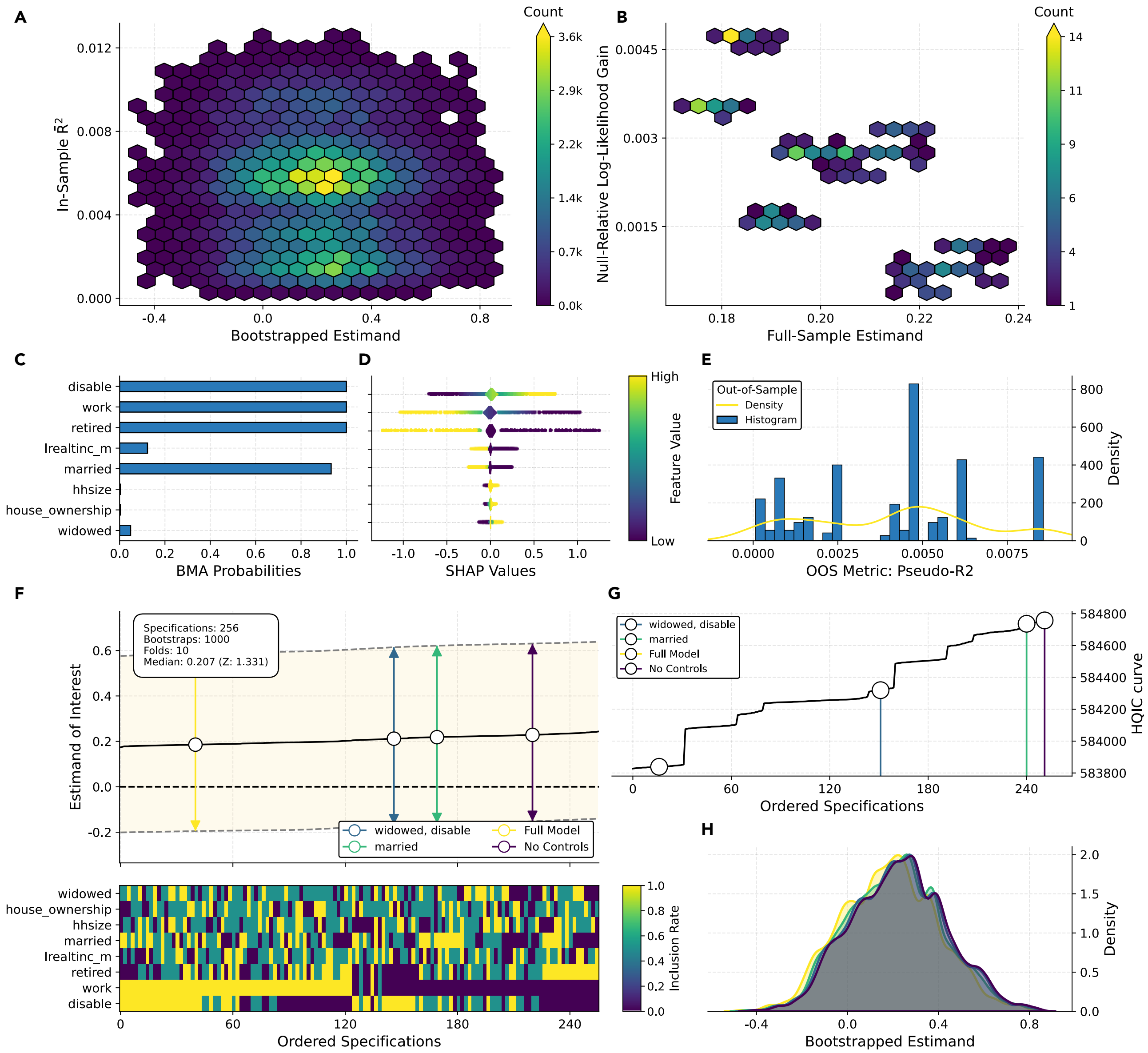

Figure 6. Example output of RobustiPy with both a grouping variable and an array of never-changing variables

The dependent variable is a measure of general health (GHQ12), and the estimand of interest ($\hat{\beta}$) is associated with spending on adult social care.

opposite across various different realizations of an estimand (weighted or unweighted). It emphasizes the ability of RobustiPy to act as a tool for the evaluation of research already published in the scientific record, not least through its ability to highlight specific specifications of interest in the visualization; it acts as an essential audit tool when the validity of research is under question.[53] While not emphasized elsewhere in this manuscript, RobustiPy reports the extrema of selected out-of-sample evaluation metrics and identifies the specifications that minimize selected information criteria; this functionality is useful for comparing predictive fit and fit-complexity trade-offs across the admissible model space (see Figure 4 for an example). For score-type out-of-sample metrics such as pseudo-R^2, McFadden's-R^2, and InterModel Vigorish (IMV), larger values indicate better performance; for loss-type metrics, such as RMSE and cross-entropy, smaller values indicate better performance. While Table 2 is compiled from the .summary() method, this is just one of the three ways by which RobustiPy provides output; it also provides a large number of (separable) visualizations alongside *all* raw estimates (which can be used for bespoke downstream analyses).

We also time profile RobustiPy. It involves two simple simulations of data-generating processes[13] and contains an example of each of both the OLSRobust and LRobust classes. Let B denote the number of bootstrap draws, K the number of cross-validation folds, and $M = |\Pi|$ the number of specifications. In these timing experiments, B varies over 25 integer values on a log scale between 10 and 10,000, $K \in \{2,5,10,15,20,25\}$, and the

CellPress
OPEN ACCESS

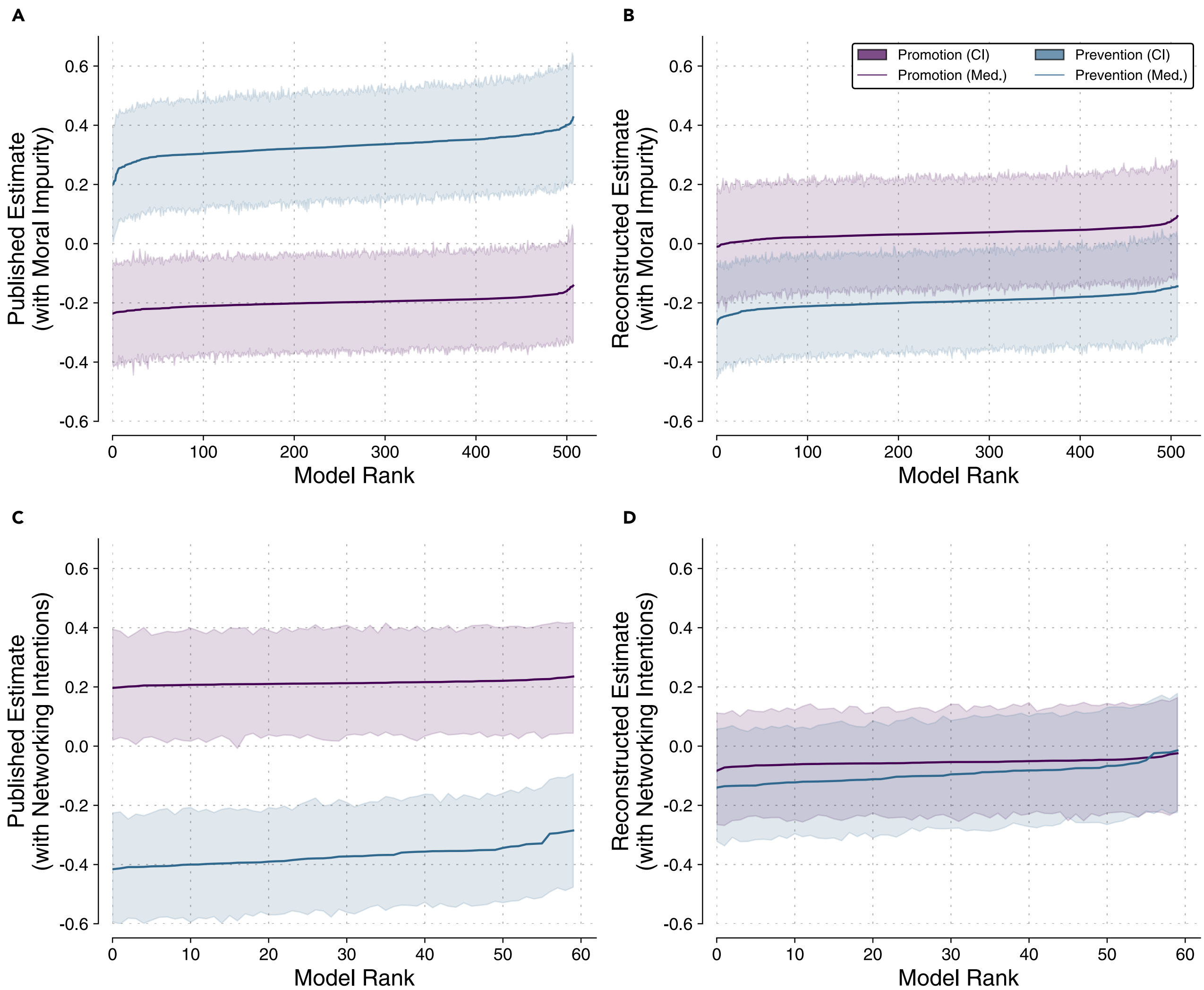

Figure 7. Output of OLSRobust using multiple dependent variables

In this example, we use a custom plotting function that accepts input from the output results object of the OLSResult class, comparing multiple competing versions of Π. The "promotion" and "prevention" conditions refer to the experimental conditions of the original study.

number of candidate controls ranges from 3 to 7, giving $M \in \{2^3, \ldots, 2^7\}$ specifications in the vanilla control selection setting. The profiler is parameterized over these draws, folds, and control set sizes. The plotted benchmarking results are shown in Figure 9. Using the accounting convention,

$$N_{\text{fits}} = 3R \sum_{r \in \mathcal{R}_{\text{plotted}}} 2^{d_r}(B_r + K_r),$$

where $R = 10$, d_r is the control set length, B_r is the number of bootstrap draws, K_r is the number of folds, and $\mathcal{R}_{\text{plotted}}$ is the set of completed timing rows used in the figure, this gives 671,512,080 regression-equivalent fits. The seed is unchanged for each iteration, and the current profiling script uses n_cpu = 24 on a 14900F processor, 96 GB of DDR5 random access memory with Python 3.12 installed on an Ubuntu 25.10 operating system. Ignoring parallelization overhead, let $C_{\text{OLS}}(n,p)$ and $C_{\text{logit}}(n,p)$ denote the cost of fitting one OLS and logistic model, respectively. For OLSRobust with the residualized null-bootstrap joint inference routine enabled, the dominant computational cost scales approximately as

$$O(M(2B + K + 1)C_{\text{OLS}}(n,p)),$$

because each specification requires the full-sample fit, K cross-validation fits, B ordinary bootstrap fits, and B residualized null-bootstrap fits. Without the null-bootstrap joint inference component, the corresponding leading term is

$$O\left(M(B+K+1)C_{logit}(n,p)\right).$$

We aim to provide an accessible tool for researchers to run the most common associative models in an era when computational power is vast. We offer this easy-to-use interface in the hope that transparent results reporting will spread and become the default standard for statistical analysis. By lowering the user experience barrier, we aim to accelerate the adoption—and routine practice—of robust modeling. We include as many

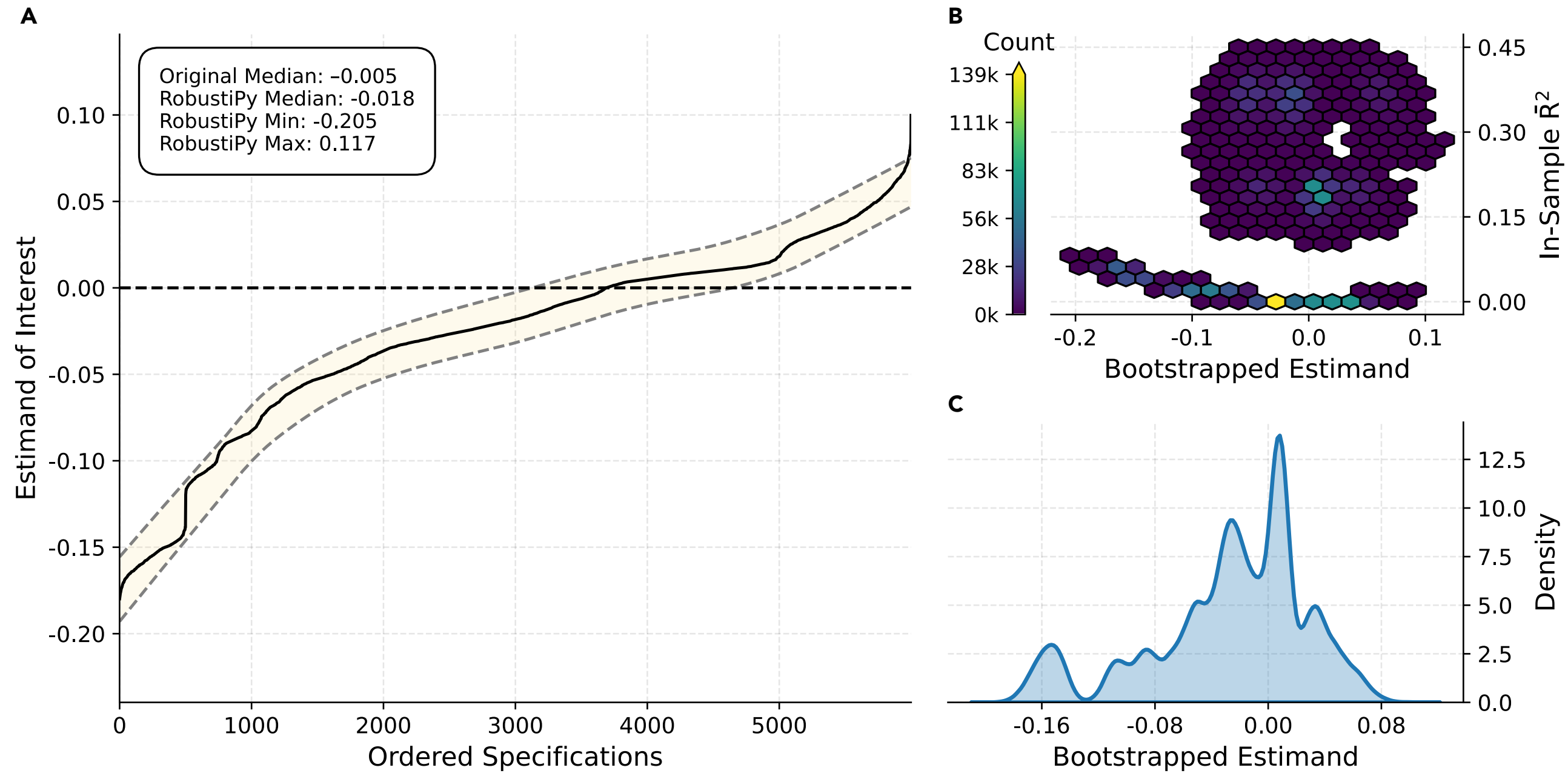

Figure 8. Example output utilizing multiple dependent variables

This example involves multiple dependent variables and rescaled independent variables. It also involves a range of different predictor sets and multiple runs of RobustiPy stacked together using .concat_results. It largely substantiates the results of the original study, showing that the estimand of interest straddles both above and below zero. This example is run with 250 combinations of multiple dependents and 250 bootstrap resamples due to computational cost, showing the flexibility of RobustiPy to adapt to computational power.

tools, tests, and features as possible while attempting to maintain the simplest possible interface in order to encourage researchers to explore the robustness of the models that test their research hypotheses. Through the analysis of multiple aggregated runs of RobustiPy, researchers can also make assertions about whether to include transformed variables, such as Z^2 instead of Z.

Importantly, our application is highly modular and easily extendible. We envision tools such as RobustiPy as a necessity for a large majority of empirical workflows, but there are some natural limitations that warrant further elaboration. First, while RobustiPy offers a comprehensive array of tools to conduct various forms of model uncertainty, many aspects of the process rely heavily on decisions made by the researcher. The selection of a defensible array of control variables, the choice of the underlying estimator, and, ultimately, the hypothesis to be tested are all left to the researcher's discretion; spurious or poorly specified models will still yield spurious and poorly specified results, even when implemented using tools like RobustiPy (despite the fact that we also weight by information criteria, as advocated in the literature[12]). Our model-averaged estimates should be interpreted as conditional on the assumptions embedded in the candidate models and in the information criterion weighting scheme. AIC/BIC weights are derived from an assumed likelihood, large-sample approximations and a specific objective that balances relative fit against complexity; they are not designed to prioritize unbiasedness. Consequently, selecting or averaging "preferred" estimates can still transmit misspecification, and it does not address omitted variable bias, which is a separate threat arising from excluded covariates or incorrect functional form. For this reason, the weighted estimate is best viewed as a pragmatic summary of model uncertainty rather than a claim of bias-free inference. Moreover, information criteria reward predictive adequacy under the assumed model class, not causal identification or robustness to unmeasured confounding, so a higher weight does not guarantee greater substantive validity. This implies that differences across specifications may reflect both genuine sensitivity and the limits of the model class itself and that small shifts in covariate choice or functional form can meaningfully change weighted summaries. The central interpretive point is that model averaging can stabilize estimates when evidence is diffuse, but it cannot substitute for theory-driven model construction or for designs that directly mitigate bias. These limits should temper over-interpretation of precise point estimates and encourage emphasis on uncertainty and sensitivity. Our application does not presently conduct mis-specification tests, for example, which is a natural extension. In particular, formal diagnostics for functional form, heteroskedasticity, serial correlation, and omitted-variable bias remain external to the current implementation and must be undertaken separately by the researcher.

Weighting only has meaning within the space of models to which it is applied, so the central practical question is whether the candidate set spans a reasonable and defensible specification space. Any weighting rule will reflect, and potentially amplify, the structure built into that space, including decisions about covariates, functional forms, identification strategies, and preprocessing. In this sense, there is no universally superior weighting procedure; the most appropriate choice depends on the available inputs and the inferential goal, but all of them inherit the limits of the model space over which they operate.[14] Relatedly, fit-based selection can privilege models that align poorly with

Table 2. Comparative analysis of different datasets with various specifications and results

	All specs, with resamples			All specs, no resamples				Stouffer's		OOS pseudo-R^{2}[b]		
Replication	Min	Median	Max	Min	Median	Max	BIC[a]	Z	p Value	Min	Max	Notes
Union dataset	−1.2	13.5	31.7	8.10	13.5	23.7	9.8	5.5	0.001	0.0	0.3	see Young and Holsteen[41]
Acemoglu et al.[48]	0.23	0.44	0.62	0.39	0.44	0.53	0.42	25.4	0.001	0.59	0.7	constant in $\overleftrightarrow{\mathbf{X}}$
Acemoglu et al.[48]	0.23	0.44	0.62	0.39	0.81	1.23	0.42	25.4	0.001	−0.43	0.7	constant in $\overleftrightarrow{\mathbf{Z}}$
Vandaele[49]	−0.87	0.61	2.03	−0.23	0.70	1.09	0.73	3.68	0.001	−0.51	0.49	see also Ehrlich[57]
Mankiw et al.[50]	−0.55	0.46	2.24	0.26	0.41	1.31	0.30	2.90	0.021	0.31	0.8	$\overleftrightarrow{\mathbf{X}} = \left[\log\frac{I}{Y}, \log(n+g+\delta)\right]$
Mankiw et al.[50]	−3.59	−0.34	1.99	−1.82	−0.21	0.27	−0.17	−0.98	0.350	0.31	0.8	$\overleftrightarrow{\mathbf{X}} = \left[\log(n+g+\delta), \log\frac{I}{Y}\right]$
Understanding society	0.01	0.01	0.01	0.01	0.01	0.01	0.01	37.1	0.001	0.01	0.01	grouping variable
Zhang et al.[51]	−0.46	0.21	0.85	0.17	0.20	0.24	0.18	1.33	0.330	0.0001	0.009	multiple $\overleftrightarrow{\mathbf{X}}$
Dawber et al.[52]	−0.06	0.03	0.10	0.0	0.02	0.06	0.01	n/a	n/a	0.01	0.1	LRobust
Dawber et al.[52]	−0.01	0.00	0.01	0.0	0.00	0.01	0.00	2.06	0.064	0.01	0.09	OLSRobust
Passenger satisfaction	0.01	0.01	0.02	0.01	0.01	0.02	0.01	n/a	n/a	0.02	0.23	LRobust
Passenger satisfaction	0.00	0.00	0.00	0.00	0.00	0.00	0.00	38.2	0.001	0.02	0.22	OLSRobust
Gino et al.[62]	−0.62	−0.20	0.21	−0.24	−0.20	−0.14	n/a	−50.05	0.001	0.02	0.07	promotion, published
Gino et al.[62]	−0.16	0.33	0.84	0.20	0.33	0.42	n/a	80.12	0.001	0.02	0.07	prevention, published
Gino et al.[62]	−0.44	0.04	0.54	−0.01	0.03	0.09	n/a	8.71	0.001	0.0	0.02	promotion, reconstructed
Gino et al.[62]	−0.62	−0.20	0.25	−0.27	−0.20	−0.14	n/a	−47.32	0.001	0.0	0.02	prevention, reconstructed
Gino et al.[62]	−0.19	0.21	0.64	0.20	0.21	0.24	n/a	18.05	0.001	0.04	0.06	promotion, published[c]
Gino et al.[62]	−0.81	−0.37	0.06	−0.41	−0.37	−0.28	n/a	−29.60	0.001	0.04	0.06	prevention, published[c]
Gino et al.[62]	−0.44	−0.05	0.34	−0.07	−0.06	−0.03	n/a	−4.32	0.001	−0.01	0.01	promotion, reconstructed[c]
Gino et al.[62]	−0.54	−0.09	0.37	−0.14	−0.09	−0.01	n/a	−7.26	0.001	−0.01	0.01	prevention, reconstructed[c]
Orben and Przybylski[54]	−0.20	−0.02	0.12	−0.18	−0.02	0.1	n/a	−531.29	0.004	0.00	0.42	multiple dependents

All results were obtained using RobustiPy. RobustiPy offers various alternate out-of-sample model evaluation metrics. Please see our data availability statement for information on accessing each of these datasets. Information criterion-weighted estimands are omitted for studies with multiple dependent variables; the log likelihood is defined with respect to the probability density or mass function of a specific dependent variable, and different outcome composites induce different likelihood functions, rendering BIC-implied weights incommensurable across the full multiple-Y specification space. It is for this reason that our visualizations for this type of analysis omit panels that rely on likelihood-comparable metrics. The results for Orben and Przybylski[54] are created from 250 bootstrapped draws; likelihood-based weighted summaries are omitted for this multiple-dependent-variable analysis because the outcome scale varies across specifications.

[a]Estimands weighted by the Bayesian information criterion (RobustiPy also calculates the AIC and HQIC, with ready extension to other information criteria).

[b]The out-of-sample pseudo-R^2 metric used in the corresponding replication notebooks.

[c]Use of networking intention items.

theoretical expectations, underscoring the need to justify the specification space itself.[65] However, ideological definitions drive what is constituted as a "reasonable" space, and that itself is contested.[66] The key implication is that careful construction and transparent reporting of a defensible model space is at least as important as the choice of weighting rule.

While analysis of this kind provides a computationally powerful framework for testing the robustness of statistical models, readers should not assume that it is the ultimate or only approach for such assessments. Like any method, it is most useful when applied critically and in conjunction with substantive theoretical reasoning. The natural extensions to RobustiPy are limitless. We plan to explore incorporating Laplace approximations by expanding each model's log posterior around its maximum *a posteriori* estimate, using the resulting Gaussian integral as an efficient proxy for the marginal likelihood, thereby enabling scalable comparisons across large model spaces[67] in order to reduce compute costs.[68] Other natural extensions involve the incorporation of additional estimators beyond the OLS, Logit, and fixed-effects models currently supported.

METHODS

Metrics, methods, and tests

In-sample metrics

RobustiPy provides an array of in-sample metrics to evaluate the robustness and quality of analysis. These metrics are calculated

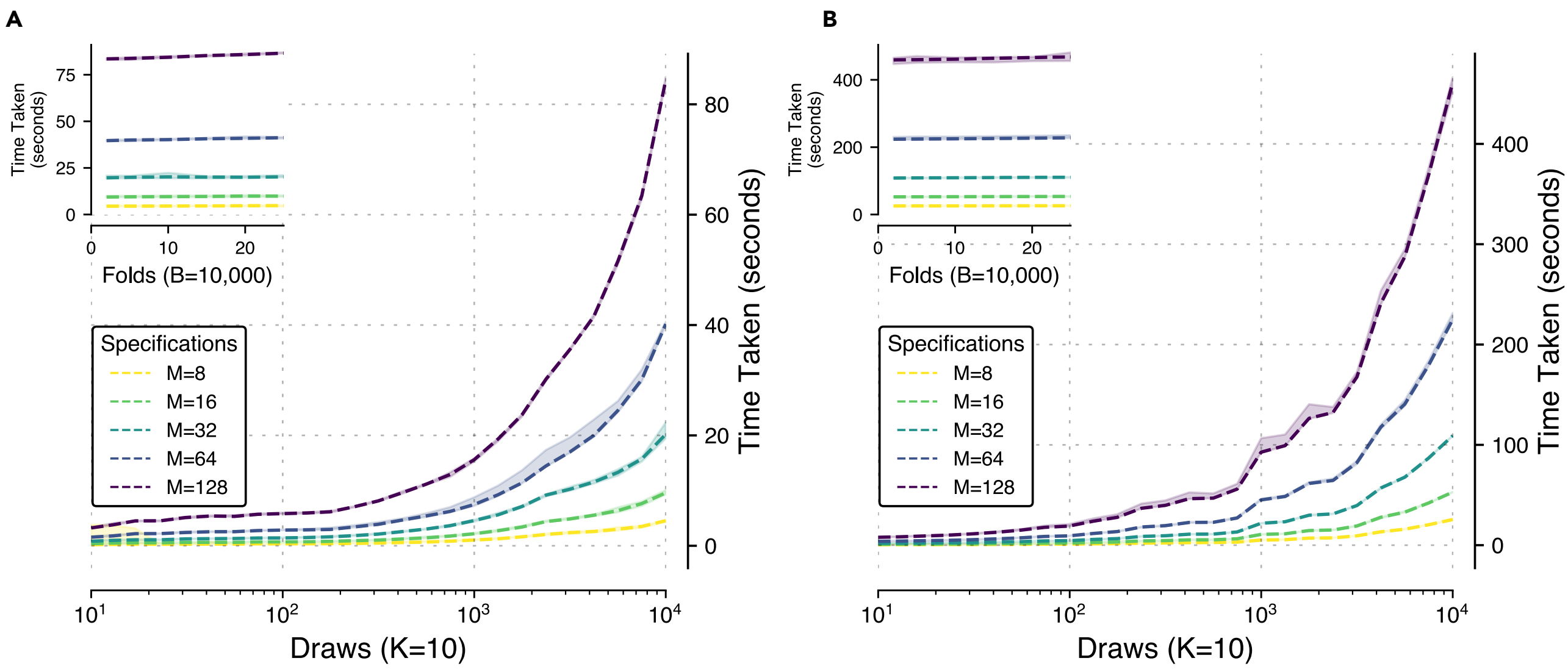

Figure 9. Time profiling of RobustiPy with simulated data
(A) The OLSRobust class.
(B) The LRobust class.
Shaded areas show the range across 10 trials, defined as the interval between the minimum and maximum runtime for a fixed parameterization. Dashed lines show the median. Insets show variation across the number of folds (k), whereas the main subfigures show variation across the number of draws (b).

for all models and samples and stored in the results class to maximize their availability to the end user.

Coefficient of determination and log likelihoods

For each ordinary least squares specification, RobustiPy computes the adjusted in-sample coefficient of determination,

$$\bar{R}^2_\pi = 1 - \frac{\text{RSS}_\pi/(N_\pi - q_\pi)}{\text{TSS}_\pi/(N_\pi - 1)},\quad \text{RSS}_\pi = \sum_{i=1}^{N_\pi}(y_i - \hat{y}_i)^2,\quad \text{TSS}_\pi = \sum_{i=1}^{N_\pi}(y_i - \bar{y}_\pi)^2. \qquad \text{(Equation 22)}$$

Here, N_π is the number of observations used by specification π; q_π is the number of estimated regression coefficients in that specification, including the intercept when present; y_i are observed values; $\hat{y}_i$ are fitted values; and $\bar{y}_\pi$ is the mean of the observed outcome in that estimation sample. RobustiPy also prints summary information about the calculations it undertakes, including the maximum value of $\bar{R}^2$ and the specification $\pi^*_{\bar{R}^2}$ to which it belongs, as follows:

$$\pi^*_{\bar{R}^2} = \arg\max_{\pi\in\Pi} \bar{R}^2_\pi, \qquad \text{(Equation 23)}$$

where Π is the set of models in the choice space, and $\bar{R}^2_\pi$ is the adjusted R^2 value of the π-th model. In the case of logistic regressions, we calculate

$$R^2_{\text{McF}} = 1 - \frac{\sum_{i=1}^{N}[y_i \ln \hat{y}_i + (1 - y_i)\ln(1 - \hat{y}_i)]}{\sum_{i=1}^{N}[y_i \ln \bar{y} + (1 - y_i)\ln(1 - \bar{y})]}, \qquad \text{(Equation 24)}$$

which is 1 minus the ratio of the full-model to null-model log likelihood, where $\hat{y}_i$ is the model's predicted probability that observation i has an outcome equal to 1. RobustiPy also calculates the specification that maximizes this metric in the logistic case, equivalent to Equation 23. In the case of the LRobust class, the full model log likelihood is also returned for each specification.

For OLSRobust, we compute the Gaussian full-model log likelihood for each specification (noting that the π term in $\log(2\pi)$ refers to the mathematical constant, not the specification notation):

$$\log L^{\text{full}}_\pi = -\frac{N_\pi}{2}(1 + \log(2\pi)) - \frac{N_\pi}{2}\log\left(\hat{\sigma}^2_{\pi,\text{full}}\right),\ \hat{\sigma}^2_{\pi,\text{full}} = \frac{1}{N_\pi}\sum_{i=1}^{N_\pi}(y_i - \hat{y}_i)^2. \qquad \text{(Equation 25)}$$

We additionally compute an intercept-only null-model log likelihood on the same sample:

$$\log L^{\text{null}}_\pi = -\frac{N_\pi}{2}(1 + \log(2\pi)) - \frac{N_\pi}{2}\log\left(\hat{\sigma}^2_{\pi,\text{null}}\right), \qquad \text{(Equation 26)}$$

where

$$\hat{\sigma}^2_{\pi,\text{null}} = \frac{1}{N_\pi}\sum_{i=1}^{N_\pi}(y_i - \bar{y})^2. \qquad \text{(Equation 27)}$$

For OLS ranking and visualization, we report the null-relative gain in log likelihood per observation:

$$\Delta\log L^{(\text{per obs})}_\pi = \frac{\log L^{\text{full}}_\pi - \log L^{\text{null}}_\pi}{N_\pi}. \qquad \text{(Equation 28)}$$

This metric is invariant to linear rescaling of the dependent variable ($y \mapsto cy$, for $c > 0$). The specification $\pi^*_{\Delta\log L}$, which maximizes this metric, is presented in the summary method, determined as follows:

$$\pi^*_{\Delta \log L} = \arg\max_{\pi \in \Pi} \Delta \log L_\pi^{(\text{per obs})}, \quad \text{(Equation 29)}$$

where $|\Pi|$ is the total number of specifications in the model space. Raw full-model log likelihoods are also stored and printed in the summary output.

Information criteria

RobustiPy also computes three information criteria for each specification: AIC,[69] BIC,[70] and HQIC.[71] For specification π, each information criterion is calculated from the model's maximized full log likelihood:

$$\text{AIC}_\pi = 2q_\pi - 2 \log L_\pi^{\text{full}}, \quad \text{(Equation 30)}$$

$$\text{BIC}_\pi = q_\pi \log N_\pi - 2 \log L_\pi^{\text{full}}, \quad \text{(Equation 31)}$$

$$\text{HQIC}_\pi = 2q_\pi \log(\log N_\pi) - 2 \log L_\pi^{\text{full}}. \quad \text{(Equation 32)}$$

Here, q_π is the number of penalized free parameters under the convention used by the implementation, N_π is the number of observations, and $\log L_\pi^{\text{full}}$ is the maximized full-model log likelihood. Information criteria are comparable only among specifications fitted to the same response variable, likelihood family, and estimation sample. When Π contains different outcome operationalizations or composite outcomes, AIC/BIC/HQIC should be interpreted as within-outcome fit summaries rather than as weights over a single common likelihood. As before, all information criteria are saved in the results class, and the minimum value and its associated model are provided by the summary method:

$$\pi^*_{\text{IC}} = \arg\min_{\pi \in \Pi_0} \text{IC}_\pi, \quad \text{(Equation 33)}$$

Here, $\Pi_0 \subseteq \Pi$ denotes a likelihood-comparable set of specifications sharing the same response variable, likelihood family, and estimation sample. where IC$\in${AIC,BIC,HQIC}. As a rule of thumb, smaller values indicate a better fit/parameterization trade-off within a likelihood-comparable model set.

Out-of-sample metrics

In addition to in-sample metrics, RobustiPy provides multiple k-fold cross-validated out-of-sample metrics for each estimated model. Given a dataset $\mathcal{D}$, defined multiple times by different sets of predictors $\overleftrightarrow{\mathbf{X}}^{(\pi)}$, where π indexes different model specifications, we perform analysis with k-fold cross-validation to evaluate a predictive model. Each specification and associated dataset $\mathcal{D}^{(\pi)}$ consists of

$$\mathcal{D}^{(\pi)} := \left\{ \left(\overleftrightarrow{\mathbf{X}}_i^{(\pi)}, \overleftrightarrow{\mathbf{Z}}_i^{(\pi)}, \overleftrightarrow{\mathbf{Y}}_i^{(\pi)} \right) \right\}_{i=1}^{N^{(\pi)}}, \quad \text{(Equation 34)}$$

where $N^{(\pi)}$ is the sample size specific to specification π after all preprocessing, X_π is the selected fixed-predictor vector, Z_π is the selected control subset, and Y_π is the selected outcome or outcome composite. For each specification π, we divide $\mathcal{D}^{(\pi)}$ into K approximately equal-sized, mutually exclusive subsets $\{\mathcal{D}_1^{(\pi)}, \mathcal{D}_2^{(\pi)}, \ldots, \mathcal{D}_K^{(\pi)}\}$ so that

$$\mathcal{D}_k^{(\pi)} \cap \mathcal{D}_j^{(\pi)} = \emptyset \text{ for } k \neq j, \bigcup_{k=1}^{K} \mathcal{D}_k^{(\pi)} = \mathcal{D}^{(\pi)}. \quad \text{(Equation 35)}$$

For ungrouped LRobust, folds are generated with stratified splitting to preserve class balance. When group is provided (for either OLSRobust or LRobust), folds are group disjoint (GroupKFold), requiring K to be no larger than the number of unique groups. For ungrouped LRobust, K must also not exceed the minority-class count after preprocessing. For each fold $k\in\{1, \ldots, K\}$ and specification π, define a training dataset $\mathcal{D}_{\text{train}}^{(k,\pi)} = \mathcal{D}^{(\pi)} \backslash \mathcal{D}_k^{(\pi)}$ and a validation set, $\mathcal{D}_{\text{val}}^{(k,\pi)} = \mathcal{D}_k^{(\pi)}$. Train the model on $\mathcal{D}_{\text{train}}^{(k,\pi)}$ to obtain the learned parameters $\theta^{(k,\pi)}$ and then evaluate it on $\mathcal{D}_{\text{val}}^{(k,\pi)}$. Users then specify or are prompted to select their preferred out-of-sample metric (oos_metric) of choice into the .fit() method. For both OLSRobust and LRobust, we calculate the root-mean-square error (selected by choosing oos_metric = 'rmse') for fold k and specification π, given by

$$\text{RMSE}_k^{(\pi)} = \sqrt{\frac{1}{\left|\mathcal{D}_k^{(\pi)}\right|} \sum_{i \in \mathcal{D}_k^{(\pi)}} \left(y_i - f\left(\overleftrightarrow{\mathbf{X}}_i^{(\pi)}, \overleftrightarrow{\mathbf{Z}}_i^{(\pi)}; \theta^{(k,\pi)} \right) \right)^2}. \quad \text{(Equation 36)}$$

We also allow the users to evaluate their out-of-sample model performance with the pseudo-R^2 metric,[72] selected through oos_metric = 'pseudo-r2'. This is otherwise known as the Brier skill score when evaluating probabilities, as in the case of LRobust.[73] For fold k and specification π,

$$\text{Pseudo-R}_k^{2(\pi)} = 1 - \frac{\sum_{i \in \mathcal{D}_k^{(\pi)}} \left(y_i - f_\pi\left(X_{i,\pi}, Z_{i,\pi}; \theta^{(k,\pi)} \right) \right)^2}{\sum_{i \in \mathcal{D}_k^{(\pi)}} \left(y_i - \overline{y}_{\text{train}}^{(k,\pi)} \right)^2}, \quad \text{(Equation 37)}$$

where $\overline{y}_{\text{train}}^{(k,\pi)}$ is the mean of y_i in the training set for fold k and specification π. In the case of LRobust, when calculating an out-of-sample R^2, we also allow the user to choose to evaluate their model with McFadden's-R^2 metric (oos_metric = 'mcfaddens-r2') but instead use the mean of the training dataset in the calculation of the denominator of Equation 24. We additionally include the cross entropy as an out-of-sample metric for evaluating probabilistic predictions in binary classification models (oos_metric = 'cross-entropy'). This metric penalizes confident but incorrect predictions more heavily, making it particularly useful for assessing well-calibrated models. For fold k and specification π, the empirical cross entropy is defined as

$$\text{Empirical Cross Entropy}_k^{(\pi)} = -\frac{1}{\left|\mathcal{D}_k^{(\pi)}\right|} \sum_{i \in \mathcal{D}_k^{(\pi)}} \left[y_i \log\left(f\left(\overleftrightarrow{\mathbf{X}}_i^{(\pi)}, \overleftrightarrow{\mathbf{Z}}_i^{(\pi)}; \theta^{(k,\pi)} \right) \right) + (1 - y_i) \log\left(1 - f\left(\overleftrightarrow{\mathbf{X}}_i^{(\pi)}, \overleftrightarrow{\mathbf{Z}}_i^{(\pi)}; \theta^{(k,\pi)} \right) \right) \right]. \quad \text{(Equation 38)}$$

In addition, we include the IMV[74–76] for binary classifications (oos_metric = 'imv'). For fold k and specification π, it is defined as

$$\text{IMV}_k^{(\pi)} = \frac{w_{\text{enhanced}}^{(k,\pi)} - w_{\text{null}}^{(k,\pi)}}{w_{\text{null}}^{(k,\pi)}}. \quad \text{(Equation 39)}$$

For a vector of validation-fold predicted probabilities $\widehat{\mathbf{p}} = (\widehat{p}_i)_{i \in \mathcal{D}_k^{(\pi)}}$, define the validation-fold mean log score

$$\ell(\widehat{\mathbf{p}}) = \frac{1}{\left|\mathcal{D}_k^{(\pi)}\right|} \sum_{i \in \mathcal{D}_k^{(\pi)}} [y_i \log \widehat{p}_i + (1 - y_i)\log(1 - \widehat{p}_i)], \quad \text{(Equation 40)}$$

with probabilities clipped away from 0 and 1 in implementation. Define

$$h(w) = w \log w + (1 - w)\log(1 - w), w \in [1/2, 1). \quad \text{(Equation 41)}$$

The entropic representation of a predictive model is

$$w(\widehat{\mathbf{p}}) = \arg \min_{w \in [1/2,1)} \{h(w) - \ell(\widehat{\mathbf{p}})\}^2. \quad \text{(Equation 42)}$$

The enhanced value $w_{\text{enhanced}}^{(k,\pi)}$ uses the fitted model predictions on the validation fold. The null value $w_{\text{null}}^{(k,\pi)}$ uses the training-fold prevalence $\bar{y}_{\text{train}}^{(k,\pi)}$ as the constant prediction for all validation observations. These metrics are then averaged over the corresponding K folds accordingly, as follows: $\text{RMSE}_{\text{CV},\pi} = \frac{1}{K}\sum_{k=1}^{K} \text{RMSE}_{k,\pi}$, $\text{Pseudo-R}^2_{\text{CV},\pi} = \frac{1}{K}\sum_{k=1}^{K} \text{Pseudo-R}^2_{k,\pi}$, $\text{McFadden's-R}^2_{\text{CV},\pi} = \frac{1}{K}\sum_{k=1}^{K} \text{McFadden's-R}^2_{k,\pi}$, $\text{Cross Entropy}_{\text{CV},\pi} = \frac{1}{K}\sum_{k=1}^{K} \text{Cross Entropy}_{k,\pi}$, and $\text{IMV}_{\text{CV},\pi} = \frac{1}{K}\sum_{k=1}^{K} \text{IMV}_{k,\pi}$. To compare multiple specifications, we compute, store, and make readily available the metrics for each π and analyze their distributions across arrays stored as follows:

$$\mathcal{S}_{\text{RMSE}} := \left[\text{RMSE}_{\text{CV},\pi}\right]_{\pi=1}^{|\Pi|}, \quad \text{(Equation 43)}$$

$$\mathcal{S}_{\text{Pseudo-R}^2} := \left[\text{Pseudo-R}^2_{\text{CV},\pi}\right]_{\pi=1}^{|\Pi|}, \quad \text{(Equation 44)}$$

$$\mathcal{S}_{\text{McFadden's-R}^2} := \left[\text{McFadden's-R}^2_{\text{CV},\pi}\right]_{\pi=1}^{|\Pi|}, \quad \text{(Equation 45)}$$

$$\mathcal{S}_{\text{Cross Entropy}} := \left[\text{Cross Entropy}_{\text{CV},\pi}\right]_{\pi=1}^{|\Pi|}, \quad \text{(Equation 46)}$$

$$\mathcal{S}_{\text{IMV}} := \left[\text{IMV}_{\text{CV},\pi}\right]_{\pi=1}^{|\Pi|}, \quad \text{(Equation 47)}$$

where $|\Pi|$ is the total number of models estimated.

Computational abilities

RobustiPy performs an array of computations for analyzing each model in the choice space ($\pi \in \Pi$).

Bootstrapping

To quantify uncertainty in the regression estimates in the specification/choice curve, RobustiPy performs a bootstrap procedure to estimate the confidence interval of the effect between $\overleftrightarrow{\mathbf{Y}}$ and the key estimand of interest, $\mathbf{X}_1$. For each choice $\pi \in \Pi$, do the following:

1. Fit the model to observed data. Let $\mathcal{A}_\pi$ denote the estimation routine implied by specification π, and let $t(\cdot)$ extract the focal coefficient. Then,(Equation 48)

$$\widehat{f}_\pi = \mathcal{A}_\pi(\mathcal{D}^{(\pi)}), \widehat{\omega}_\pi = t(\widehat{f}_\pi), \quad \text{(Equation 48)}$$

   where $\widehat{\omega}_\pi$ is typically the coefficient of the predictor of interest $\mathbf{X}_{1,\pi}$.
2. Draw B bootstrap replicates b from $\mathcal{D}^{(\pi)}$. For ungrouped models, rows are resampled with replacement; when group is provided, groups are resampled with replacement (cluster bootstrap) until the requested row target (sample_size, defaulting to full sample size) is reached, so realized replicate size may vary.
3. Re-estimate the model in each replicate b:

$$\widehat{f}_\pi^{(b)} = \mathcal{A}_\pi\left(\mathcal{D}_\pi^{(b)}\right), \widehat{\omega}_\pi^{(b)} = t\left(\widehat{f}_\pi^{(b)}\right), b = 1, \ldots, B. \quad \text{(Equation 49)}$$

4. Construct the empirical distribution of coefficients $\widehat{\omega}_\pi^{(b)}$ across all bootstrap replicates

$$\mathcal{S}_\pi^{\text{boot}} := \left\{\widehat{\omega}_\pi^{(b)}\right\}_{b=1}^{B}. \quad \text{(Equation 50)}$$

Let $c \in (0,1]$ denote the user-specified confidence level (ci = c, default c = 1). The corresponding confidence interval is

$$\text{CI}_\omega = \left\{\left[Q_{(1-c)/2}\left(\mathcal{S}_\pi^{\text{boot}}\right), Q_{1-(1-c)/2}\left(\mathcal{S}_\pi^{\text{boot}}\right)\right] : \pi \in \Pi\right\}. \quad \text{(Equation 51)}$$

where Q_α denotes the empirical quantile function. Values (for the specification curve subfigure) can be optionally LOESS smoothed for better visualization, but raw estimates are kept. If singleton filtering yields unstable grouped samples, implementation may fall back to unfiltered grouped draws to avoid hard failure.

Bayesian model averaging for control coefficients (BIC-implied weights)

RobustiPy performs Bayesian model averaging (BMA) for variables specified in the candidate control space. The focal predictor(s) in $\overleftrightarrow{\mathbf{X}}_\pi$ are included in every specification, so they are not averaged over. BMA weights are computed only within a likelihood-comparable comparison set $\Pi_0 \subseteq \Pi$, typically a set of specifications sharing the same outcome operationalization, likelihood family, and estimation sample. For a linear specification $\pi \in \Pi_0$, write

$$\overleftrightarrow{\mathbf{Y}}_\pi = \left[\overleftrightarrow{\mathbf{X}}_\pi, \overleftrightarrow{\mathbf{Z}}_\pi\right]\boldsymbol{\beta}_\pi + \epsilon_\pi, \epsilon_\pi \sim \mathcal{N}\left(0, \sigma_\pi^2 \mathbf{I}_{N_\pi}\right). \quad \text{(Equation 52)}$$

The BIC-implied model weight is

$$w_\pi := \frac{\exp\left[-\frac{1}{2}\text{BIC}_\pi\right]}{\sum_{\pi' \in \Pi_0} \exp\left[-\frac{1}{2}\text{BIC}_{\pi'}\right]},$$

$$\sum_{\pi \in \Pi_0} w_\pi = 1, \quad \text{(Equation 53)}$$

where

$$\text{BIC}_\pi = q_\pi \log N_\pi - 2 \log \widehat{L}_\pi. \quad \text{(Equation 54)}$$

These weights are a BIC approximation to posterior model probabilities under equal prior model probabilities and regularity assumptions; they should not be interpreted as exact posterior probabilities outside those assumptions. For $p \in \{1, \ldots, d_Z\}$, let Z_p denote the p-th candidate control and define its inclusion indicator in specification π by

$$I_{p,\pi} := \mathbf{1}\{Z_p \in Z_\pi\}. \quad \text{(Equation 55)}$$

Let $\widehat{\omega}_{p,\pi}$ be the estimated coefficient of control p when $I_{p,\pi} = 1$. For unconditional BMA, define the zero-padded coefficient

$$\tilde{\omega}_{p,\pi} := \begin{cases} \widehat{\omega}_{p,\pi}, & I_{p,\pi} = 1, \\ 0, & I_{p,\pi} = 0. \end{cases} \quad \text{(Equation 56)}$$

The unconditional BMA estimate is then

$$\widehat{\omega}_p^{\mathrm{BMA}} = \sum_{\pi \in \Pi_0} w_\pi \tilde{\omega}_{p,\pi}. \quad \text{(Equation 57)}$$

The posterior inclusion probability is

$$\mathrm{PIP}_p = \sum_{\pi \in \Pi_0} w_\pi I_{p,\pi}. \quad \text{(Equation 58)}$$

An effect conditional on inclusion can also be defined as

$$\widehat{\omega}_{p|p \in M}^{\mathrm{BMA}} = \frac{\sum_{\pi \in \Pi_0} w_\pi I_{p,\pi} \widehat{\omega}_{p,\pi}}{\sum_{\pi \in \Pi_0} w_\pi I_{p,\pi}}, \quad \text{(Equation 59)}$$

whenever $\mathrm{PIP}_p > 0$. Repeating this step for $p = 1, \ldots, d_Z$ yields the vector of BMA summaries for candidate controls. The returned BMA table reports PIP_p, the unconditional zero-padded BMA coefficient $\widehat{\omega}_p^{\mathrm{BMA}}$, and, when $\mathrm{PIP}_p > 0$, the conditional-on-inclusion coefficient $\widehat{\omega}_{p|p \in M}^{\mathrm{BMA}}$.

SHAP values for a full-specification predictive model

RobustiPy computes SHAP for the single "full" specification containing all predictors and controls. These SHAP values explain the fitted full-specification prediction model used for the SHAP calculation; they are not averaged over the specification space and should not be interpreted as causal marginal effects. In the current implementation, SHAP values are returned for single-outcome full-specification predictive models when compute_shap = True; multiple-outcome OLS result objects do not currently return a SHAP object. After dropping missing values, the data are split into an 80% training set and a 20% held-out test set. For OLSRobust with group, demeaning is applied before fitting; for LRobust, logistic fixed-effects demeaning is not applied, and group is used for grouped cross-validation/bootstrap resampling. Let

$$\mathbf{U}_i^{\mathrm{test}} = \left(U_{1i}^{\mathrm{test}}, \ldots, U_{qi}^{\mathrm{test}}\right)$$

denote the row vector of non-constant test set features used by the SHAP explanation model. In the implementation, any explicit const column is removed before fitting the SHAP model, and the intercept is handled internally by the scikit-learn estimator. Let $(\tilde{\alpha}, \tilde{\boldsymbol{\beta}})$ denote the intercept and slope vector of the auxiliary full-specification prediction model fitted for SHAP on the training split. For OLSRobust, this auxiliary model is a linear regression and its additive score is

$$\tilde{h}_i = \tilde{Y}_i = \tilde{\alpha} + \sum_{p=1}^{q} U_{pi}^{\mathrm{test}} \tilde{\beta}_p. \quad \text{(Equation 60)}$$

For LRobust, this auxiliary model is a penalized logistic regression, and the additive score is the linear predictor

$$\tilde{h}_i = \tilde{\eta}_i = \tilde{\alpha} + \sum_{p=1}^{q} U_{pi}^{\mathrm{test}} \tilde{\beta}_p, \tilde{p}_i = \Lambda(\tilde{\eta}_i), \Lambda(t) = \frac{1}{1 + \exp(-t)}.$$

Thus, for logistic regression, the additive SHAP decomposition is on the log-odds scale of the auxiliary prediction model, not on the probability scale. Let

$$\mu_p = \mathbb{E}_{\mathrm{train}}[U_p], \quad \text{(Equation 61)}$$

and define the baseline additive score

$$v_{\tilde{h}}(\varnothing) = \tilde{\alpha} + \sum_{p=1}^{q} \tilde{\beta}_p \mu_p. \quad \text{(Equation 62)}$$

Under linear additivity, the SHAP values for the auxiliary additive score are

$$\tilde{\phi}_{p,i} = \tilde{\beta}_p \left(U_{pi}^{\mathrm{test}} - \mu_p\right), \quad \text{(Equation 63)}$$

for $p = 1, \ldots, q$ and $i = 1, \ldots, N_{\mathrm{test}}$, satisfying

$$\tilde{h}_i = v_{\tilde{h}}(\varnothing) + \sum_{p=1}^{q} \tilde{\phi}_{p,i}. \quad \text{(Equation 64)}$$

Rather than aggregating them internally, RobustiPy returns

$$\mathrm{shap\_return} = \left(\left[\tilde{\phi}_{p,i}\right]_{i=1,\ldots,N_{\mathrm{test}};\, p=1,\ldots,q}, \mathbf{U}^{\mathrm{test}}\right). \quad \text{(Equation 65)}$$

These SHAP values explain the auxiliary full-specification prediction model trained for feature explanation. They should not be interpreted as the stored coefficient estimates from every specification in the multiverse nor as causal marginal effects. Users may then compute summaries, such as mean absolute SHAP per feature, or visualize these values as desired.

Specification curve inference

Beyond per-specification uncertainty, `RobustiPy` implements curve-level summaries for the null hypothesis

$$H_0 : \beta_{1,\pi} = 0 \text{ for every } \pi \in \Pi$$

$$H_1 : \beta_{1,\pi} \neq 0 \text{ for at least one } \pi \in \Pi$$

asking whether the entire pattern of focal estimates across the specification space Π is compatible with a zero focal coefficient. For additive OLS-type specifications, the procedure can be interpreted as a bootstrap-calibrated curve-level test. For binary response or other non-additive specifications, the same summaries should be interpreted as descriptive diagnostics unless a model-specific null simulation is supplied.

1. Full-sample fits: for every specification $\pi \in \Pi$, fit on its observed data $\mathcal{D}^{(\pi)}$, storing the coefficient and p value $(\widehat{\omega}_\pi, p_\pi)$.

2. Outcome bootstrap (sampling variability): draw B independent and identically distributed resamples of analysis units. In the single-outcome OLS implementation, the same bootstrap seeds are shared across control specification variants, which preserves the induced dependence structure across the specification curve. If no grouping variable is supplied, units are row indices. If a grouping variable is supplied, units are unique group identifiers, and RobustiPy performs cluster bootstrap resampling with replacement, retaining all rows from sampled groups including multiplicity. Re-estimate $\widehat{\omega}_{\pi}^{(b)}$ for each $\pi \in \Pi$ and $b = 1, \ldots, B$. When different outcome composites induce different estimation samples, the resulting curve-level summaries should be interpreted as descriptive diagnostics unless a common resampling scheme is explicitly imposed.
3. Null bootstrap ($y^{\star}$ construction): for additive OLS-type specifications, construct a null pseudo-outcome by subtracting the fitted contribution of the focal predictor:

$$Y_{\pi}^{\star} = Y_{\pi} - \mathbf{X}_{1,\pi}\widehat{\omega}_{\pi}. \quad \text{(Equation 66)}$$

Re-fit each specification on $Y_{\pi}^{\star}$ using the same B bootstrap resamples at the same sampling level: row level when ungrouped and group level when grouped. This generates a joint null reference distribution of curve-level functionals while preserving the correlation structure induced by resampling the same analysis units across specifications. For LRobust or any bounded/discrete outcome model, this residualized $Y_{\pi}^{\star}$ is not generally a valid outcome under a sharp binary-response null; in those cases, the resulting null summaries are diagnostic rather than exact hypothesis tests.
4. Curve-level functionals: for a vector $A = \{a_{\pi}\}_{\pi \in \Pi}$ and (optionally) p values $p = \{p_{\pi}\}_{\pi \in \Pi}$, define

$$S_1(A) := \text{median}_{\pi} a_{\pi}, \quad \text{(Equation 67)}$$

$$S_2(A) := \min_{\pi} a_{\pi}, \quad \text{(Equation 68)}$$

$$S_3(A) := \max_{\pi} a_{\pi}, \quad \text{(Equation 69)}$$

$$S_4(A) := \sum_{\pi} 1\{a_{\pi} > 0\}, \quad \text{(Equation 70)}$$

$$S_5(A) := \sum_{\pi} 1\{a_{\pi} < 0\}, \quad \text{(Equation 71)}$$

$$S_6(A,P) := \sum_{\pi} 1\{p_{\pi} < 0.05\}, \quad \text{(Equation 72)}$$

$$S_7(A,P) := \sum_{\pi} 1\{a_{\pi} > 0\}\, 1\{p_{\pi} < 0.05\}, \quad \text{(Equation 73)}$$

$$S_8(A,P) := \sum_{\pi} 1\{a_{\pi} < 0\}\, 1\{p_{\pi} < 0.05\}. \quad \text{(Equation 74)}$$

5. Two-sided null tail areas: for each functional S, let S_{obs} denote the value on the observed coefficients $\widehat{\omega}_{\pi}$ and $\left\{S^{\star(b)}\right\}_{b=1}^{B}$ its values under null bootstraps. The two-sided Monte Carlo tail area is

$$p(S_{\text{obs}}) = 2\min\left\{\frac{1 + \sum_{b=1}^{B} 1\left\{S^{\star(b)} \geq S_{\text{obs}}\right\}}{B+1}, \frac{1 + \sum_{b=1}^{B} 1\left\{S^{\star(b)} \leq S_{\text{obs}}\right\}}{B+1}\right\} \quad \text{(Equation 75)}$$

$$p(S_{\text{obs}}) \leftarrow \min\{1, p(S_{\text{obs}})\}. \quad \text{(Equation 76)}$$

clipped at 1. This gives a two-sided null-calibrated diagnostic for the chosen curve-level functional.
6. Model-class caveat: This residualized null-bootstrap procedure is most defensible for additive OLS-type specifications under ideal independent and identically distributed error assumptions, where the bootstrap can approximate the sampling distribution of sufficiently regular functionals of the estimator. The median, minimum, and maximum summaries are non-linear but comparatively stable curve summaries. The sign-count and significance-count summaries $S_4, \ldots, S_8$, however, are discontinuous indicator functionals, so their bootstrap calibration should be interpreted as a finite-sample diagnostic rather than as an asymptotically exact test. For grouped models, RobustiPy uses cluster-level resampling based on the supplied grouping variable; inference is conditional on that grouping variable correctly capturing the relevant dependence structure. For logit models, the score is heteroskedastic and the outcome is bounded, and the current implementation does not construct a residualized binary-response null distribution; these summaries should therefore be interpreted as descriptive diagnostics unless a model-specific null simulation is supplied. If curve-level summaries are computed using data-dependent weights, such as information criterion weights, those weights must be recomputed within each bootstrap replicate to preserve coherence between the observed statistic and its null reference distribution.
7. Meta-analytic Stouffer summary (null calibrated): Let Π denote the specification set. For each $\pi \in \Pi$, retain the full-sample estimate and p value $(\widehat{\beta}_{\pi}, p_{\pi})$. For additive single-outcome OLS-type specifications with joint inference enabled, the null bootstrap additionally provides draws $\left\{\left(\widehat{\beta}_{\pi}^{\star(b)}, p_{\pi}^{\star(b)}\right)\right\}_{b=1}^{B}$ computed on the pseudo-outcome $Y_{\pi}^{\star}$, using shared resampling seeds across control specification variants. For multiple-outcome OLS, non-additive models, or bounded-outcome specifications, the Stouffer quantity remains useful as a dependence-adjusted descriptive summary unless calibrated by a model-specific common null-resampling scheme.

Clipping and signed Z scores: Define the numerically clipped p values

$$p_{\pi}^{\text{clip}} := \min(\max(p_{\pi}, \varepsilon), 1 - \delta) \quad \text{(Equation 77)}$$

CellPress
OPEN ACCESS

$$\varepsilon := \max\left(10^{-300}, \tau_{\text{mach}}\right) \quad \text{(Equation 78)}$$

$$\delta := 10^{-16}, \quad \text{(Equation 79)}$$

where τ_{mach} denotes the smallest positive normal double-precision number (np.finfo(float).tiny). The observed signed Z scores are

$$z_\pi := \text{sign}(\widehat{\beta}_\pi)\, \Phi^{-1}\left(1 - \frac{p_\pi^{\text{clip}}}{2}\right), \quad \text{(Equation 80)}$$

with Φ^{-1} the standard normal quantile. As a safeguard, any non-finite values (which can occur only at extreme tails) are clipped to the range $[-37, 37]$.
From the null draws define, for each $b = 1, \ldots, B$,

$$z_\pi^{\star(b)} := \text{sign}(\widehat{\beta}_\pi^{\star(b)})\, \Phi^{-1}\left(1 - \frac{\left(p_\pi^{\star(b)}\right)^{\text{clip}}}{2}\right). \quad \text{(Equation 81)}$$

Let $Z_{\text{vec}}^{\star(b)} := \left(z_\pi^{\star(b)}\right)_{\pi \in \Pi}$. Specifications with (near) zero null variability are excluded prior to dependence estimation; i.e., we retain

$$\Pi_{\text{keep}} := \left\{\pi \in \Pi : \widehat{\text{sd}}\left(z_\pi^{\star(1:B)}\right) > 10^{-14}\right\} \quad \text{(Equation 82)}$$

and subsequently work with the restricted vectors $Z_{\text{vec,keep}}^{\star(b)} := \left(z_\pi^{\star(b)}\right)_{\pi \in \Pi_{\text{keep}}}$.
Dependence correction from the null bootstrap. Using the B null vectors, form the empirical correlation matrix

$$\widehat{R} := \text{Corr}\left(Z_{\text{vec,keep}}^{\star(1)}, \ldots, Z_{\text{vec,keep}}^{\star(B)}\right). \quad \text{(Equation 83)}$$

To ensure numerical stability, $\widehat{R}$ is projected onto the cone of positive semidefinite matrices (eigenvalue clipping), symmetrized, forced to have unit diagonal, and ridge regularized:

$$\widehat{\Sigma} := \widehat{R}_{\text{psd}} + 10^{-12} I. \quad \text{(Equation 84)}$$

If $\widehat{\Sigma}$ cannot be constructed (e.g., too few null draws), then the implementation falls back to the independence denominator. Because the same denominator divides the observed and all null combined statistics, the dependence estimate Σ∧ rescales the reported Z but cancels in the calibrated p value; Σ∧ thus affects the magnitude of Z, not the significance decision.
Combined statistic. With nonnegative weights w_π (default $w_\pi \equiv 1$) for $\pi \in \Pi_{\text{keep}}$, define

$$Z_{\text{obs}} := \frac{\sum_{\pi \in \Pi_{\text{keep}}} w_\pi z_\pi}{\sqrt{w^\top \widehat{\Sigma}\, w}}, \quad \text{(Equation 85)}$$

where, under the independence fallback, the denominator is $\sqrt{\sum_{\pi \in \Pi_{\text{keep}}} w_\pi^2}$.
Null calibration. For each null draw b, compute the corresponding combined statistic using the *same* denominator as in Equation 85,

$$Z^{\star(b)} := \frac{\sum_{\pi \in \Pi_{\text{keep}}} w_\pi z_\pi^{\star(b)}}{\sqrt{w^\top \widehat{\Sigma}\, w}} \quad \text{(Equation 86)}$$

for $b = 1, \ldots, B$. The two-sided null-calibrated Monte Carlo tail area is

$$p_{\text{Stouffer}} := \frac{1 + \sum_{b=1}^{B} 1\left\{\left|Z^{\star(b)}\right| \geq \left|Z_{\text{obs}}\right|\right\}}{B + 1}. \quad \text{(Equation 87)}$$

Interpretation. Large positive Z_{obs} indicates broadly concordant evidence for $\beta_1 > 0$ across specifications, large negative Z_{obs} indicates concordant evidence for $\beta_1 < 0$, and values near zero indicate weak or conflicting evidence. Invalid or missing $(\widehat{\beta}_\pi, p_\pi)$ are omitted from Π_{keep}, and dependence is estimated from the null bootstrap when available.

8. Stored outputs: all curve-level summaries S_k, their null tail areas where available, and the (Z, p_{Stouffer}) pair are saved in OLSResult.inference for downstream use in .summary() and in visualization methods.

Sub-sampling large specification spaces

When the number of admissible outcome composites $\Pi_{\mathcal{Y}}$ or covariate sets $\Pi_{\mathcal{Z}}$ is very large, estimating *every* admissible model in

$$\Pi \subseteq \Pi_{\mathcal{Y}} \times \Pi_{\mathcal{F}} \times \Pi_{\mathcal{X}} \times \Pi_{\mathcal{Z}}$$

can be computationally prohibitive. RobustiPy therefore lets the user sample outcome-composite subsets and/or control-subset masks before fitting begins.

1. Determine target sizes. Choose integers $m_Y \leq |\Pi_{\mathcal{Y}}|$ and $m_Z \leq |\Pi_{\mathcal{Z}}|$ and the number of outcome and covariate options to keep (defaults are the full sets).
2. Uniform sub-sampling. Independently sample, *without replacement*,

$$\tilde{\Pi}_{\mathcal{Y}} \sim \text{Unif}(\{A_Y \subseteq \Pi_{\mathcal{Y}} : |A_Y| = m_Y\}), \quad \text{(Equation 88)}$$

$$\tilde{\Pi}_{\mathcal{Z}} \sim \text{Unif}(\{A_Z \subseteq \Pi_{\mathcal{Z}} : |A_Z| = m_Z\}), \quad \text{(Equation 89)}$$

using NumPy's fast random sampler.
3. Construct the reduced model space. The analysis then proceeds on

$$\tilde{\Pi} = \Pi \cap (\tilde{\Pi}_{\mathcal{Y}} \times \Pi_{\mathcal{F}} \times \Pi_{\mathcal{X}} \times \tilde{\Pi}_{\mathcal{Z}}), \quad \text{(Equation 90)}$$

which can cut runtime by orders of magnitude while approximating the behavior of the full model space when the sampled masks cover the relevant parts of that space.

In the current release, pre-fit covariate-space sub-sampling is implemented in OLSRobust via z_specs_sample_size. Outcome-composite sub-sampling is also exposed in OLSRobust.fit(…) through composite_sample. When multiple dependent variables are supplied, a positive value samples that many non-empty outcome subsets before composite construction; otherwise, all non-empty outcome subsets are enumerated. The LRobust class currently estimates the full

covariate-subset space for a single binary outcome. The random seeds are user controllable, making implemented sub-sampling routines reproducible conditional on the software environment, dependency versions, and parallelization settings.

RESOURCE AVAILABILITY

Lead contact

Requests for further information and resources should be directed to and will be fulfilled by the lead contact, Charles Rahal (charles.rahal@demography.ox.ac.uk).

Materials availability

This study did not generate new physical materials.

Data and code availability

The extensive code library that accompanies this work can be found at github.com/RobustiPy on the Python Package Index, which avails an install as simple as a pip install robustipy command and is also indexed into Zenodo.[13] Documentation is available at robustipy.readthedocs.io.

All but three of our empirical examples utilize publicly available information, and those three are in general highly accessible upon requests to the relevant parties. In our replication files,[13] two of these three require a simple and immediately granted application to the UK Data Service; these are empirical_type5_orben.ipynb and empirical_type3_ukhls.ipynb. One, empirical_type3_asc.ipynb, requires a request to the authors.[51] The other seven are entirely publicly available, but we re-share no data as part of our library in order to be maximally respectful of the relevant data licenses (even if some permit re-sharing and are in the public domain). Of these, our replication files automatically scrape necessary data on the fly: empirical_type1_acemoglu.ipynb pulls a publicly available file from the Dropbox of a study's author, empirical_type1_ehrlich.ipynb from an academic's homepage, empirical_type1_union.ipynb from Stata Press, and empirical_type2_mrw.ipynb from dataverse.nl. Two resources come from the Python library kagglehub and are used in empirical_type4_airline.ipynb and empirical_type4_framingham.ipynb. Finally, the notebook empirical_type5_gino.ipynb pulls data from researchbox.org. Kindly let us know if you spot that any of the necessary resources become unavailable in their original locations, and we will update our empirical examples in our library accordingly.

ACKNOWLEDGMENTS

The authors are grateful for comments on earlier versions of the work from participants at the International Conference on Computational Social Science, the British Society for Population Studies, the European Consortium for Sociological Research, the Population Association of America, Tsinghua University, the International Conference on Social Computing, and internal feedback received from the Leverhulme Centre for Demographic Science, Reproducible Research Oxford, the Big Data Institute's Data Engineers Meeting, and the Pandemic Sciences Institute. We also thank all attendees at a previous hackathon, hosted at the University of Oxford in June 2024. We especially thank Anda-Raluca Epure for detailed comments on the manuscript and an extensive code review of the accompanying software. The authors are grateful for funding from the ESRC (grant ES/W002302/1), the Leverhulme Trust (grant RC-2018-003) for the Leverhulme Centre for Demographic Science and Nuffield College, and a Grand Union DTP ESRC studentship.

AUTHOR CONTRIBUTIONS

C.R. initiated the software development of the library. This work was subsequently developed further in collaboration with D.V. and J.Y. Detailed code reviews were undertaken by J.Y., D.V., and D.D. All authors contributed to the creation of the manuscript, participated in regular discussions of the key concepts and derivations, and contributed to the manuscript revisions.

DECLARATION OF INTERESTS

The authors declare no competing interests.

DECLARATION OF GENERATIVE AI AND AI-ASSISTED TECHNOLOGIES IN THE WRITING PROCESS

During the preparation of this work, the authors used ChatGPT and Claude to improve the grammar and flow of the manuscript. After using these tools/services, the authors reviewed and edited the content as needed and take full responsibility for the content of the publication.

SUPPLEMENTAL INFORMATION

Supplemental information can be found online at https://doi.org/10.1016/j.patter.2026.101609.

Received: October 21, 2025
Revised: March 5, 2026
Accepted: June 3, 2026
Published: August 14, 2026

REFERENCES

1. Oreskes, N. (2003). The Role of Quantitative Models in Science. Models in Ecosystem Science *13*, 31–42.
2. Camerer, C.F., Dreber, A., Holzmeister, F., Ho, T.H., Huber, J., Johannesson, M., Kirchler, M., Nave, G., Nosek, B.A., Pfeiffer, T., et al. (2018). Evaluating the Replicability of Social Science Experiments in Nature and Science Between 2010 and 2015. Nat. Hum. Behav. *2*, 637–644.
3. Klein, R.A., Ratliff, K.A., Vianello, M., Adams, R.B., Jr., Bahník, S., Bernstein, M.J., Bocian, K., Brandt, M.J., Brooks, B., Brumbaugh, C.C., et al. (2014). Investigating Variation in Replicability. Soc. Psychol. *45*, 142–152.
4. Leamer, E.E. (1983). Let's Take the Con Out of Econometrics. Am. Econ. Rev. *73*, 31–43.
5. Jeffreys, H. (1939). Theory of Probability, 1st ed. (Oxford University Press).
6. Lindley, D.V. (1957). A statistical paradox. Biometrika *44*, 187–192.
7. Gelman, A., and Loken, E. (2013). The Garden of Forking Paths: Why Multiple Comparisons Can Be a Problem Even When There Is No Fishing Expedition or p-Hacking and the Research Hypothesis Was Posited Ahead of Time (Tech. Rep. Department of Statistics, Columbia University).
8. Lundberg, I., Johnson, R., and Stewart, B.M. (2021). What Is Your Estimand? Defining the Target Quantity Connects Statistical Evidence to Theory. Am. Sociol. Rev. *86*, 532–565.
9. Engzell, P., and Mood, C. (2023). Understanding Patterns and Trends in Income Mobility Through Multiverse Analysis. Am. Sociol. Rev. *88*, 600–626.
10. Yan, J., and Rahal, C. (2025). On the Unknowable Limits to Prediction. Nat. Comput. Sci. *5*, 188–190.
11. Young, C. (2019). The Difference Between Causal Analysis and Predictive Models: Response to 'Comment on Young and Holsteen (2017)'. Socio. Methods Res. *48*, 431–447.
12. Slez, A. (2019). The Difference Between Instability and Uncertainty: Comment on Young and Holsteen (2017). Socio. Methods Res. *48*, 400–430.
13. Valdenegro Ibarra, D., Yan, J., Dai, D., and Rahal, C. (2026). RobustiPy: v1.0.3 for Zenodo. Zenodo. https://doi.org/10.5281/zenodo.15700697.
14. Young, C., and Cumberworth, E. (2025). Multiverse Analysis: Computational Methods for Robust Results. Analytical Methods for Social Research (Cambridge University Press). https://doi.org/10.1017/9781009003391.

15. Head, M.L., Holman, L., Lanfear, R., Kahn, A.T., and Jennions, M.D. (2015). The Extent and Consequences of *p*-Hacking in Science. PLoS Biol. *13*, e1002106.

16. Brodeur, A., Lé, M., Sangnier, M., and Zylberberg, Y. (2016). Star Wars: The Empirics Strike Back. Am. Econ. J. Appl. Econ. *8*, 1–32.

17. Kerr, N.L. (1998). HARKing: Hypothesizing After the Results Are Known. Pers. Soc. Psychol. Rev. *2*, 196–217.

18. Wingen, T., Berkessel, J.B., and Englich, B. (2020). No Replication, No Trust? How Low Replicability Influences Trust in Psychology. Soc. Psychol. Personal. Sci. *11*, 454–463.

19. Van't Veer, A.E., and Giner-Sorolla, R. (2016). Pre-Registration in Social Psychology: A Discussion and Suggested Template. J. Exp. Soc. Psychol. *67*, 2–12.

20. Edlund, J.E., Cuccolo, K., Irgens, M.S., Wagge, J.R., and Zlokovich, M.S. (2022). Saving science through replication studies. Perspect. Psychol. Sci. *17*, 216–225.

21. Benjamin, D.J., Berger, J.O., Johannesson, M., Nosek, B.A., Wagenmakers, E.J., Berk, R., Bollen, K.A., Brembs, B., Brown, L., Camerer, C., et al. (2017). Redefine Statistical Significance. Nat. Hum. Behav. *2*, 6–10.

22. Etz, A., and Vandekerckhove, J. (2016). A Bayesian Perspective on the Reproducibility Project: Psychology. PLoS One *11*, e0149794.

23. Verhagen, M.D. (2024). Incorporating machine learning into sociological model-building. Sociol. Methodol. *54*, 217–268. https://doi.org/10.1177/00811750231217734.

24. Rahal, C., Verhagen, M., and Kirk, D. (2024). The Rise of Machine Learning in the Academic Social Sciences. AI & Society *39*, 799–801. https://doi.org/10.1007/s00146-022-01540-w.

25. Simonsohn, U., Simmons, J.P., and Nelson, L.D. (2020). Specification Curve Analysis. Nat. Hum. Behav. *4*, 1208–1214.

26. Steegen, S., Tuerlinckx, F., Gelman, A., and Vanpaemel, W. (2016). Increasing Transparency Through a Multiverse Analysis. Perspect. Psychol. Sci. *11*, 702–712.

27. Sala-i Martin, X. (1997). I Just Ran Four Million Regressions (National Bureau of Economic Research). https://doi.org/10.3386/w6252.

28. Muñoz, J., and Young, C. (2018). We Ran 9 Billion Regressions: Eliminating False Positives Through Computational Model Robustness. Sociol. Methodol. *48*, 1–33.

29. Hanck, C. (2016). I Just Ran Two Trillion Regressions. Econ. Bull. *36*, 2037–2042.

30. Gelman, A., and Loken, E. (2014). The Statistical Crisis in Science. Am. Sci. *102*, 460–465.

31. Simmons, J.P., Nelson, L.D., and Simonsohn, U. (2011). False-Positive Psychology: Undisclosed Flexibility in Data Collection and Analysis Allows Presenting Anything as Significant. Psychol. Sci. *22*, 1359–1366.

32. Huntington-Klein, N., Arenas, A., Beam, E., Bertoni, M., Bloem, J.R., Burli, P., Chen, N., Grieco, P., Ekpe, G., Pugatch, T., et al. (2021). The Influence of Hidden Researcher Decisions in Applied Microeconomics. Econ. Inq. *59*, 944–960.

33. Ioannidis, J.P.A. (2005). Why Most Published Research Findings Are False. PLoS Med. *2*, e124.

34. Sutton, R. (2019). The Bitter Lesson. Blog post. Incomplete Ideas. https://incompleteideas.net/IncIdeas/BitterLesson.html.

35. Sarma, A., Kale, A., Moon, M.J., Taback, N., Chevalier, F., Hullman, J., and Kay, M. (2023). multiverse: Multiplexing Alternative Data Analyses in R Notebooks. In Proceedings of the 2023 CHI Conference on Human Factors in Computing Systems (Association for Computing Machinery), pp. 148:1–148:15. https://doi.org/10.1145/3544548.3580726.

36. Masur, P.K., and Scharkow, M. (2020). Specr: Conducting and Visualizing Specification Curve Analyses. R Package (CRAN). https://doi.org/10.32614/CRAN.package.specr.

37. Semken, C., and Rossell, D. (2022). Specification analysis for technology use and teenager well-being: Statistical validity and a Bayesian proposal. J. Roy. Stat. Soc. C Appl. Stat. *71*, 1330–1355. https://doi.org/10.1111/rssc.12578.

38. Semken, C., and Rossell, D. (2022). Bayesian Specification Curve Analysis (OSF). https://doi.org/10.17605/OSF.IO/M8D4N.

39. Ortiz-Bobea, A. (2021). Spec_Chart: Generate a specification chart in base R. Code repository. GitHub. https://github.com/ArielOrtizBobea/spec_chart.

40. Young, C., and Holsteen, K. (2021). MULTIVRS: Stata Module to Conduct Multiverse Analysis. Statistical Software Component S458927 (Boston College Department of Economics). https://ideas.repec.org/c/boc/bocode/s458927.html.

41. Young, C., and Holsteen, K. (2017). Model Uncertainty and Robustness: A Computational Framework for Multimodel Analysis. Socio. Methods Res. *46*, 3–40.

42. Castro, O., Bruneau, P., Sottet, J.-S., and Torregrossa, D. (2024). Landscape of high-performance Python to develop data science and machine learning applications. ACM Computing Surveys, 56(3), Article *65*, 1–30. https://doi.org/10.1145/3617588.

43. Liu, Y., Kale, A., Althoff, T., and Heer, J. (2021). Boba: Authoring and Visualizing Multiverse Analyses. IEEE Trans. Vis. Comput. Graph. *27*, 1753–1763.

44. Barron, A., Birgé, L., and Massart, P. (1999). Risk bounds for model selection via penalization. Probab. Theor. Relat. Field *113*, 301–413.

45. Chen, X. (2007). Large Sample Sieve Estimation of Semi-Nonparametric Models. In Handbook of Econometrics, *76*, J.J. Heckman and E.E. Leamer, eds. (Elsevier), pp. 5549–5632. https://doi.org/10.1016/S1573-4412(07)06076-4.

46. Nelder, J.A., and Wedderburn, R.W.M. (1972). Generalized Linear Models. J. Roy. Stat. Soc. *135*, 370–384.

47. Aldrich, J.H., and Nelson, F.D. (1984). Linear Probability, Logit, and Probit Models (Sage).

48. Acemoglu, D., Johnson, S., and Robinson, J.A. (2001). The Colonial Origins of Comparative Development: An Empirical Investigation. Am. Econ. Rev. *91*, 1369–1401.

49. Vandaele, W. (1987). Participation in Illegitimate Activities: Ehrlich Revisited, 1960 (Version v1). In ICPSR - Interuniversity Consortium for Political and Social Research. https://doi.org/10.3886/ICPSR08677.v1.

50. Mankiw, N.G., Romer, D., and Weil, D.N. (1992). A Contribution to the Empirics of Economic Growth. Q. J. Econ. *107*, 407–437.

51. Zhang, Y., Bennett, M.R., and Yeandle, S. (2021). Longitudinal Analysis of Local Government Spending on Adult Social Care and Carers' Subjective Well-Being in England. BMJ Open *11*, e049652.

52. Dawber, T.R., Meadors, G.F., Moore, F.E., Jr, and Felix, E. (1951). Epidemiological Approaches to Heart Disease: The Framingham Study. Am. J. Public Health Nation's Health *41*, 279–286.

53. (2023). Retraction of Gino et al. (2020). J. Pers. Soc. Psychol. *125*, 648. https://doi.org/10.1037/pspa0000351.

54. Orben, A., and Przybylski, A.K. (2019). The Association Between Adolescent Well-Being and Digital Technology Use. Nat. Hum. Behav. *3*, 173–182.

55. U.S. Bureau of Labor Statistics (1988). National Longitudinal Survey of Mature and Young Women (NLSW) [Data Set]. Data set. U.S. Bureau of Labor Statistics. https://www.bls.gov/nls/home.htm.

56. Hirsch, B.T. (2004). Reconsidering Union Wage Effects: Surveying New Evidence on an Old Topic. Journal of Labor Research *25*, 233–266.

57. Ehrlich, I. (1973). Participation in Illegitimate Activities: A Theoretical and Empirical Investigation. J. Polit. Econ. *81*, 521–565.

58. Feenstra, R.C., Inklaar, R., and Timmer, M.P. (2015). The Next Generation of the Penn World Table. Am. Econ. Rev. *105*, 3150–3182.

59. ISER (2025). Understanding Society: Waves 1–14, 2009–2023 and Harmonised BHPS: Waves 1–18, 1991–2009 [Data Set], 19th edition (Data Collection. UK Data Service).

60. Wilson, P.W.F., D'Agostino, R.B., Levy, D., Belanger, A.M., Silbershatz, H., and Kannel, W.B. (1998). Prediction of Coronary Heart Disease Using Risk Factor Categories. Circulation *97*, 1837–1847.

61. D'Agostino, R.B., Vasan, R.S., Pencina, M.J., Wolf, P.A., Cobain, M., Massaro, J.M., and Kannel, W.B. (2008). General Cardiovascular Risk Profile for Use in Primary Care: The Framingham Heart Study. Circulation *117*, 743–753.

62. Gino, F., Kouchaki, M., and Casciaro, T. (2020). Why Connect? Moral Consequences of Networking with a Promotion or Prevention Focus. J. Pers. Soc. Psychol. *119*, 1221–1238. https://doi.org/10.1037/pspa0000226.

63. Simmons, J. (2024). Harvard's Gino Report Reveals How a Dataset Was Altered. Blog post. Data Colada. https://datacolada.org/118.

64. Harvard Business School Investigation Committee (2024). Harvard Report on Gino (Investigation Report Harvard Business School).

65. Auspurg, K. (2025). Robustness is better assessed with a few thoughtful models than with billions of regressions. Proceedings of the National Academy of Sciences *122* (43), e2521917122. https://doi.org/10.1073/pnas.2521917122.

66. Ganslmeier, M., and Vlandas, T. (2025). Reply to Auspurg: On the limits of "justified" model spaces. Proc. Natl. Acad. Sci. USA *122*, e2523374122.

67. Tierney, L., and Kadane, J.B. (1986). Accurate Approximations for Posterior Moments and Marginal Densities. J. Am. Stat. Assoc. *81*, 82–86.

68. Strubell, E., Ganesh, A., and McCallum, A. (2019). Energy and Policy Considerations for Deep Learning in NLP. In Proceedings of the 57th Annual Meeting of the Association for Computational Linguistics, pp. 3645–3650. https://doi.org/10.18653/v1/P19-1355.

69. Akaike, H. (1974). A New Look at the Statistical Model Identification. IEEE Trans. Automat. Control *19*, 716–723. https://doi.org/10.1109/TAC.1974.1100705.

70. Schwarz, G. (1978). Estimating the Dimension of a Model. Ann. Stat. *6*, 461–464. https://doi.org/10.1214/aos/1176344136.

71. Hannan, E.J., and Quinn, B.G. (1979). The Determination of the Order of an Autoregression. J. Roy. Stat. Soc. B *41*, 190–195. https://doi.org/10.1111/j.2517-6161.1979.tb01072.x.

72. Salganik, M.J., Lundberg, I., Kindel, A.T., Ahearn, C.E., Al-Ghoneim, K., Almaatouq, A., Altschul, D.M., Brand, J.E., Carnegie, N.B., Compton, R.J., et al. (2020). Measuring the predictability of life outcomes with a scientific mass collaboration. Proc. Natl. Acad. Sci. USA *117*, 8398–8403.

73. Brier, G.W. (1950). Verification of Forecasts Expressed in Terms of Probability. Mon. Weather Rev. *78*, 1–3.

74. Domingue, B.W., Rahal, C., Faul, J., Freese, J., Kanopka, K., Rigos, A., Stenhaug, B., and Tripathi, A.S. (2025). The Intermodel Vigorish (IMV) as a Flexible and Portable Approach for Quantifying Predictive Accuracy With Binary Outcomes. PLoS One *20*, e0316491. https://doi.org/10.1371/journal.pone.031649.

75. Domingue, B.W., Kanopka, K., Kapoor, R., Pohl, S., Chalmers, R.P., Rahal, C., and Rhemtulla, M. (2024). The Intermodel Vigorish as a Lens for Understanding (and Quantifying) the Value of Item Response Models for Dichotomously Coded Items. Psychometrika *89*, 1034–1054. https://doi.org/10.1007/s11336-024-09977-2.

76. Zhang, L., Rahal, C., Kanopka, K., Ulitzsch, E., Zhang, Z., and Domingue, B.W. (2026). Evaluating Model Predictive Performance in Confirmatory Factor Analysis with Binary Outcomes Using the InterModel Vigorish. Multivariate Behav. Res. 1–20. https://doi.org/10.1080/00273171.2026.2645212.

Patterns, Volume 7

Supplemental information

RobustiPy: An efficient next-generation multiversal library with model selection, averaging, resampling, and explainable AI

Daniel Valdenegro, Jiani Yan, Duiyi Dai, and Charles Rahal

RobustiPy: An efficient next-generation multiversal library with model selection, averaging, resampling, and explainable AI

Daniel Valdenegro Ibarra[1,3,5], Jiani Yan[1,2,3,4], Duiyi Dai[1,3], and Charles Rahal[1,3,5]

[1]Leverhulme Centre for Demographic Science, University of Oxford
[2]Department of Sociology, University of Oxford
[3]ESRC Centre for Care, University of Oxford
[4]Wolfson College, University of Oxford
[5]Nuffield College, University of Oxford

June 30, 2026

Keywords: *Multiverse analysis*; *Specification curve analysis*; *Robustness analysis*; *Model uncertainty quantification*; *Model selection*; *Bayesian model averaging*; *Bootstrap resampling*; *Joint inference*; *Out-of-sample validation*; *Explainable AI / SHAP*.

For correspondence: Daniel Valdenegro Ibarra, Jiani Yan, and Charles Rahal, Leverhulme Centre for Demographic Science and the Centre for Care, Oxford Population Health, University of Oxford, OX1 1JD, United Kingdom. Tel: 01865 286170. Email: daniel.valdenegro@demography.ox.ac.uk, jiani.yan@demography.ox.ac.uk and charles.rahal@demography.ox.ac.uk. The authors declare no conflicts of interest and are grateful to comments on earlier versions of the work from participants at the International Conference on Computational Social Science, the British Society for Population Studies, the European Consortium for Sociological Research, Population Association of America, Tsinghua University, the International Conference on Social Computing, and internal feedback received from the Leverhulme Centre for Demographic Science, Reproducible Research Oxford, and the Pandemic Sciences Institute. We also thank all attendees at a previous hackathon, hosted at the University of Oxford in June 2024. We especially thank Anda-Raluca Epure for detailed comments on the manuscript and an extensive code review of the accompanying software. The authors are grateful for funding from the ESRC (an ESRC Large Centre grant), the Leverhulme Trust (Grant RC-2018-003) for the Leverhulme Centre for Demographic Science and Nuffield College, and a Grand Union DTP ESRC studentship.

Supplementary Figures

Supplementary Figure S1: Array of never-changing predictors

```
import numpy as np
import pandas as pd
from robustipy.models import OLSRobust


def sim2(project_name):
    np.random.seed(192735)
    beta1 = np.array([0.2, 0.5, -0.4, -0.7, 0.2,
                      0.5, 0.2, 0.5, 0.3])
    L = np.array([
        [0.8, 0.2], [0.6, -0.5], [0.7, 0.1],
        [0.5, -0.6], [0.4, 0.7], [0.3, -0.4],
        [0.2, 0.3], [0.1, -0.2]])
    D = np.diag([5] * 8)
    cov_matrix = L.dot(L.T) + D
    num_samples = 1000
    mean_vector = np.zeros(8)
    X = np.random.multivariate_normal(mean=mean_vector,
                                      cov=cov_matrix,
                                      size=num_samples)
    X_i = np.column_stack((np.ones(num_samples), X))
    errors = np.random.normal(0.0, 1.0, num_samples)
    Y1 = np.dot(X_i, beta1) + errors
    np_data = np.column_stack((Y1, X))
    data = pd.DataFrame(np_data, columns=['y1', 'x1', 'z1', 'z2',
                                          'z3', 'z4', 'z5', 'z6',
                                          'z7'])
    y = ['y1']
    x = ['x1', 'z1']
    z = ['z2', 'z3','z4', 'z5', 'z6', 'z7']
    sim2 = OLSRobust(y=y, x=x, data=data)
    sim2.fit(controls=z, draws=1000, kfold=10, seed=192735)
    sim2_results = sim2.get_results()
    sim2_results.plot(specs=[['z4', 'z5']],
                      ic='hqic', figsize=(16, 16),
                      ext='pdf',
                      project_name=project_name)
    sim2_results.summary()


if __name__ == "__main__":
    sim2('sim2_example')
```

Supplementary Figure S2: Fixed Effect OLS

```
import numpy as np
import pandas as pd
from robustipy.models import OLSRobust


def sim3(project_name):
    np.random.seed(192735)
    beta_mean = [0.9, 0.4, -0.7, -0.1, 0.2, 0.3, -0.2, 0.5, 0.1]
    beta_std = 2
    L_factor = np.array([[0.8,  0.2], [0.6, -0.5],
                         [0.7,  0.1], [0.5, -0.6],
                         [0.4,  0.7], [0.3, -0.4],
                         [0.2,  0.3], [0.1, -0.2]])
    D = np.diag([0.3] * 8)
    cov_matrix = L_factor.dot(L_factor.T) + D
    num_samples, num_groups = 10, 1000
    mean_vec = np.zeros(8)
    X_list, Y_list, group_list = [], [], []
    for group in range(1, num_groups + 1):
        beta = np.random.normal(beta_mean, beta_std, len(beta_mean))
        mi, ma, factor = np.argmin(beta), np.argmax(beta), 5
        beta[mi] = np.random.normal(beta[mi], beta_std * factor)
        beta[ma] = np.random.normal(beta[ma], beta_std * factor)
        X = np.random.multivariate_normal(mean_vec,
                                          cov_matrix,
                                          num_samples) \
            + np.random.normal(0, 5, (num_samples, 8))
        X_i = np.column_stack((np.ones(num_samples), X))
        errors = np.random.normal(0, 20, num_samples)
        Y = np.dot(X_i, beta) + errors
        X_list.append(X)
        Y_list.append(Y)
        group_list.extend([group] * num_samples)
    data = pd.DataFrame(np.column_stack((np.concatenate(Y_list),
                                         np.vstack(X_list))),
                        columns=['y', 'x1', 'z1', 'z2', 'z3',
                                 'z4', 'z5', 'z6', 'z7'])
    data['group'] = group_list
    sim3 = OLSRobust(y=['y'], x=['x1'], data=data)
    sim3.fit(controls=['z1', 'z2', 'z3', 'z4', 'z5', 'z6', 'z7'],
             group='group', draws=1000, kfold=10,
             rescale_y=True, rescale_z=True,
             seed=192735)
    sim3_results = sim3.get_results()
    sim3_results.plot(specs=[['z1', 'z2', 'z3']], ic='hqic',
                      figsize=(16, 16), ext='pdf',
                      project_name=project_name)
    sim3_results.summary()


if __name__ == '__main__':
    sim3('sim3_example')
```

Supplementary Figure S3: Logistic Regression

```python
import numpy as np
import pandas as pd
from robustipy.models import LRobust

def sim4(project_name):
    np.random.seed(192735)
    beta1 = np.array([0.05, 0.1, -0.6, -0.35, 0.05,
                      0.1, 0.05, 0.1, 0.05])
    L_factor = np.array([
        [0.8,  0.2],
        [0.6, -0.5],
        [0.7,  0.1],
        [0.5, -0.6],
        [0.4,  0.7],
        [0.3, -0.4],
        [0.2,  0.3],
        [0.1, -0.2]
    ])
    D = np.diag([0.3]*8)
    cov_matrix = L_factor.dot(L_factor.T) + D
    num_samples = 10000
    mean_vector = np.zeros(8)
    X = np.random.multivariate_normal(mean=mean_vector,
                                      cov=cov_matrix,
                                      size=num_samples)
    X_i = np.column_stack((np.ones(num_samples), X))
    errors = np.random.normal(0.0, 1, num_samples)
    Y1 = np.dot(X_i, beta1) + errors
    threshold = np.median(Y1)
    Y1 = (Y1 > threshold).astype(int)
    np_data = np.column_stack((Y1, X))
    data = pd.DataFrame(np_data, columns=['y1','x1','z1','z2',
                                          'z3','z4','z5','z6','z7'])

    y = ['y1']
    x = ['x1']
    z = ['z1','z2','z3','z4','z5','z6','z7']
    sim4 = LRobust(y=y, x=x, data=data)
    sim4.fit(controls=z, draws=1000, kfold=10, seed=192735)
    sim4_results = sim4.get_results()
    sim4_results.plot(specs=[['z1'], ['z2','z4'],
                             ['z3','z5']],
                      ic='hqic', figsize=(16,16),
                      ext='pdf', project_name=project_name)
    sim4_results.summary()


if __name__ == "__main__":
    sim4('sim4_example')
```

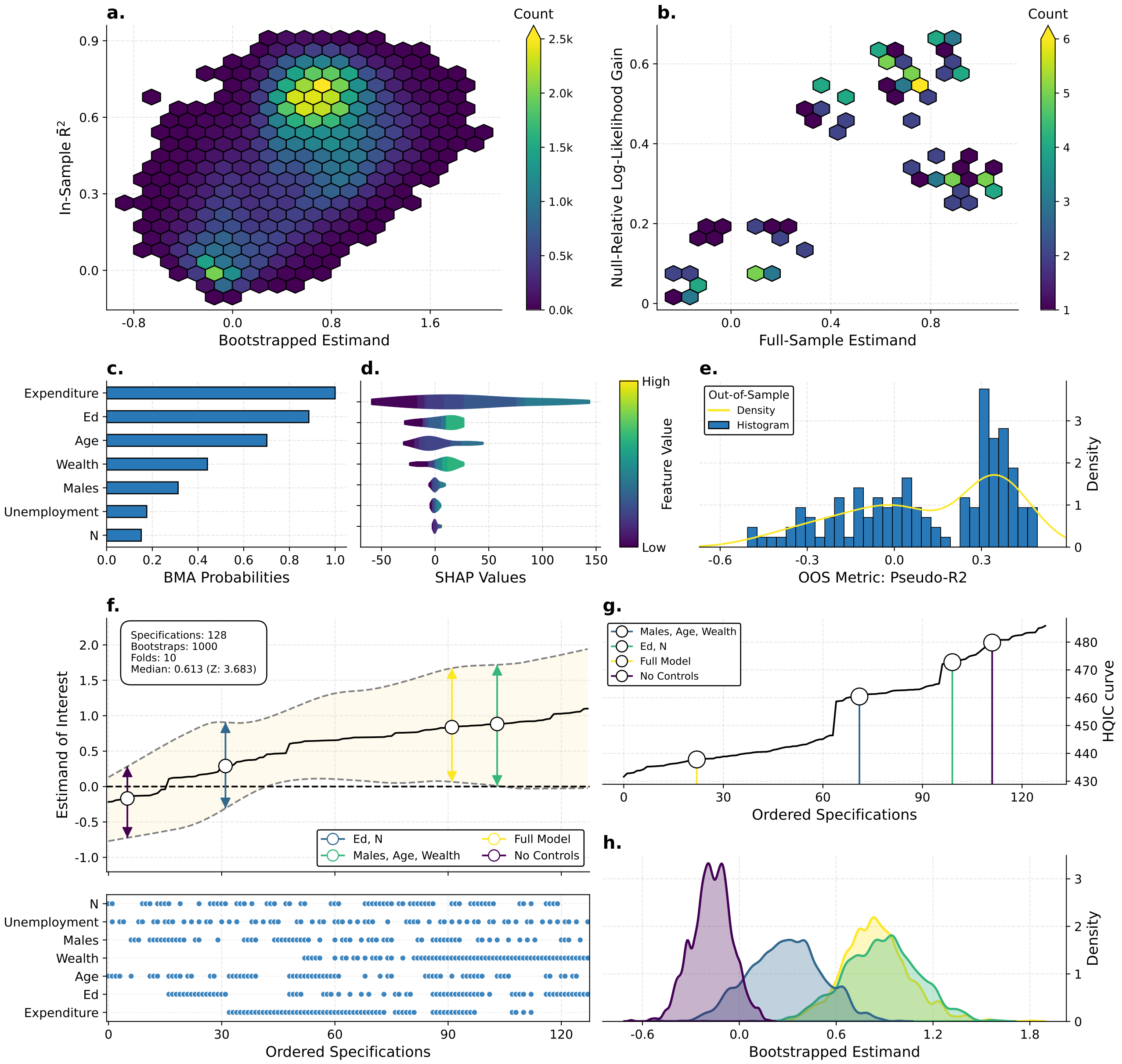

Supplementary Figure S4: An application of `RobustiPy` to criminology: Ehrlich (1973) revisited, based on a revised dataset from Vandaele (1987). The dependent variable is state-level crime rate, while the estimand of interest is income inequality.

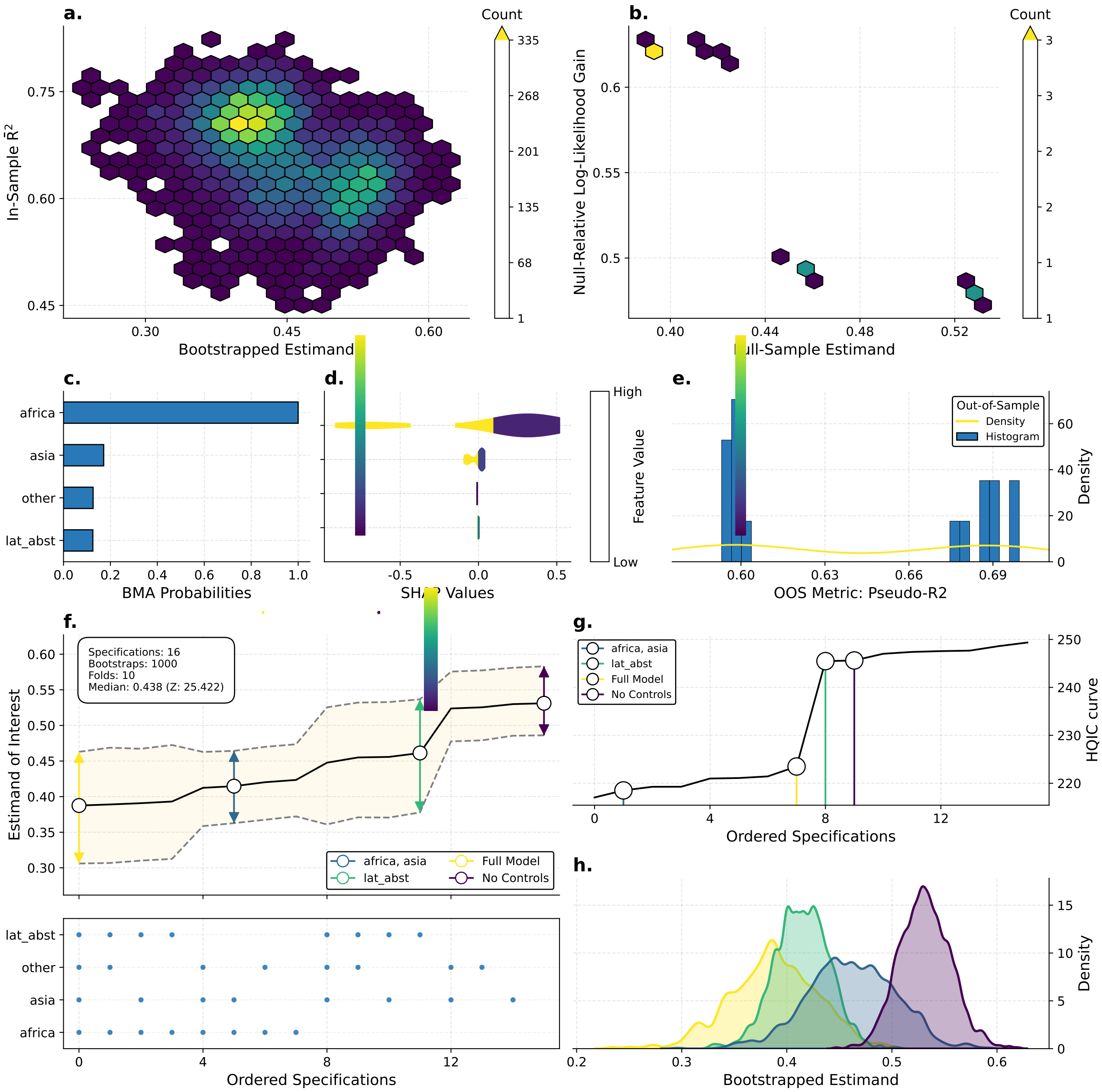

Supplementary Figure S5: A re-examination of the influential Acemoglu et al. (2001) paper. Our analysis spans from the core, baseline OLS model, to the fullest OLS model as specified in Table 2 of the aforementioned paper, where the variation of all intermediate specifications are analysed by `RobustiPy`. The baseline models without any and with all controls are highlighted in subfigures '**f.**', '**g.**' and '**h.**'.

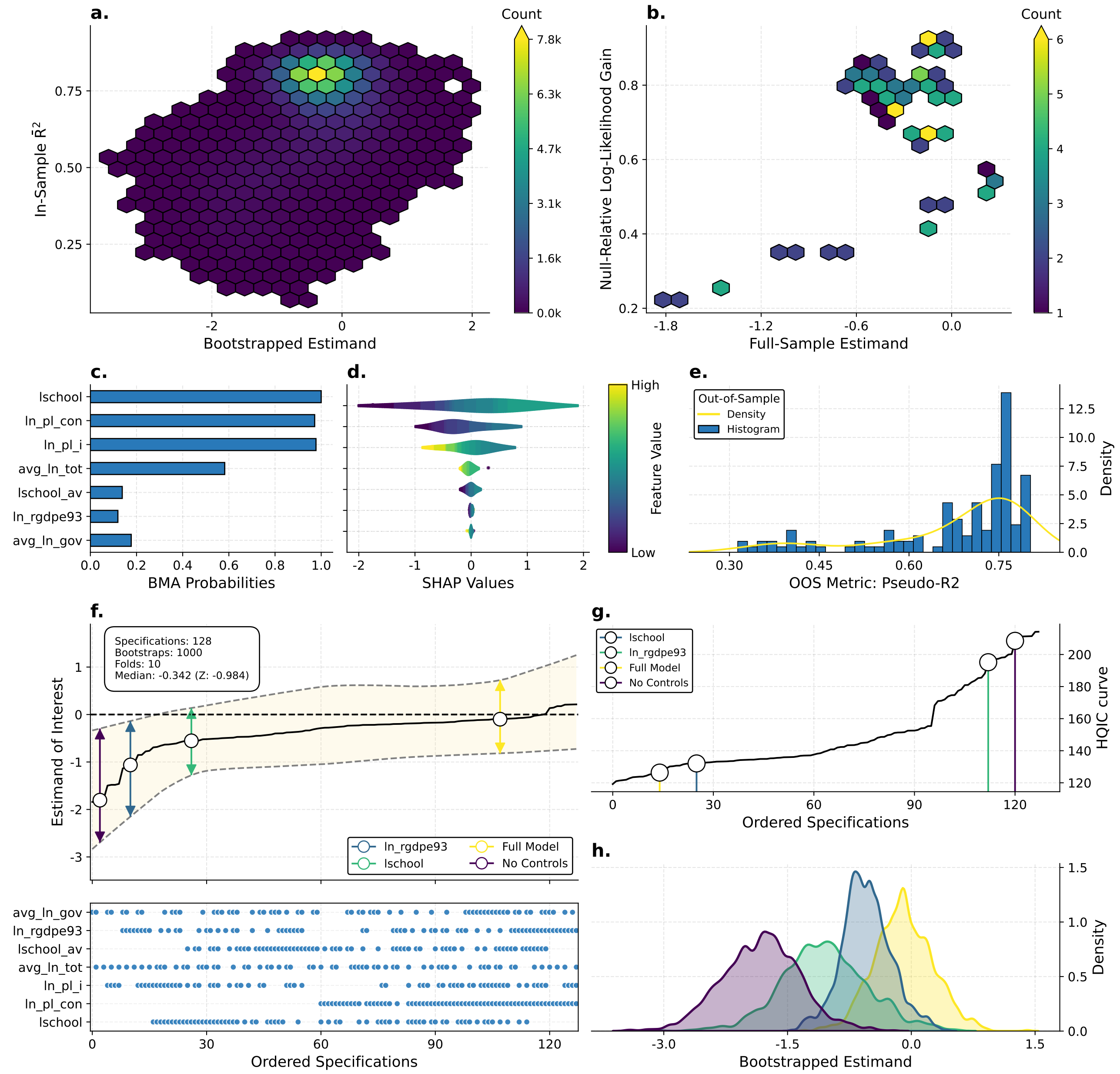

Supplementary Figure S6: A re-analysis of the Solow Growth Model as per Mankiw et al. (1992). With this instantiation of `RobustiPy`, we order the logarithm of (n+g+δ) – averaged over the preceding years and empirically calibrated for all countries – first in our x array.

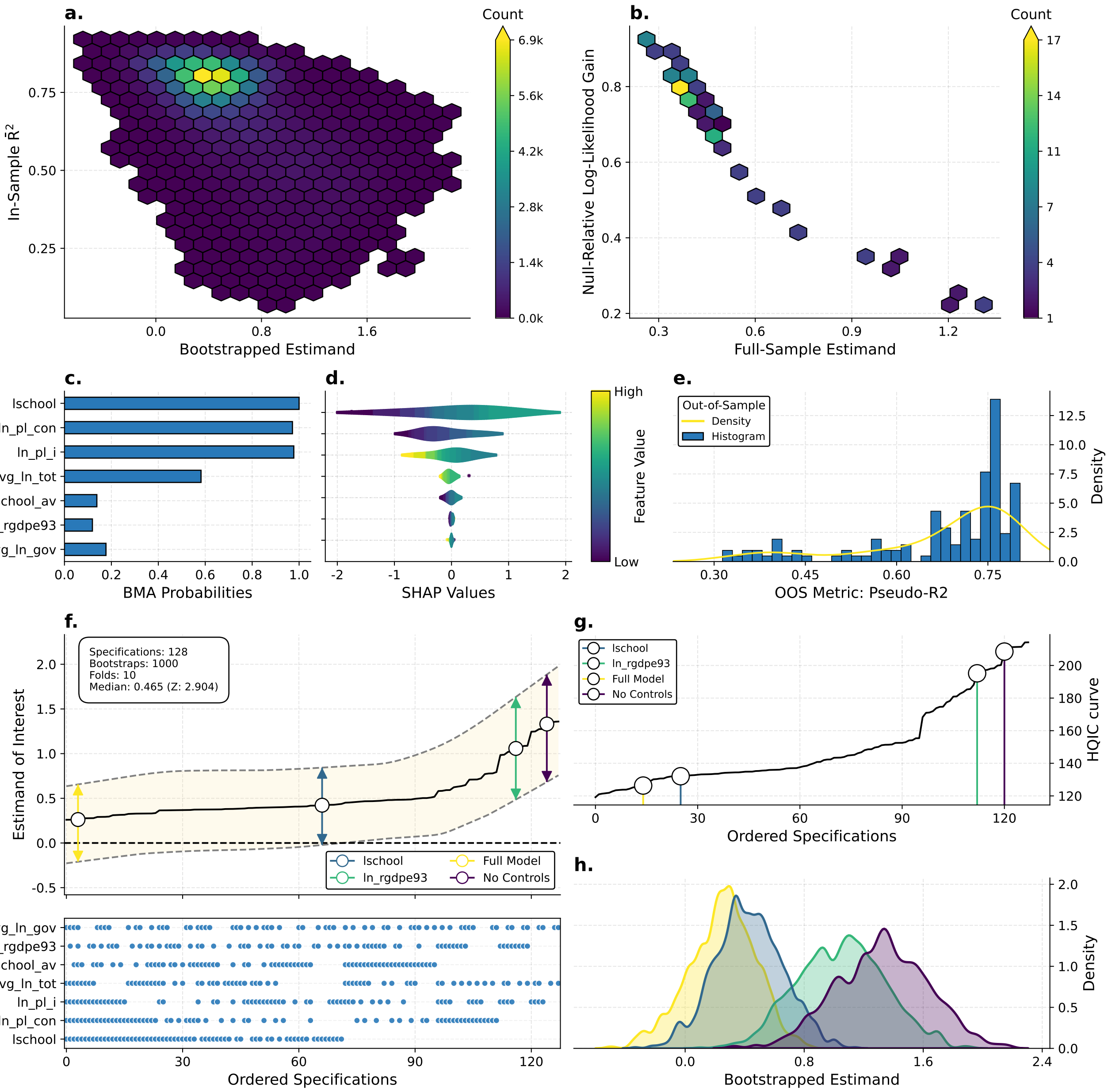

Supplementary Figure S7: A re-analysis of the Solow Growth Model as per Mankiw et al. (1992). With this instantiation of `RobustiPy`, we order the logarithm of $\frac{I}{Y}$ – averaged over the preceding years and empirically calibrated for all countries – first in our x array.

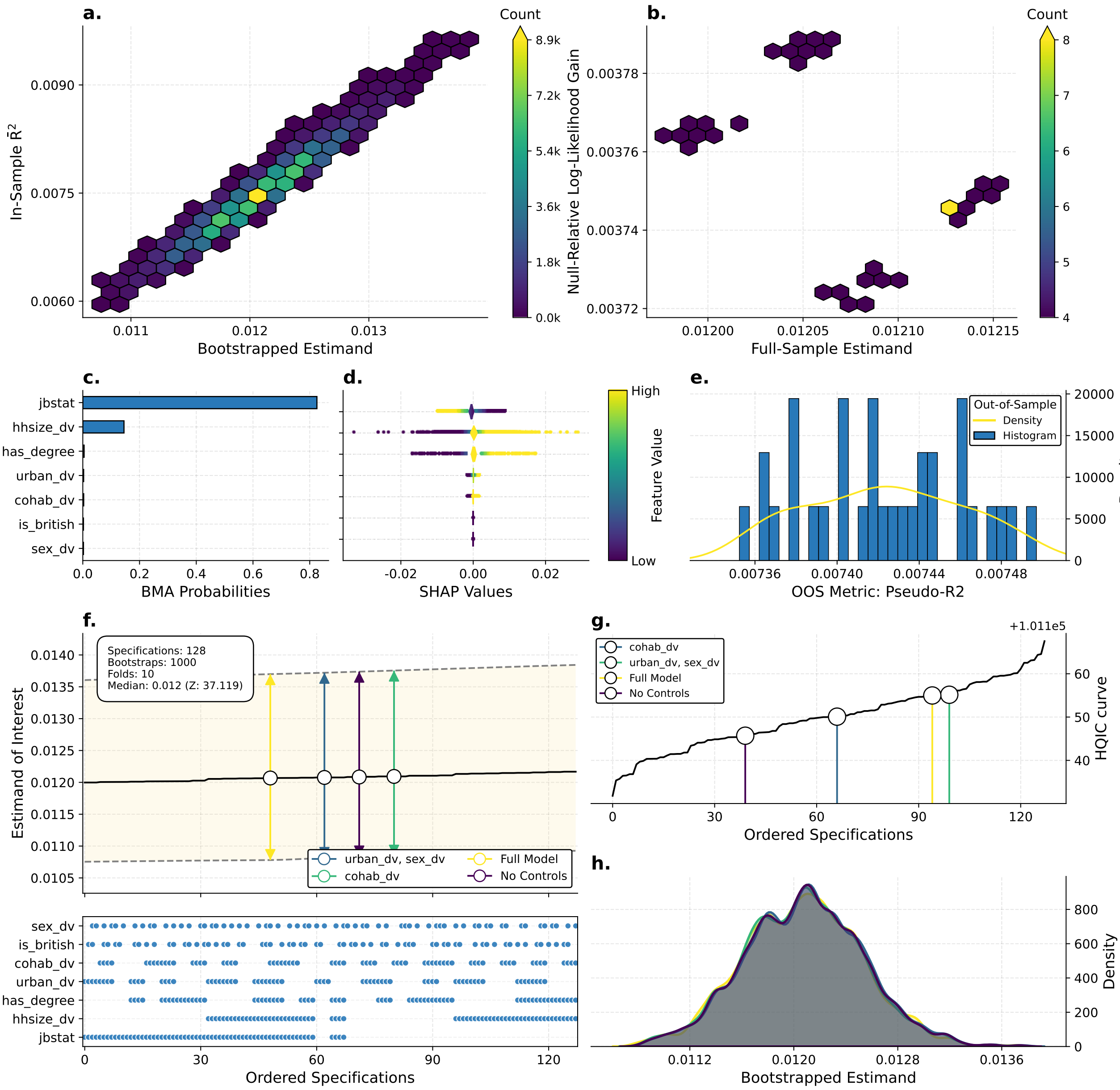

Supplementary Figure S8: Example output of `RobustiPy` **utilising the UKHLS dataset**. The dependent variable is a binary representation of whether a survey recipient is in 'good health', estimated as a linear probability model. This example showcases individuals grouped longitudinally via the `group` input to the '`.fit()`' method (in this case, with `group='pipd'`).

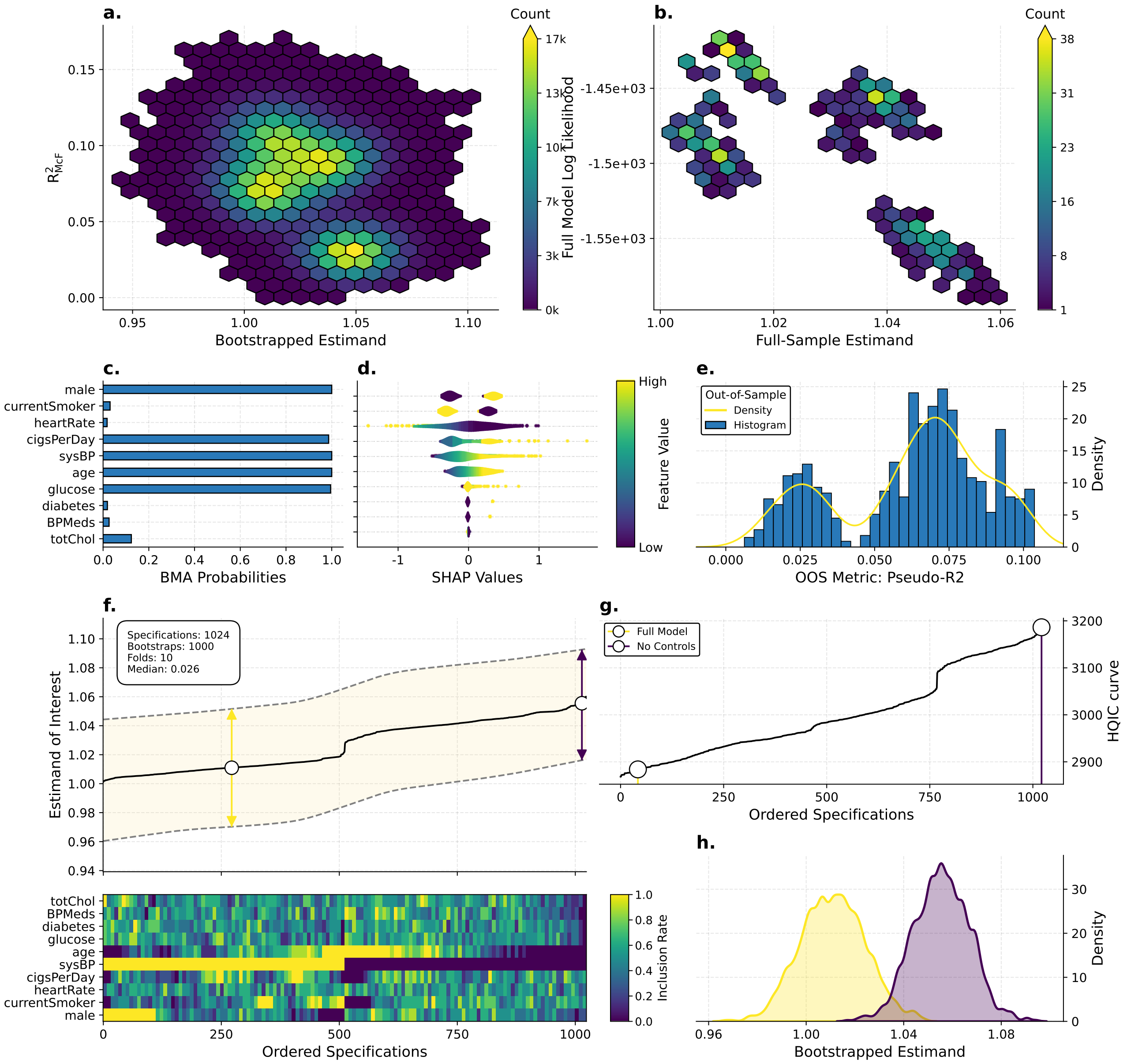

Supplementary Figure S9: Example output of `RobustiPy` with a binary dependent variable. The dependent variable is a ten year metric of coronary heart disease (CHD), and the variable of interest is Body Mass Index.

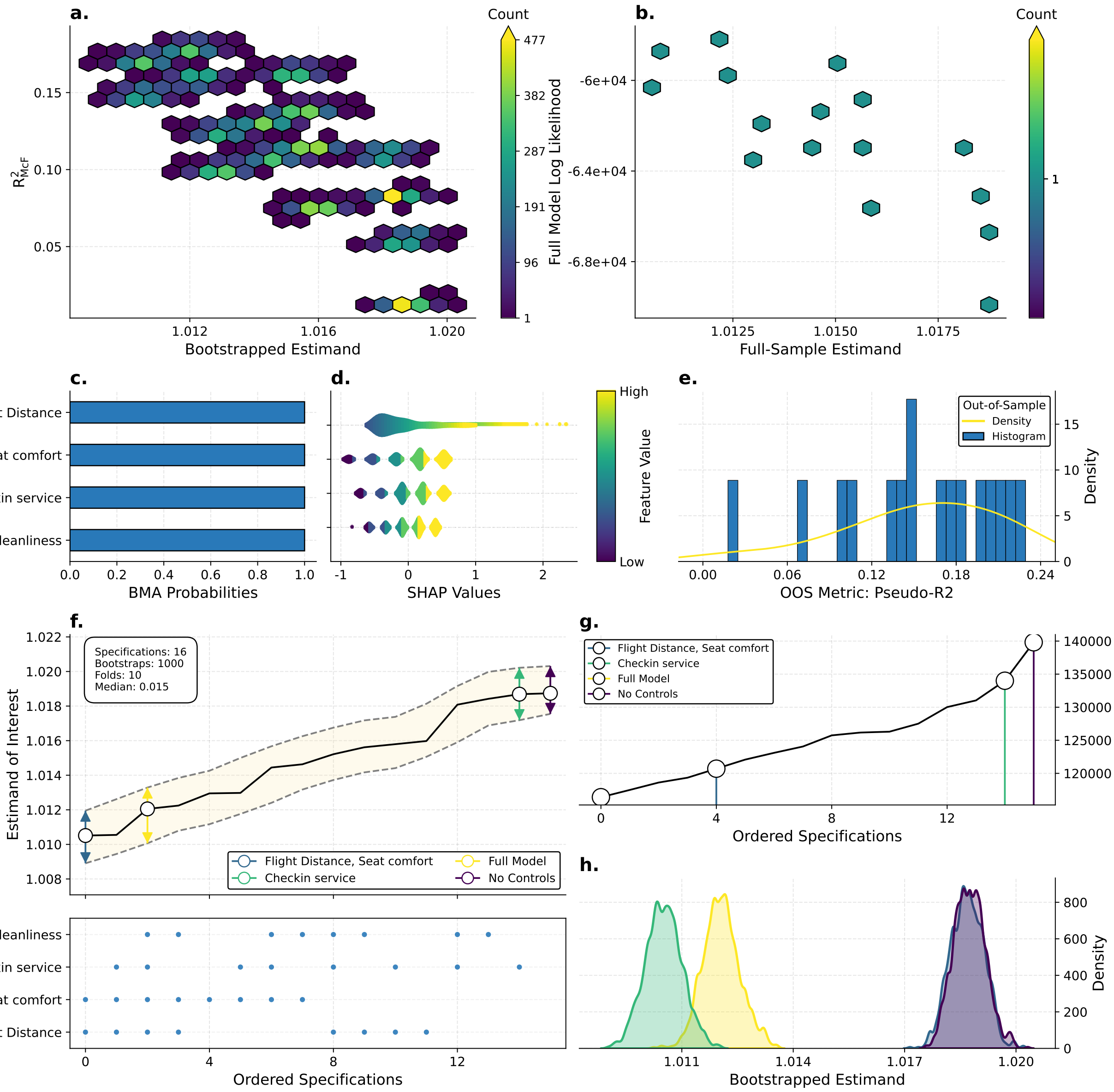

Supplementary Figure S10: Example output of RobustiPy utilising a binary dependent variable. The dependent variable is a representation of custom satisfaction within an anonymous airline company, with data provided by Kaggle. The estimand of interest relates to the age of the customer.

Supplementary References

Daron Acemoglu, Simon Johnson, and James A. Robinson. The colonial origins of comparative development: An empirical investigation. *American Economic Review*, 91(5):1369–1401, 2001.

Isaac Ehrlich. Participation in illegitimate activities: A theoretical and empirical investigation. *Journal of Political Economy*, 81(3):521–565, 1973.

N. Gregory Mankiw, David Romer, and David N. Weil. A contribution to the empirics of economic growth. *Quarterly Journal of Economics*, 107(2):407–437, 1992.

Walter Vandaele. *Participation in Illegitimate Activities: Ehrlich Revisited, 1960 [Data Set]*. Inter-University Consortium for Political and Social Research, 1987.
